# Bioinformatic identification of novel putative photoreceptor specific *cis*-elements

**DOI:** 10.1186/1471-2105-8-407

**Published:** 2007-10-22

**Authors:** Charles G Danko, Vera A McIlvain, Maochun Qin, Barry E Knox, Arkady M Pertsov

**Affiliations:** 1 Department of Pharmacology, SUNY Upstate Medical University, Syracuse, NY, USA; 2 Department of Biochemistry & Molecular Biology and Ophthalmology, SUNY Upstate Medical University, Syracuse, NY, USA

## Abstract

**Background:**

Cell specific gene expression is largely regulated by different combinations of transcription factors that bind *cis*-elements in the upstream promoter sequence. However, experimental detection of *cis*-elements is difficult, expensive, and time-consuming. This provides a motivation for developing bioinformatic methods to identify *cis*-elements that could prioritize future experimental studies. Here, we use motif discovery algorithms to predict transcription factor binding sites involved in regulating the differences between murine rod and cone photoreceptor populations.

**Results:**

To identify highly conserved motifs enriched in promoters that drive expression in either rod or cone photoreceptors, we assembled a set of murine rod-specific, cone-specific, and non-photoreceptor background promoter sequences. These sets were used as input to a newly devised motif discovery algorithm called Iterative Alignment/Modular Motif Selection (IAMMS). Using IAMMS, we predicted 34 motifs that may contribute to rod-specific (19 motifs) or cone-specific (15 motifs) expression patterns. Of these, 16 rod- and 12 cone-specific motifs were found in clusters near the transcription start site. New findings include the observation that cone promoters tend to contain TATA boxes, while rod promoters tend to be TATA-less (exempting *Rho *and *Cnga1*). Additionally, we identify putative sites for IL-6 effectors (in rods) and RXR family members (in cones) that can explain experimental data showing changes to cell-fate by activating these signaling pathways during rod/cone development. Two of the predicted motifs (NRE and ROP2) have been confirmed experimentally to be involved in cell-specific expression patterns. We provide a full database of predictions as additional data that may contain further valuable information. IAMMS predictions are compared with existing motif discovery algorithms, DME and BioProspector. We find that over 60% of IAMMS predictions are confirmed by at least one other motif discovery algorithm.

**Conclusion:**

We predict novel, putative *cis-*elements enriched in the promoter of rod-specific or cone-specific genes. These are candidate binding sites for transcription factors involved in maintaining functional differences between rod and cone photoreceptor populations.

## Background

Experimental identification of DNA sequence motifs that bind specific transcription factors (*cis*-elements) and regulate gene expression are expensive, time-consuming, and difficult. This makes bioinformatic methods for identifying *cis*-elements an important tool for prioritizing future experimental studies of transcriptional regulation. Rod and cone photoreceptors each specialize in a unique function by the expression of distinct genes that perform analogous roles in each cell's light transduction pathway. Bioinformatic motif identification techniques have been used to successfully identify potential targets of 3 photoreceptor-specific transcription factors (NRL, CRX, NR2E3) using their known binding specificity [1]. Experimental evidence suggests that at least 9 additional transcription factors are involved in regulation of either rod- or cone-specific expression [2]. However, binding motifs for many of these transcription factors are presently unknown. In this study, we use *de novo *motif discovery methods to identify motifs that may be important for gene expression differences between rod and cone photoreceptors.

The most commonly used *de novo *method is phylogenetic footprinting, based on the assumption that functional sequence changes more slowly through evolution compared to the surrounding sequence. The advantage of phylogenetic footprinting is its specificity: significant conservation across many species strongly suggests that a sequence is functional. However, phylogenetic footprinting suffers from a high incidence of false negative errors [3-6]. Alternative approaches seek to identify motifs that are over-represented compared to a set of unrelated background sequences [7,8]. To increase the accuracy of predictions, recent over-representation motif discovery implementations incorporate additional biological information [9-11], such as the position of motifs relative to the transcription start site (for reviews see: [12,13]). Here, we use a combination of over-representation, position-based filtering, and phylogenetic analysis to select and analyze motifs that may be involved in rod and cone-specific expression patterns.

Our motif discovery implementation, called iterative alignment/modular motif selection (IAMMS), selects motifs based on three biological assumptions. First, we assume that promoters of functionally linked genes will share similar regulatory motifs. The second assumption is that functional motifs are concentrated near the transcription start site [14]. Third, we assume that occurrences of a given motif cluster near a characteristic distance from the transcription start site [14]. To implement the last two assumptions, we applied a hierarchical clustering algorithm because the algorithm chooses the mode and variance of a distribution based on the underlying data. This approach advances position-based filtering over previous implementations that model motif position dependence by a static distribution given by the empirical frequency of all motifs relative to the transcription start site in bacteria [12]. We implement this approach on a set of murine rod-specific, cone-specific, and background promoter sequences derived from biochemical [15-21] and microarray [2,22] studies.

IAMMS identified 34 motifs enriched in the promoter of either rod or cone photoreceptors, most of which are not similar to any previously known motifs. To increase our confidence in these predictions, results obtained using IAMMS were compared to those of existing motif discovery algorithms, DME and BioProspector. We chose BioProspector because it improves on the well-studied Gibbs sampling algorithm by representing background sequences as a third-order Markov model [8,13]. DME was chosen because it is based on the well-regarded maximum likelihood algorithm [7]. This comparison revealed that over 60% of our predictions were also confirmed by at least one additional algorithm. We provide extensive discussion of these predictions in the context of the biochemical literature.

## Results

### Application of IAMMS to Rod and Cone-specific Promoters

**Figure 1:**
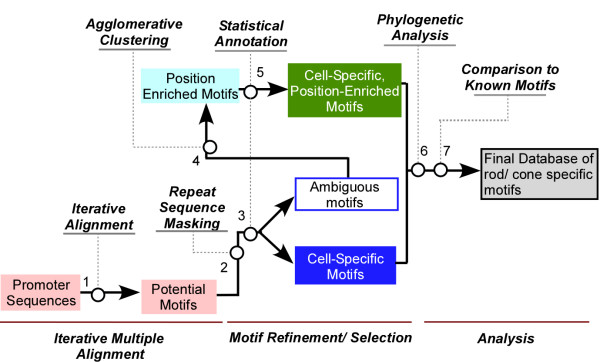
A block diagram of the iterative alignment/modular motif selection (IAMMS) algorithm used to identify putative functional sites in photoreceptor promoter regions. Boxes represent the input/output of each successive step. Arrows show flow. Circles show the application of a given filter.

**Figure 2:**
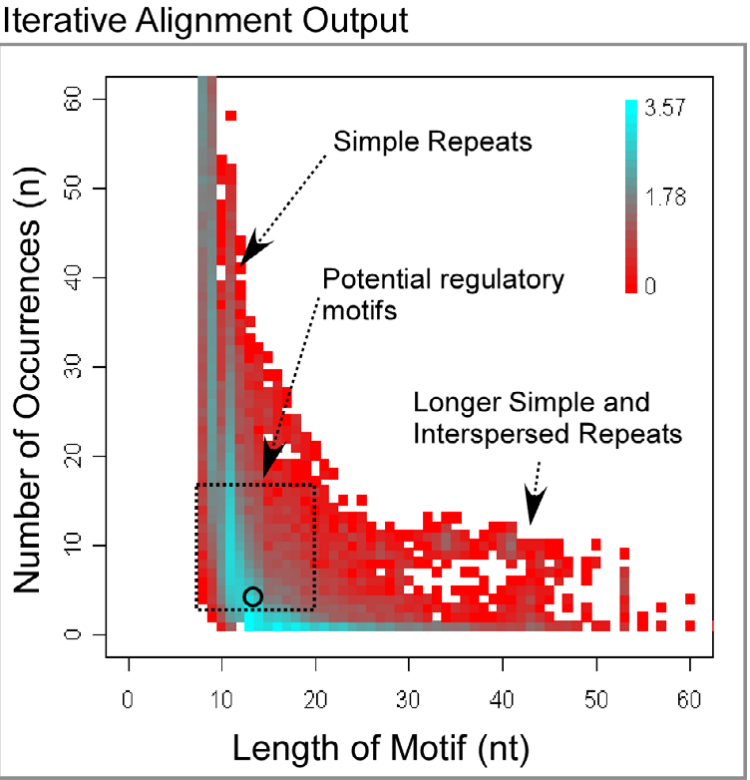
3D histogram representing features of potential motifs after the iterative alignment. The vertical and horizontal axis plot the number of non-overlapping occurrences of a motif, and the motif length in nucleotides (nt), respectively. Color shows the number (on a log-10 scale) of motifs with the given parameters. The box shows the approximate area that is likely to contain functional motifs. The circle shows the region containing the motif sample in Fig. 3A. Longer motifs (> 20 bp) are longer simple or interspersed repeats.

Input to IAMMS consisted of the upstream region of 11 rod-specific, 12 cone-specific, and 84 non-photoreceptor genes (see table 1 for a list of rod/cone-specific genes, and additional file 1 online for background genes). The flowchart of the IAMMS algorithm is shown in figure 1 (see methods for details). The first step involved an iterative alignment procedure conducted on all rod, cone, and non-photoreceptor promoters. This step resulted in a dataset of 71,195 conserved motifs between 8 and 150 bp in length. Each entry of the dataset contains nucleotide sequences, the location of motif occurrences with respect to the transcription start site, strand, and promoter from which each occurrence originated. To illustrate the composition of the dataset, we plotted motif length against the number of occurrences of each motif in photoreceptor promoters (figure 2; background occurrences are not shown). The color map represents the number of motifs with each length/frequency combination. As may be expected, motif size has an inverse relationship with the number of occurrences.

**Table 1:** Rod-specific and cone-specific genes

**Rod:**	Rho^22^; Sag^17^; Pde6a^22^; Pde6g^22^; Pde6d^20^; Pde6b^22^; Nrl^18^; Nr2e3^19^; Gnat1^22^; Cnga1^22^; Gnb1^15^
**Cone:**	Opn1mw^15^; Opn1sw^15^; Pde6c^15^; Pde6h^15^; Arr3^15^; Cngb3^15^; Cnga3^21^; Smug1^15^; Gnat2^15^; Gnb3^15^; Elovl2^15^; Gngt2^15^

Rod-specific and cone-specific genes whose promoters were used in this study are listed by MGI symbol. References to the article stating cell-specificity are given superscript to each gene.

Analysis showed that the majority of motifs identified after the first step were repeat sequences. The motifs occurring most frequently (> 25 occurrences) were primarily simple repeats. All longer motifs (> 19 bp) were highly similar to microsatellites and interspersed repeats, as revealed by comparison to a database of known repeats (RepBase). Repeat sequences were filtered out at step 2.

After repeat filtering, the remaining motifs, those inside and immediately above the marked box in figure 2, were evaluated for potential enrichment in rod or cone photoreceptors (step 3). Since we are interested in motifs that occur in the promoters of only one photoreceptor cell type, motifs that have occurrences in both rod and cone promoters were classified as ambiguous and were excluded from consideration during this step. To evaluate enrichment of a motif compared to background, we assume a binomial distribution of *k*_*r *_rod specific (or *k*_*c *_cone-specific) promoters drawn from the total number of promoters that contain occurrences. A Bonferroni correction for multiple hypothesis testing (E-value) is applied to the resulting p-value, as described in the *Statistical annotation *section of methods. The top scoring motifs identified during this step were subjected to phylogenetic analysis (step 6) and compared to known motifs using the Transcriptional Element Search System (TESS; step 7).

**Figure 3:**
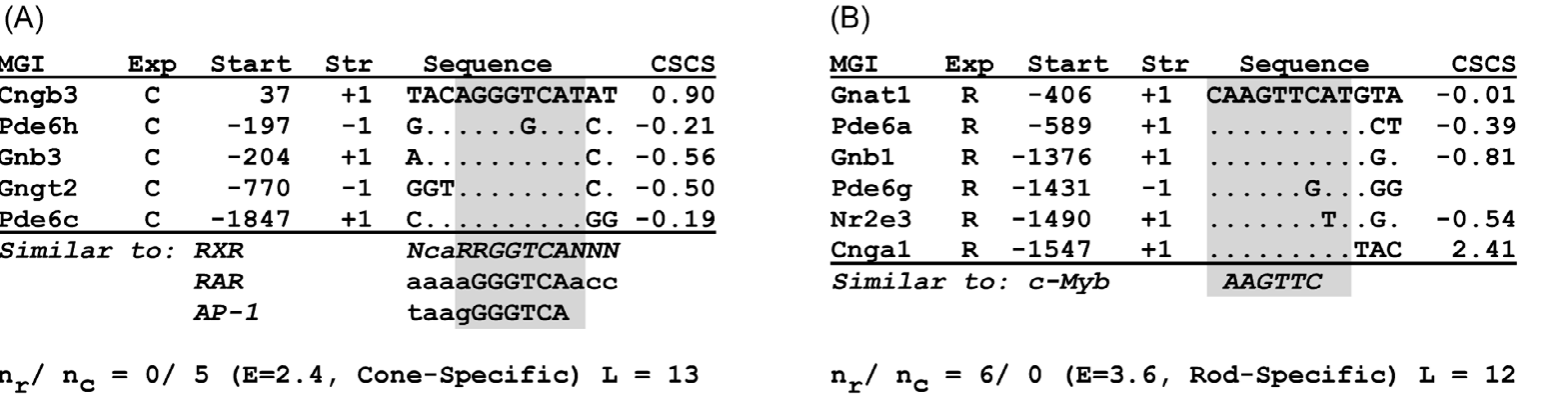
Example of cone (A) and rod (B) enriched DNA motifs after statistical annotation. Columns from left to right give gene MGI Symbol, cell-specific expression patterns (C, cone; R, rod; background matches are removed for this figure), start position of motif occurrence relative to the transcription start site, strand relative to the transcription start site (+1), consensus sequence (shown on the top), and cross-species conservation score (see methods). Occurrences are sorted by distance from transcription start site. The cone motif (A) is similar to a known binding site (RXR). The rod motif (B) is similar to the c-Myb binding site. For both motifs, non-photoreceptor occurrences (n = 2, 9 for A and B, respectively) have been removed for simplicity.

Figure 3 shows representative examples of top-scoring cone- and rod-enriched motifs identified during step 3, after being subjected to phylogenetic analysis, and compared to TESS. The cone-enriched motif is 13 bp in length, contains 5 occurrences in cone-specific promoters and none in rods (non-photoreceptor occurrences are not shown). The cross species conservation scores (CSCS) for each occurrence is shown in the last column. Four occurrences have a negative CSCS. A negative CSCS means that the predicted occurrence is more conserved than surrounding sequences of the same length (see Methods for details). Comparison with known photoreceptor-specific motifs indicated that this sequence is similar to the preferred binding site for the Retnoid X Receptor (RXR). Involvement of RXR in cone-specific expression is well established [2], but binding sites for this transcription factor in cone photoreceptor promoters have not yet been identified, making this prediction valuable for planning experimental studies.

The rod-enriched motif (figure 3B) is 12 bp in length and contains 6 occurrences in rod promoters. Cross-species conservation shows that *Pde6a*, *Gnb1*, and *Nr2e3 *occurrences are phylogenetically conserved (a cross-species alignment is not available for the region containing the *Pde6g *occurrence, and thus no score is reported). According to TESS, this motif is similar to a c-Myb binding site. The prediction that c-Myb may have a function unique to one type of photoreceptor is consistent with publicly available microarray data (see Methods). We found that c-Myb is between 2.6 and 7.6 fold enriched in cones compared to rod photoreceptors.

**Figure 4:**
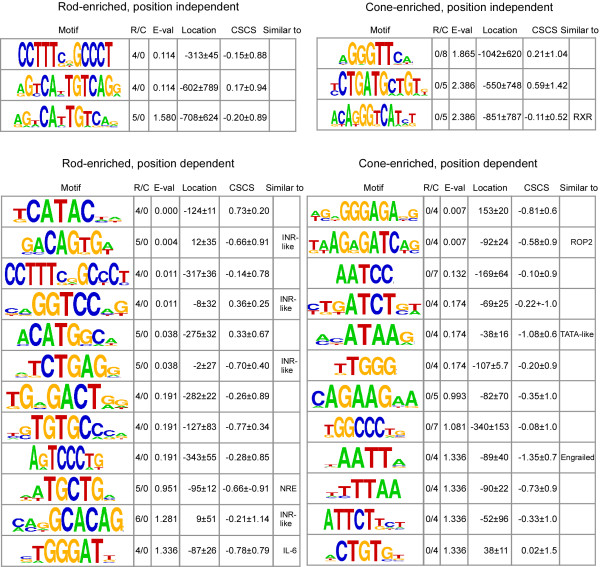
Highest scoring rod (left) and cone (right) enriched motifs returned after statistical annotation in IAMMS step 3 (position independent) and IAMMS step 5 (position dependant). From the left, columns give the motif logo, the fraction of rod/cone specific occurrences, cell-specificity E-value, mean location relative to the transcription start site (bp), mean phylogenetic conservation score, and similarity to known motifs. The table is sorted based on the fraction of cell-specific sequence (E-value). Note that predictions with similar core sequences are represented by the prediction with the highest E-value in figure 4. All predictions are presented individually in figure 7.

After step 3, IAMMS identified a total of 6 motifs (3 rod- and 3 cone-enriched) with E < 2.5. Since no position filtering was applied to identify these motifs, we refer to them as position independent. All position independent rod- and cone-enriched motifs, sorted based on E-value, are shown on the top of figure 4. The highest scoring rod prediction at the top of figure 4 contains two 5 bp invariant core regions separated by two ambiguous positions (CCTTTNNGCCCT; rod-enriched position independent, row 1). The position variance of this prediction is remarkably small (± 45) considering that no position-based selection was applied to identify this sequence. The top scoring cone motif contains a core region 5 bp in width (aGGGTTca). It occurs in 8/12 cone promoter sequences with no discernable bias in position. Detailed information on the position and phylogenetic conservation of each occurrence is available as additional data (files 1, 2) online.

**Figure 5:**
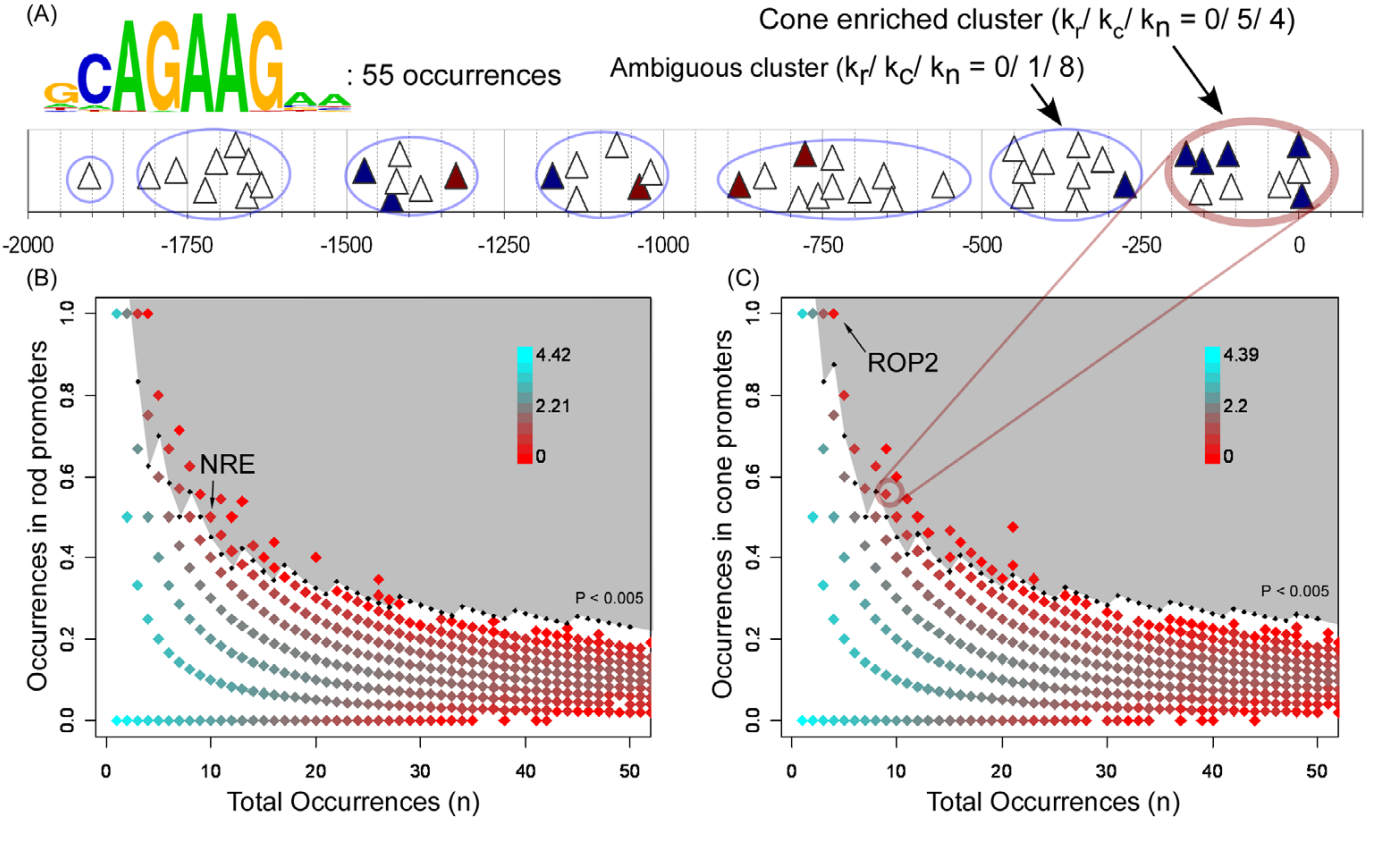
(A) Occurrences of a sample ambiguous motif (triangles) analyzed using position cluster discovery. The horizontal axis represents position relative to the putative transcription start site. The vertical position of occurrences was offset to ease viewing. Position clusters (ovals) were identified using agglomerative hierarchical clustering for all occurrences of each motif in the 2 kbp upstream region identified. Clusters with occurrences in the first 400 bp relative to the transcription start site were evaluated for cell-specificity. In this case, the cluster of occurrences nearest the transcription start site is cone-enriched. A second cluster between -250 and -500 is entirely ambiguous. The numbers k_r_, k_c_, and k_n _reflect the number of rod, cone, and background promoters that contain the motif. (B-C) Identification of cell-specific motifs among position-enriched clusters by statistical annotation. The vertical and horizontal axes plot the fraction of rod (B) or cone (C) promoters against the total number of promoters that contain at least one occurrence of a putative motif. Colors are assigned by the number of motifs with a given fraction (log-10 scale). The shaded region represents groups chosen using a p < 0.005 cutoff threshold.

Those motifs classified as ambiguous during step 3 were subjected to position-based clustering (step 4). As described previously, we acted under the hypothesis that occurrences of a motif near the transcription start site, and those occurring in clusters around a preferred position, are more likely to be functional. One example of clusters selected by the hierarchical clustering algorithm is shown in figure 5A. This particular motif contains 55 occurrences, plotted as triangles based on their 1-dimensional position relative to the transcription start site. These occurrences are broken into clusters by the algorithm, denoted by blue ovals. A cone-enriched cluster just upstream of the transcription start site is shown in pink. This cluster contains 5/12 occurrences from cone-specific promoters, and only 4/84 occurrences in non-photoreceptor promoter regions.

After motifs were broken into position-dependant clusters, we used the same statistical procedure described above to select those clusters enriched in rod or cone promoters (IAMMS, step 5). Figure 5B–C plots the ratio between cell-specific and total occurrences (vertical axis) against the total number of promoters with at least one occurrence (horizontal axis). Points are colored based on the number of motifs with a given combination, in a similar manner to figure 2. The cone-enriched cluster cAGAAG shown in figure 5A is one of the motifs represented by the point marked in figure 5C. This point lies just inside the gray region representing a statistical threshold of p = 0.005 that was used to classify motifs as enriched in rod (or cone) specific promoter sequences. A motif corresponding to the known cone-specific *cis*-element ROP2 is also represented by a point in the gray region of figure 5C. Figure 5B shows the same representation as figure 5C for rod-specific motifs. A previously characterized rod-specific motif, NRE, is represented by a point that lies just inside the gray region (marked in figure 5A), indicating the biological relevance of motifs represented in this region.

**Figure 6:**
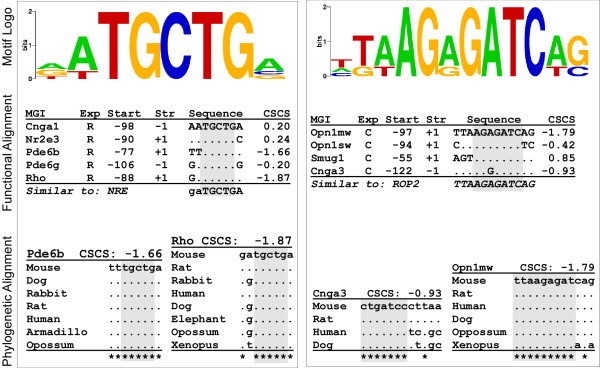
Predicted rod (left) and cone (right) enriched motifs. Notations are the same as figure 3. Cross-species alignments for *Pde6b*, *Rho *(left), *Cnga3*, and *Opn1mw *(right) occurrences are shown on the bottom. All occurrences are highly conserved across species (CSCS -1.66, -1.87, -0.93, and -1.79). The rod-specific prediction is similar to the known rod-motif NRE. The cone-specific motif contains a previously known binding site (ROP2) for which it predicts additional occurrences. Non-photoreceptor occurrences have been removed for simplicity (See additional files 1 and 2).

A detailed view of the NRE-like motif identified after step 5 is shown in the left panel of figure 6. The predicted motif contains a core region (aTGCTGa). The occurrence in the *Rho *promoter at -88 bp (occurrences are enumerated below the logo in figure 6) has already been validated experimentally [23]. Two sample cross-species phylogenetic alignments are shown below the functional alignment in figure 6 (*Pde6b *and *Rho*). In this case, these occurrences are very highly conserved relative to the surrounding sequence.

Another known transcription factor binding site detected in this study corresponds to the recently discovered cone-specific sequence ROP2, shown in the right panel of figure 6. This prediction contains an occurrence in the *Opn1mw *promoter that was recently discovered to be required for cone-specific expression [24]. Previously unknown occurrences of ROP2 were predicted in the promoter of *Opn1sw*,*Smug1*, and *Cnga3*. The newly-discovered occurrence in the *Opn1sw *promoter shows remarkable position-conservation relative to the transcription start site when compared with the known *Opn1mw *occurrence: -94 and -97 bp, respectively, a difference of only 3 bp. Selected phylogenetic alignments (figure 6, right panel, bottom) show that the occurrences in the *Cnga3 *and *Opn1mw *promoters are highly conserved through evolution. In addition to increasing confidence in predictions, the ROP2 detection also provides exciting new targets for a *cis*-element that is pertinent for cone-specificity.

The 12 highest scoring (E-value) rod- and cone-enriched position dependent predictions are shown on the bottom of figure 4. The example given in figure 5A (cAGAAG) can be found among cone-enriched motifs in row 7. Among the high scoring motifs, 6 rod and 3 cone predictions are similar to known motifs whose specific binding positions (with the exception of NRE) are not known, including four putative initiator (INR-like) elements, NRE, an IL-6 effector, an RXR binding site, ROP2, a putative TATA-like motif, and an Engrailed homeodomain binding site. Phylgoenetic conservation is relatively high for several of the elements, including two conservation scores less than -1 for cone-enriched predictions (TATA-like: -1.08 and En2: -1.35). As we show in the next section, many of these motifs are corroborated by motifs predicted by DME and/or BioProspector.

### Comparison with DME and BioProspector

To increase confidence in our predictions, we compared motifs discovered using IAMMS to those discovered using existing *de novo *motif discovery algorithms, DME and BioProspector. For both of these algorithms, a smaller section of the upstream region was employed (500 bp of upstream sequence and 100 bp of UTR) for a more similar comparison to IAMMS position clustering implementation. In order to return useful results, promoter regions needed to be repeat masked prior to analysis. Since the rod- and cone-specific sets are too small to be compared directly against each other, cone promoters were compared against the combined set of background and rod promoters to evaluate cone-enrichment. The same approach was used to identify rod-enriched predictions.

**Figure 7:**
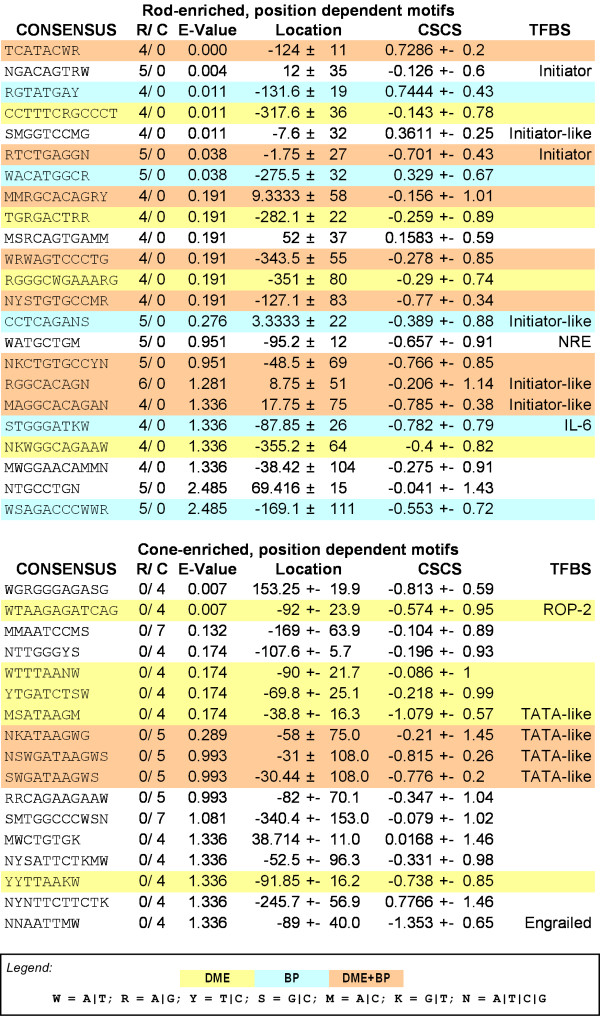
Comparison of rod (top) and cone (bottom) specific predictions made by IAMMS to those made by either DME (yellow), BioProspector (blue), or both DME and BioProspector (orange). For each prediction, the consensus sequence is given using IUPAC ambiguity codes (given in the legend). The following columns represent the ratio of rod to cone occurrences, the E-value of cell-specificity, the mean occurrence position in the promoter relative to the transcription start site, the mean cross-species conservation score, and similarity to any well-known transcription factor binding sites.

The top 10 motifs for each motif length between 6 and 10 bp (DME) or 6 and 12 bp (BioProspector) were compared with the top IAMMS predictions. This comparison is shown in Figure 7. Predictions made by IAMMS and confirmed by DME or BioProspector are highlighted in yellow (DME), blue (BioProspector), or orange (both DME and BioProspector). It is interesting to note that rod predictions for DME and BioProspector were in agreement with IAMMS a much higher proportion of the time (nearly 80%) compared to cone predictions (just under 50%). This difference between the numbers results from a much lower rate of agreement between IAMMS and BioProspector in cone sequences. Compared to BioProspector, the rate of agreement between IAMMS and DME in rods and cones is similar (47% in cones, 57% in rods). We conclude that although they use different underlying algorithms, results obtained using DME are more similar to IAMMS compared with BioProspector.

Overall, of 40 rod- and cone-specific predictions, 25 (over 60%) are confirmed by either DME or BioProspector and 11 (nearly 30%) were confirmed by both. Major predictions, including the ROP2 binding site, Initiator, TATA-like, and IL-6 (discussed in detail below) were corroborated by at least one motif discovery algorithm. The initiator-like and TATA-like predictions were identified by all 3 algorithms, increasing our confidence in these predictions.

## Discussion

In this article, we use a combination of motif discovery algorithms to identify putative *cis*-elements that may be responsible for differences in gene expression between rod and cone photoreceptors. We identified 34 conserved motifs highly enriched in either rod or cone photoreceptor genes. Our predictions can be divided into three distinct groups:

1. Completely new motifs that bare no resemblance to known transcription factor binding sites. This first group contains 20 motifs, most of which are confirmed by at least two discovery algorithms, or have a high degree of phylogenetic conservation.

2. Motifs similar to *cis*-elements with known photoreceptor function. This second group contains 5 motifs, including motifs that have been characterized by previous experimental studies (NRE, ROP2) as well as motifs whose putative binding sites are unknown (RXR, En2, and IL-6 effectors). It is notable that all these motifs were derived without using any specific *a priori *knowledge.

3. Motifs similar to known *cis*-elements whose involvement in photoreceptor function has not yet been established. This final group includes the TATA-like and Initiator-like sequences enriched in cone and rod promoters, respectively (see below for more details).

### RXR and En2 binding motifs in promoters of cone-specific genes

Previous microarray studies suggest that at least 4 transcription factors (RXRγ, En2, Sall3, and Prdm1) are more active in cone photoreceptors than rods [2]. The role of RXR is supported by additional biochemical studies which demonstrate that RXRγ plays a vital role in patterning cone photoreceptors in response to signaling by thyroid hormone receptor β2 [25,26]. The RXR prediction is shown in figure 4, position independent, row 3. Functional RXR *cis-*elements often contain a degenerate repeat of the invariant core in close proximity [27]. Therefore, we examined the promoter sequences surrounding predicted RXR sites for degenerate variations of the putative core selected by IAMMS. Out of 5 sites, 4 contain an additional occurrence of G(N [0–2])TCA within 4 bp of the recognized site (see the image in additional file 3 online). This is very unlikely to occur by chance (p~3.7 × 10^-4^), further increasing our confidence that the predicted motif binds RXR-family transcription factors.

The En2-like motif is shown in figure 4 cone-enriched position dependent row 9. The prediction includes the central portion of the optimal En2 homeodomain transcription factor consensus (TAATTA) detected by *in vitro *selection experiments [28,29]. While a corresponding motif was detected only by IAMMS (figure 7, last cone-enriched row), occurrences of the Engrailed-like prediction are highly conserved through evolution (-1.35) suggesting that the motif is functional. A similar prediction (cone position dependent, row 10) contains many of the same occurrences, but shifts the core by ~2 bp and adds an additional A to the 3' end. Like the first prediction, it is also highly conserved through evolution, centered in the same region, and cone-specific. Moreover, this second prediction was also detected by DME (figure 7, cone-enriched, 3^rd ^row from bottom). If validated experimentally, occurrences of this AATT motif will be the first reported promoter binding sites for En2.

We were unable to find binding sites for Sall3 or Prdm1 in the experimental literature. Some of the unidentified motifs predicted in this study (group 1) may correspond to binding sties for these transcription factors. Future experimental studies will be required to discover any correspondence between these transcription factors and motifs predicted in this study.

### IL-6 Binding Motif in Promoters of Rod-Specific Genes

One of the rod-specific predictions, detected by both IAMMS and BioProspector, is similar to an IL-6 effector (figure 7, row marked IL-6). This is interesting in the context of recent findings that in rodents, signaling by IL-6 family members CNTF and LIF can block the formation of rod photoeceptors during development [30,31]. According to the literature, peak IL-6 effector activity is obtained by the invariant core (CTGGGAA) and another degenerate occurrence (CTGGAA) appearing nearby [32]. Our prediction corresponds to the first invariant core (CTGGGA). To determine if a degenerate occurrence appeared nearby, we took rod-enriched IL-6-like predictions andsearched nearby promoter sequence to find if any elements similar to the core were present. We found that 4 rod-specific promoter sequences, including *Pde6b*, *Gnat1*, *Pde6d*, and *Rho *contain an exact copy of either the degenerate sequence or the high-affinity core binding sequence within 50 bp of a predicted occurrence (see the image in additional file 4, as well as additional files 1 and 2 for more IL-6 like predictions). It is interesting that in chick, where artificial IL-6 stimulation increases the number of rod photoreceptors [33], only one orthologous promoter (of all those in table 1) contains both the invariant core and a degenerate consensus within 50 bp of one another. The correspondence between empirical evidence and occurrences of IL-6-like motifs lends support for the biological relevance of the IL-6 prediction.

The high-affinity core and a degenerate occurrence missing only the final A (i.e. CTGGA, also within 50 bp) was found in the *Nrl *promoter. This predicted site is likely to be significant considering the important role *Nrl *plays in rod photoreceptor differentiation [2]. This observation suggests that one possible mechanism for IL-6 regulation of rod-differentiation involves suppression of the *Nrl *gene product.

### Differences in the Core Promoter of Rod and Cone-Specific Genes

One of the most striking findings of this study is differences in the core promoter region of rod-and cone-specific genes. We predicted several rod-enriched motifs centered on the transcription start site that are similar to characterized initiator consensus sequences, but no enrichment of degenerate initiator-like sequences were found specific to cones. Conversely, a TATA-like motif was detected in almost all cone promoters near the appropriate position upstream of the transcription start site, whereas it was absent from rod promoters.

**Figure 8:**
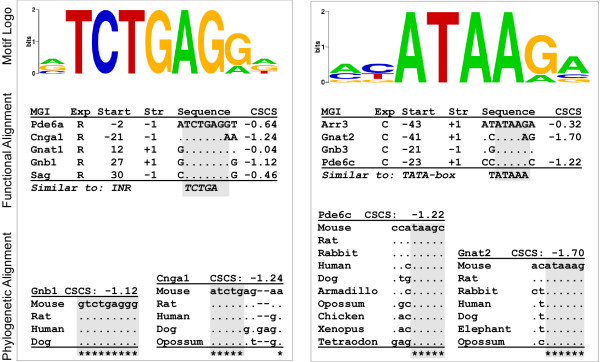
Predicted rod (left) and cone (right) enriched motifs in the same format as figure 6. The rod motif is similar in sequence and mean position to the central portion of an initiator element, and the cone to a TATA box.

Figure 4 depicts 4 unique, rod-enriched motifs whose mean position lies near the transcription start with relatively low position variance. Three of these motifs are similar to portions of experimentally-validated initiators (INR-like), including the motifs in row 4 (aGGTCC) [34], row 6 (TCTGAG) [35], and row 11 (GCACAG) [36]. The fourth motif (ACAGTGa), in row 2, is also attributed to the initiator-like group because its antisense mismatches the accepted initiator consensus (YYANWYY) at only one position. More details of the TCTGAG motif are shown in the left panel of figure 8. We detected occurrences of this motif near the transcription start site in 5 rod promoters. The *Pde6a*, *Cnga1*, and *Sag *occurrences are on the -1 strand, and are consequently highly similar to the pyridine rich initiator consensus (YYANWYY). The proximity of the 4 motifs to the annotated transcription start site, their phylogenetic conservation, as well as similarity to portions of experimentally characterized initiators suggests that these motifs may function as degenerate initiator sequences in rod-specific promoters.

In cone promoters we found a different core promoter element, ATAA, a motif similar to the central portion of a TATA box (see Figure 4 and Figure 7). One such prediction is depicted in the right panel of figure 8. Occurrences of this particular motif are found in 4 cone promoters, *Arr3*,*Gnat2*,*Gnb3*, and *Pde6c *(figure 8, right panel). These occurrences are located between 20 and 45 bp upstream of the transcription start site, close to the typical position of a TATA-box [37], supporting the idea that it is, indeed, a degenerate variation on the TATA consensus. A high degree of phylogenetic conservation of this motif and corroboration by both DME and BioProspector further support the biological relevance of this prediction.

Many of the ATAA occurrences contain an additional T on the beginning of the motif, making them even closer to the classic TATA-consensus. We conducted a search for TATA-like sequences in the core promoter of rod and cone genes. It is interesting that the sequence TATAA (or its antisense) appears in 7 cone promoters (*Opn1mw*, *Opn1sw*, *Cngb3*, *Arr3*, *Pde6c*, *Smug1*, and *Cnga3*) between -180 to +60 relative to the transcription start site. Conversely, this sequence is only found in two (*Rho *and *Cnga1*) out of 11 rod promoters. The enrichment of the TATAA sequence in cones, although not as pronounced as ATAA, lends further support to the idea that the cone promoters studied here are initiated by a TATA box. It is notable that except for *Elovl2 *and *Pde6h*, an occurrence of either sATAAgw or TATAA is present near the transcription start site in all cone-specific promoters.

Experimental evidence supports the biological relevance of the ATAA prediction, regardless of whether it is, indeed, a degenerate TATA-box. A recent experimental study deleted two occurrences of TATA-like motifs from the *Arr3 *promoter [38] and observed that the previously cone-specific promoter drove transgene expression in rods as well. In light of our predictions, we suggest that a TATA or TATA-like motif in the core promoter plays a central role in the differences between rod and cone expression patterns.

### Limitations

The fact that the number of genes specifically expressed in either rod or cone photoreceptors is rather small makes the application of *de novo *motif discovery approaches that heavily rely on statistical analysis difficult. Because of this consideration, we took two independent approaches designed to increase the accuracy of our results. First, we employed a large number of non-photoreceptor genes as a negative control, and evaluated enrichment of motifs in either rod or cone promoters relative to this large dataset. Second, we applied 3 motif discovery software packages that use different algorithms to identify motifs. While we do not filter motifs that are identified by only one algorithm from our final database, we do provide a table of overlaps (figure 7) as additional information that can be used to evaluate predictions. Together, these two approaches should minimize both false positive and false negative errors.

In the present study, we selected promoters based on ENSEMBL annotated transcription start sites. However, recent reports suggest that two separate ambiguities exist in transcription start site annotations. One, the so-called "borad" class of transcription start sites, represents inherent local variation over 50–100 bp [37,39]. This complication should not have a major impact on the quality of our predictions. Since hierarchical clustering automatically chooses the mode and variance of a motifs' position distribution relative to the transcription start site separately for each motif, IAMMS should turn up the same predictions with some additional position variance.

A second ambiguity is the recent observation that a majority of genes are driven by two or more alternative promoter sequences [37,39]. To determine the relevance of this finding for our study, we searched the database of transcription start sites (DBTSS) for genes used in this study, and found only 3 genes (*Nr2e3*, *Gngt2*, and *Pde6h*) that contain potential alternate transcription start sites far from the ENSEMBL annotation, and 4 additional genes (*Pde6d*, *Pde6b*, *Gnb3*, and *Elovl2*) that contain alternate transcription start sites within 200 bp [40]. However, in all of these cases the alternate transcripts were identified in non-retinal tissue, and therefore the alternate start sites do not pertain to our present application. In addition, 8/23 promoters that we selected for analysis are validated experimentally (*Arr3 *[38,41], *Pde6c*[42], *Opn1mw *[24,43], *Pde6a*[44], *Gnat2*[45], *Sag *[46,47], *Rho*[23], and *Nrl*[48]). Those considerations expressed above give us confidence in the promoter regions selected for this study.

This study did not detect motifs corresponding to two transcription factors known to be enriched in rod-photoreceptors compared to cones, including Mef2c [22] and NR2E3 [49]. One of the causes of this omission could potentially be the multiple severe constraints in our selection criteria that were introduced to maximally reduce the rate of false positive predictions. In the case of NR2E3, another potential reason may be that NR2E3 may not bind DNA directly *in vivo*. Rather, recent findings suggest that NR2E3 regulates expression indirectly by interactions with CRX [50]. If there is no NR2E3 binding directly to DNA, it is not surprising that we do not identify a motif for this transcription factor.

## Conclusion

Using a panel of three motif discovery algorithms (IAMMS, DME, and BioProspector), we predict 34 putative *cis*-elements that may be vital for maintaining either rod or cone gene expression patterns. Our predictions include many previously unknown motifs, known *cis*-elements involved in maintaining the differences between rod and cone expression patterns, as well as binding sites for transcription factors with no known photoreceptor function. Our most important predictions include specific sites for RXR and Engrailed family members (enriched in cone promoters) and IL-6 effectors (enriched in rod promoters). We predict differences in the core promoter between rod and cone phototransduction genes. While rod promoters are enriched in putative initiator-like motifs and are TATA-less, cone promoters are enriched in TATA-like motifs. To simplify access to our findings, we provide an on-line database containing detailed information about the exact position of the motifs with the respect to the transcription start and their phylogenetic conservation (additional files 1, 2).

## Methods

### Building photoreceptor-specific list

Genes in the photoreceptor-specific list (table 1) were selected as follows. Cone genes (except *Cnga3*) were selected using microarray data from NRL or NR2E3-knockout mouse retina that are known to produce a rod-deficient phenotype [2,15]. We included *Cnga3 *which was found to be cone-specific by experimental studies [21]. Rod genes *Sag *[17], *Pde6d *[20], *Nrl *[18], *Nr2e3 *[19], and *Gnb1 *[15] were previously observed to be expressed in rod but not cone photoreceptors by biochemical studies [16-21]. The remaining rod genes (*Rho*, *Pde6a*, *Pde6g*, *Pde6b*, *Gnat1*, and *Cnga1*) were selected based on microarray data comparing FACS sorted rods to a model of cone-photoreceptors [22]. The latter involves FACS sorting cells expressing GFP by the NRL promoter in NRL knockout mice, and is demonstrated to be a good model for cone photoreceptors [51]. To pick the rod genes we obtained, raw CEL files for 4 normal and 4 NRL knockout animals were obtained using the Gene Expression Omnibus website. The data were MAS5 normalized and averaged using the bioconductor package [52]. We selected genes involved in the phototransduction pathway that were significantly down-regulated in the *Nrl *knockout samples (p < 0.02; Student's t-test).

For each gene in table 1, 2 kb of sequence upstream of the annotated transcription start site and the entire 5' UTR of the mouse was obtained from ENSEMBL (Mouse v.36, Aug. 2005). Two genes (*Nrl *and *Gngt2*) contained two annotated transcription start sites within 1000 bp of each other, and each promoter contained a UTR. In both cases, the promoter closer to the translation start site was chosen. This choice effectively included the region immediately upstream of each transcription start site; for *Nrl*, this choice corresponded to an experimental study [48].

### Selecting background promoter set

We constructed a background sequence set from genes that are not expressed in either rods or cones, but are expressed in most tissues, in a tissue independent manner. To construct this background set, we first identified all genes that are not expressed in adult rod or cone photoreceptors. To do this, we used the MAS5 normalized FACS sorted microarray data obtained in the previous section. From this data, we obtained a list of REFSEQ IDs where all probes associated with each REFSEQ ID was marked absent by the Affymetrix perfect match/mismatch designation.

To evaluate tissue specificity, microarray data from the mouse gene-atlas [53] was obtained from NCBI's Gene Expression Omnibus (GSE1133) using R's Bioconductor plugin [52] and GEOquery [54] packages. Average expression of each gene in each tissue was calculated, and probe sets were converted to REFSEQ IDs. Next, we calculated the ratio of maximum expression to the sum of expression in all tissues. This tissue-specificity ratio ranged between 0.02 (nearly equal expression between all tissues) and 0.98 (highly specific to one tissue). For the background set, we selected all REFSEQ IDs for genes with a ratio less than 0.03 that are also absent from both adult rod and cone photoreceptors (n= 84). For all of these genes, 2 kb of upstream sequence and the entire 5' UTR was obtained using ENSEMBL's BioMart.

### IAMMS procedure

The flowchart of IAMMS is depicted in figure 1. The input for the algorithm consists of 3 sets of promoter sequences, including 11 rod-specific sequences, 12 cone-specific sequences, and an additional set of background sequences that do not drive expression in photoreceptors. All promoters were passed through an iterative alignment procedure (step 1) that returns a motif for each sequence ≥ 8 bp in length that appears more than once in photoreceptor promoters. The resulting database of potential motifs was scanned for sequences similar to a known simple or interspersed repeat sequence (step 2). Motifs were evaluated for cell-specificity using a binomial model of enrichment (statistical annotation, step 3) to create predictions for cell-specific motifs. Ambiguous motifs were filtered to extract sets of position-enriched occurrences using an agglomerative clustering procedure (step 4). Position-enriched clusters were subsequently analyzed using statistical annotation (step 5) to create a set of position-specific predictions. Both position-enriched and non-enriched predictions were subsequently analyzed by phylogenetic analysis (step 6) and were compared to known *cis*-elements (step 7).

### Step 1: Iterative alignment

All sequences ≥ 8 bp in length that appear more than once in rod and cone photoreceptor promoters were identified using the BLAST implementation distributed by Washington University [55]. We used scoring parameters that were observed to return short, nearly exact matches: +2 for a match, -3 for a mismatch, and a threshold bit score of 16. Gaps were allowed, but using the default score of -20 gaps rarely appeared (impossible in any sequence pair less than 28 bp match of flanking surrounding a gap). After completion, we separated pairwise alignments into a database of individual sequences. We filtered this database, so that each sequence occurs exactly once. This database contains each sequence ≥ 8 bp in length that occurs at least twice in the photoreceptor promoter set. Next, we constructed a multiple alignment for each sequence returned in the pairwise alignment. In the second iteration, we scanned all promoter sequences (rod, cone, and 84 background promoters) using each sequence identified in the previous database. BLAST was run using the same match and mismatch parameters as the first iteration, but the threshold bit score was changed for each sequence. To calculate the threshold bit score, we multiplied the sequence length by 1.4 (we also tried a variety of constants between 1.3 and 1.7). The output of this step is a series of multiple alignments – one alignment for each sequence occurring twice in photoreceptor promoters.

### Step 2: Masking Longer Sequences to RepBase

Longer sequences were evaluated to examine potential similarities to known repeats. BLAST was used to compare each sequence ≥ 20 bp in length to all mouse repeats represented in RepBase [56] v.11.07. A scoring scheme of +2 (match), -3 (mismatch), and 20 (threshold) was used.

### Step 3: Statistical Annotation & Bonferroni Correction

Let *k*_*r *_and *k*_*c *_be the number of rod- and cone-specific promoters that contain at least one occurrence in a motif with *n *occurrences. We take the p-value of cell-specific enrichment to be the binomial probability of selecting a list that contains *n *sequences, of which *k*_*r *_or *k*_*c *_are mapped to rod- or cone-specific promoters. The probability of selecting one rod/cone-specific promoter is the number of rod or cone promoters divided by the total number of promoters (11/107 = 0.103 for rods).

Due to overlap between different motif core regions, we observed underdispersion relative to the binomial model described above. We corrected the p-value by Z-score normalizing across all groups with a given number of occurrences using an empirically derived mean and standard deviation. To perform the Bonferroni correction, we multiplied the corrected p-value by the total number of sequences considered for cell-specific expression, not counting motifs with less than 4 occurrences in photoreceptor promoters or similar to repeat sequences longer than 20 bp (38,779). For the sake of simplicity our calculations do not take into account dependence between motifs with highly similar core regions, and are therefore highly conservative. This Bonferroni corrected expected false positive rate is referred to as the E-value. We select all motifs with an enrichment E-value less than 2.5 in one photoreceptor cell type, and also require that no occurrences are found in the alternate photoreceptor cell type.

### Step 4: Agglomerative Hierarchical Clustering

Single link hierarchical agglomerative clustering was performed on the distance to the transcription start site for each motif. Motifs are broken into the minimum number of clusters when the mean inter-cluster variance reaches 1% of the total variance. To ensure that the data contains an underlying structure that can be described by clusters, only motifs with an agglomerative coefficient greater than or equal to 0.7 were analyzed using agglomerative clustering. All computations were performed using the R-cluster package [57] (v.2.3.0). Samples of clusters selected by this procedure are shown in figure 5A. In addition to single link, Euclidean and Ward's algorithms in the R-cluster package were tested, though all results are reported on single-link results.

### Steps 5: Statistical Annotation

Each position enriched cluster with occurrences in the first 400 bp upstream of the transcription start site was analyzed for rod/cone specificity using the statistical annotation procedure described in step 3.

### Step 6: Phylogenetic Analysis

To compare the conservation of a putative mouse *cis*-element to other predictions, we applied a recently described model [58] involving a comparison between the actual number of substitutions in the predicted *cis*-element to the expected rate of conservation between all species in the alignment. First, alignments corresponding to promoters of interest were extracted from existing whole-genome alignments using the UCSC genome browser [59] (mm8 version). Raw alignments corresponding to each mouse promoter were obtained as different regions known as alignment blocks. We defined the cross-species conservation score (CSCS) as the Z-score of the calculated substitutions in our sequence of interest, relative to all surrounding windows (the same size as our comparison sequence) in the same local alignment block. To calculate the mean and standard deviation, we used a sliding window (the same size as the prediction) locally, in the alignment block. Negative results mean less than the average number of mutations are found in the window (i.e. the sequence is conserved); positive values mean that there are more differences. Sequences corresponding to gaps where no alignment is available between the mouse sequence and other species were not included in the analysis. References to these scores were left blank in the figures and additional data files.

### Step 7: Comparison to known motifs

Rod/cone-specific motifs were compared to a database of known motif and consensus sequences using the Transcriptional Element Search System [60] (TESS). Searches for each list were performed on the sequence returned in the first BLAST iteration using the TESS combined search option. Log-likelihood score filtering was used for both string and weight matrix functions. The minimum log-likelihood ratio was set to 12 for string scoring and 10 for weight matrix scoring. Matches were visually inspected, and deemed to be similar only if nearly identical in the invariant core region. TESS missed several regulatory sequences available in the experimental literature, many of which have special relevance to a photoreceptor system. These similarities were annotated by hand and are included in figures 4 and 7.

### Comparison between predictions

Predictions highly similar in the core region, but differing in ambiguous peripheral positions, (for example, see figure 7, rows 8–10 and row 7 antisense) were grouped prior to the creation of figure 4 and counting predictions for the text. Sequences were grouped according to the methods described in [14]. A single vector was created by concatenating column vectors from the position-weight-matrix representation of a motif. The maximum Pearson coefficient over each possible alignment between two separate motifs (including sense-antisense) was subsequently calculated. When comparing motifs with a different length, the smaller motif was compared against the larger to emphasize similarities in the invariant core region. During this step, overhangs were filled in with P(A) = P(T) = P(C) = P(G) = 0.25. Motifs were considered highly-similar if the Pearson coefficient was greater than 0.85.

### DME/BioProsprector procedure

The same promoters used for IAMMS were repeat masked [61] using the following settings: wublast algorithm, DNA source set to mouse, and default sensitivity. After masking, sequences were chopped to include only 500 bp of upstream sequence and 100 bp of UTR, when available. Each software program was run with default settings, except that the number of motifs to return was set to 10, and the motif size was varied between 6 and 10 (for DME), or 6 and 12 (for BioProspector). When identifying cone-specific motifs, cone promoters were compared against the combined set of rod-specific and background promoters (and vice-versa). Results for each run were added into the same table and sorted by the score given by each program. Output motifs were compared to predictions made by IAMMS using the method described in [14], and repeated above (see *Comparison between predictions *in Methods section).

## List of abbreviations

CSCS: Cross Species Conservation Score

DBTSS: Database of Transcription Start Sites

FACS: Fluorescent Activated Cell Sorting

IAMMS: Iterative Alignment/Modular Motif Selection

RXR: Retnoid X Receptor

TESS: Transcriptional Element Search System

## Authors' contributions

CGD assisted in the design and implementation of IAMMS, applied IAMMS to photoreceptor promoter regions, conducted analysis of results based on experimental work, conducted microarray analysis, and wrote the paper. VAM constructed the initial photoreceptor-specific list and assisted in analysis of the results. MQ assisted in the implementation of IAMMS and some web-based tools to distribute predictions. BEK assisted in the construction of the photoreceptor-specific list and in the analysis of the results. AMP assisted in the design of IAMMS, contributed enormously to writing the manuscript, provided thoughtful discussion. All authors read and approved the final manuscript.

## Supplementary Material

Additional file 1 **Explanation of Supplementary Data**. Detailed information on reading HTML formatted supplementary data. Click here for file

Additional file 2 **Extended table of information on predictions**. Contains cross-promoter alignments and phylogenetic alignments for each prediction, as well as the entire list of ENSEMBL IDs for genes used in the study. Please refer to "Data Supplement Instructions.doc" for detailed information. Click here for file

Additional file 3 Additional figure 1. Region surrounding predicted IL-6 sites in 5 rod promoters. Sequences identified by IAMMS are shaded in gray; copies of the core (including the degenerate copy CTGGA) are outlined in black. Click here for file

Additional file 4 Additional figure 2. The location of predicted RXR core binding sites (gray) and the adjacent degenerate core region (outline) in 4 cone promoters. Click here for file
