# Supplementary material for: Bioinformatic identification of novel putative photoreceptor specific cis-elements
Source: BMC Bioinformatics. 2007 Oct 22;8:407. doi: 10.1186/1471-2105-8-407 (PMC2225425; doi:10.1186/1471-2105-8-407)
Supplement: Additional file 1 — Explanation of Supplementary Data. Detailed information on reading HTML formatted supplementary data. [file 1471-2105-8-407-S1.ZIP › c.NS.html]

cis-Browser 

Predictions via cis-Browser

|  |
| --- |
| - ID: Opn1sw\_529\_539     R|C/ N: (8/18)     Z: 3.9027555    Consensus:                           YNAGGGTTCMS   - Opn1sw              -1472  -1461  +  TTAGGGTTCAC     - Mouse                           gtgaaccctaa Rat                             gtgaaccctaa                                 \*\*\*\*\*\*\*\*\*\*\*   CSCS: -1.5825941781491326   - Arr3                -1213  -1202  -  TTAGGGTTCAC     - Mouse                           gtgaaccctaa Rat                             gtgaactctaa Human                           ttgagccctcc Dog                             ------cctcc                                        \*\*     CSCS: 0.46421003318492565   - Opn1sw               -625   -614  -  TCTGGGTTCAC     - Mouse                           tctgggttcac Rat                             accaggttcac                                  \*  \*\*\*\*\*\*\*   CSCS: 0.38315062168492825   - Opn1mw              -1783  -1772  +  TCAGGGTTCAC     - Mouse                           tcagggttcac Rat                             tcagggttcat                                 \*\*\*\*\*\*\*\*\*\*    CSCS: -0.32127300435723505   - ENSMUSG00000039713  -1415  -1404  +  TTAGGGTTCCC   - Gnat2               -1978  -1967  +  TTAGGGTTCTT     - Mouse                           tt-agggttc-------tt Rat                             ttaagggttc-------tt Human                           tt-cgacctcag--tagtt Dog                             tt-cgaactcaagttaaat                                 \*\*  \*   \*\*        \*   CSCS: -1.1706574178218314   - ENSMUSG00000032059  -1080  -1069  -  TTAGGGTTCTC   - Gngt2               -1201  -1190  -  TTAGAGTTCAC     - Mouse                           g Rat                             - Mouse                           gaactctaa Rat                             --------- CSCS: 1.2571331213495045   - ENSMUSG00000024981    -45    -34  -  TCCGGGTTCAC   - Smug1               -1226  -1215  +  GGCGGGTTCAC     - Mouse                           gtgaacccgcc Rat                             gtgaacccgcc Human                           atgaacctgcc                                  \*\*\*\*\*\* \*\*\*   CSCS: -0.46938343076222844   - ENSMUSG00000024571  -1066  -1055  -  CCAGGGTTCAA   - ENSMUSG00000000738  -1394  -1383  +  CGGGGGTTCAC   - ENSMUSG00000000738    126    137  -  TGCGGGTTCAC   - ENSMUSG00000068264   -657   -646  +  AGAGGGTTCAG   - ENSMUSG00000013150  -1146  -1135  -  CCAGGGTTCAG   - ENSMUSG00000043760    169    180  -  TTAGGGTTGAT   - Pde6c                -273   -262  -  TTAGGGTTAGC     - Mouse                           gctaaccctaa Rat                             aaaaaccctaa Human                           aaaaaatccaa Dog                             g-------cag                                          \*    CSCS: 1.3875062086165126   - Pde6h               -1228  -1217  -  AGAGGGTTCAA     - Mouse                           ttgaaccctct Rat                             ctaaacacttt Human                           ccaaacacttt Dog                             ccaaacactct                                    \*\*\* \*\* \*   CSCS: 0.3040209737924258   - ENSMUSG00000026799   -957   -946  -  TTAGGGTTTTT   - ENSMUSG00000034278  -1570  -1559  +  CTAGGGTTCTG   - ENSMUSG00000034278  -1858  -1847  -  CAAGGGTTCAG   - ID: cnga3\_2138\_2150     R|C/ N: (5/7)     Z: 3.8463635    Consensus:                           NYCTGATGCTGTK   - cnga3                 137    150  +  CTCTGATGCTGTT     - Mouse                           ctc---tgatgctgtt Rat                             ttc---caatgctgtt Human                           actgagcggcgcagct Dog                             actttatgctgtttca                                           \*        CSCS: -0.26533234066205275   - Gnat2               -1068  -1055  -  CTCTGATGCAGTT     - Mouse                           aactgcatcagag Rat                             aactgtattagaa                                 \*\*\*\*\* \*\* \*\*\*    CSCS: 1.5651681717893955   - Elovl2               -335   -322  +  GCCTGATGCTGTG     - Mouse                           cacagcatcaggc Rat                             ---------aggc Human                           ---------gagc                                            \*\*   CSCS: 2.5850221778933724   - ENSMUSG00000024787   -518   -505  -  TTCTGATGCTGAA   - ENSMUSG00000043760    136    149  -  GCCTGATGCTGTG   - Gnb3                -1965  -1952  +  GCCTGATGCTGTG     - Mouse                           cacagc---- Rat                             ---------- Human                           cacaga---- Dog                             ccctga---- Chicken                         ----------                                       \*\*\*\*\*\*      \*\*\*                 \*\*\*\*\*\*\*\*\*\*\*\*\*\*\*\*\*\*\*\*\*\*\*\*\*\*\*\*\*\*\*              \*\*        CSCS: 0.612674442688431   - Arr3                 -240   -227  -  TTCTGATGATGTT     - Mouse                           aacatcatcagaa Rat                             aacatcatcagaa Human                           aacatcatcagaa Dog                             aacactatcagat Opossum                         ----------gag                                           \*\*    CSCS: -1.527876023993614   - ID: Gnb3\_1797\_1809     R|C/ N: (5/7)     Z: 3.8463635    Consensus:                           NACAGGGTCATMK   - Gnb3                 -204   -191  +  AACAGGGTCATCT     - Mouse                           agatgac--------cctgtt Rat                             agatgac--------cctgtt Human                           agatggc--------cccatt Dog                             aggtggc--------cc-att Opossum                         agatgatggcaaacatccttc                                 \*\* \*\*           \*  \*    CSCS: -0.5577113005350727   - ENSMUSG00000029415  -1855  -1842  -  AACATGGTCATCT   - ENSMUSG00000037060  -1126  -1113  +  AACAGGGTCAAAG   - cngb3                  37     50  +  TACAGGGTCATAT     - Mouse                           tacagggtcatat Rat                             tacagggtcatag Human                           caca--gtcataa Dog                             -acagagttgtaa Opossum                         caca--ggcagaa Chicken                         cacg--gtaaaag                                  \*\*   \*    \*    CSCS: 0.9021743976791854   - Pde6c               -1847  -1834  +  CACAGGGTCATGG     - Mouse                           cacagggtcatgg Rat                             cacagagtcatga                                 \*\*\*\*\* \*\*\*\*\*\*    CSCS: -0.191482117569823   - Gngt2                -770   -757  -  GGTAGGGTCATCT     - Mouse                           ag----------atgaccctacc Rat                             ag----------atgaccctgcc Human                           aggatgggtcccagggccccact                                 \*\*          \* \* \*\*\*  \*    CSCS: -0.504303059896423   - Pde6h                -197   -184  -  GACAGGGGCATCT     - Mouse                           agatgcccctgtc Rat                             agatgcccttgtc Human                           atgaacccaagtc Dog                             gagagcccaagtc                                      \*\*\*  \*\*\*   CSCS: -0.2155677347036258   - ID: Pde6d\_2007\_2017\_14     R|C/ N: (4/4)     Z: 5.0822554    Consensus:                           WGRGGGAGASG   - cnga3                 128    139  -  AGAGGGAGATG     - Mouse                           catct---------------ccctct Rat                             tgtct---------------cccttt Human                           aatct---------------ccctac Dog                             aacccctcaattcacaactgccctac                                    \*                \*\*\*\*     CSCS: -0.9331392177202974   - Gngt2                 168    179  -  AGAGGGAGACC     - Mouse                           acaggaaggcc Rat                             acaggaaggcc Human                           acaggaaggac Dog                             ataggaaggaa                                 \* \*\*\*\*\*\*\*     CSCS: -1.4801072634185806   - Smug1                 177    188  -  TCTGGGAGAGG   - Gnb3                  140    151  -  TGGGGGAGAGG     - Mouse                           tgggg----- Rat                             taggg----- Human                           tgggg----- Dog                             tgtgg----- Opossum                         taagg-----                                 \*  \*\*\*\*\*\*\*\*\*\*\*\*\*\*\*\*    \*\*\*\*\*\*\*\*\*\*\*\*\*\*    CSCS: -0.028567612961372283   - ID: Opn1mw\_1904\_1915\_1     R|C/ N: (4/4)     Z: 5.0822554    Consensus:                           WTAAGAGATCAG   - Opn1mw                -97    -85  +  TTAAGAGATCAG     - Mouse                           ttaagagatcag Rat                             ttaagagatcag Human                           ttaagagatcag Dog                             ttaagagatcag Opossum                         ttaagagatcag X.tropicalis                    ttaagagataaa                                 \*\*\*\*\*\*\*\*\* \*    CSCS: -1.7901212098465105   - cnga3                -122   -110  -  TTAAGGGATCAG     - Mouse                           ctgatcccttaa Rat                             ctgatcccttaa Human                           ctgatcctctgc Dog                             ctgatcccttgc                                 \*\*\*\*\*\*\*  \*     CSCS: -0.9309443787631781   - Opn1sw                -94    -82  +  CTAAGAGATCTC     - Mouse                           gagatctcttag Rat                             gagatctcttag Human                           gaaatccctaaa Dog                             gagctgtccaag Opossum                         gggacccttgag                                 \*         \*    CSCS: -0.42937732572149695   - Smug1                 -55    -43  +  AGTAGAGATCAG     - Mouse                           ctgatctctact Rat                             cttatc--tact Human                           ctaaccgttagt                                 \*\* \* \*  \*\* \*   CSCS: 0.8540536158360592   - ID: Gngt2\_844\_852\_6     R|C/ N: (7/15)     Z: 4.500767    Consensus:                           MMAATCCMS   - ENSMUSG00000036636   -123   -114  -  AAAATCCCT   - Gngt2                -191   -182  -  AAAATCCAC     - Mouse                           gtggatttt Rat                             gtggatttt Human                           gtggatttt Dog                             gtggatttt Opossum                         atggttttt                                  \*\*\* \*\*\*\*   CSCS: -1.2734106060818053   - ENSMUSG00000040213   -263   -254  +  CGAATCCAC   - ENSMUSG00000026983    -99    -90  -  AAAATCCAG   - Smug1                   0      9  -  ATAATCCAC     - Mouse                           at---aatccac Rat                             at---aatccac Human                           gt---cctccat                                  \*\*\*\*  \*\*\*\*    CSCS: 0.02466961309927132   - ENSMUSG00000044811    -37    -28  +  TCAATCCAC   - cnga3                 -18     -9  +  CCAATCCAC     - Mouse                           ccaatcca Rat                             ccagccca Human                           ccctgggg Dog                             ccctagga                                 \*\*                                         Mouse                                 Rat                                 Human                                 Dog CSCS: NaN   - cnga3                -112   -103  +  AAAATCCGC     - Mouse                           aaaatccgc Rat                             aaaatccgc Human                           gcaatcccc Dog                             gcaatcccc                                   \*\*\*\*\* \*   CSCS: -0.3845013222614813   - Pde6c                 -45    -36  +  TTAATCCAC     - Mouse                           ttaatccac Rat                             ttaatccac Human                           ttaatcctg Dog                             ttaatcctg Opossum                         taaagtcac Chicken                         ttaattagc X.tropicalis                    ttaattagc                                    \*        CSCS: -0.5210093513807389   - Pde6c                 -71    -62  -  AAAATCCTC     - Mouse                           ga--ggatttt Rat                             ta-aggatttt Human                           ca-gggattta Dog                             ta-gggatttc Opossum                         cacagggctta Chicken                         ta-ggggttta X.tropicalis                    ca-ttggttta                                  \*       \*    CSCS: -0.5210093513807389   - cngb3                 -38    -29  +  AAAATCCTG     - Mouse                           aaaatcctg Rat                             aaaatcctg Human                           aaaatcctg Dog                             aaaatcccg Opossum                         aaaatcctg Chicken                         taaa-cctg                                  \*\*\* \*\* \*   CSCS: -1.23590193995445   - Pde6h                  12     21  -  AAAATCCCC     - Mouse                           gggg---atttt Rat                             tg---aattttt Human                           tgagtacttctg Dog                             tgagtatttctc                                  \*      \* \*    CSCS: 1.0907125517312193   - ENSMUSG00000027536   -249   -240  -  CAAATCCAT   - Opn1mw               -160   -151  -  CCAATCCAC     - Mouse                           gtggattgg Rat                             gtgggttgg Human                           gcgggctcg Dog                             --------- Opossum                         gc---ttca CSCS: 1.4098661763835578   - ENSMUSG00000068391    -87    -78  -  AAAATCCCT   - ENSMUSG00000038496   -223   -214  +  GAAATCCAA   - ENSMUSG00000022414   -233   -224  +  AAAATCCGG   - ID: Pde6a\_344\_351\_17     R|C/ N: (4/5)     Z: 4.442663    Consensus:                           NTTGGGYS   - cngb3                -118   -110  -  CTTGGGTG     - Mouse                           cacccaag Rat                             cacccaag Human                           cacccaag Dog                             cacccaag Opossum                         tataaaag                                  \*   \*\*\*   CSCS: -0.9178229046171643   - ENSMUSG00000043760   -101    -93  +  TTTGGGTC   - Gngt2                -108   -100  +  ATTGGGGA     - Mouse                           attgggga Rat                             attgggaa Human                           acagggga Dog                             cctgggga Opossum                         gctagtga                                     \*  \*   CSCS: 0.15158376221343844   - Opn1mw               -104    -96  -  ATTGGGCC     - Mouse                           ggcccaat Rat                             ggcccaat Human                           ggcccaat Dog                             ggcccaat Opossum                         ggctcaat X.tropicalis                    atgccaat                                     \*\*\*\*   CSCS: -1.1918177769192795   - Arr3                 -107    -99  -  GGTGGGTG     - Mouse                           cacccacc Rat                             cacccacc Human                           -ccccccc Dog                             ---gcccc Opossum                         cctccact                                     \* \*    CSCS: 1.1703514203608933   - ID: Nrl\_735\_742\_11     R|C/ N: (4/5)     Z: 4.442663    Consensus:                           WTTTAANW   - ENSMUSG00000024906   -110   -102  +  TTTTAACA   - cnga3                -116   -108  +  CCTTAAAA     - Mouse                           ccttaaaa Rat                             ccttaaaa Human                           ctctgcaa Dog                             ccttgcaa                                 \*  \*  \*\*   CSCS: -0.17150363449065706   - cngb3                 -93    -85  +  TTTTAAGT     - Mouse                           tttt Rat                             tttt Human                           tttt Dog                             tttt Opossum                         ctta                                  \*\*    Mouse                           agt Rat                             agc Human                           agc Dog                             agc Opossum                         aat Chicken                         agc                                 \*     CSCS: 0.5051564927011539   - Gnat2                 -72    -64  +  TTTTAATT     - Mouse                           t-tttaatt Rat                             t-tttaatt Human                           t-cttaatt Dog                             t-cttaatt Opossum                         tccttaatt                                 \*  \*\*\*\*\*\*   CSCS: -1.4915936110776333   - Pde6c                 -59    -51  +  ATTTAAAG     - Mouse                           atttaaag Rat                             acttaaag Human                           tcctgaag Dog                             tcctgaag Opossum                         tcctgaac Chicken                         tcctga-c X.tropicalis                    tcctga-c CSCS: 1.4350716739642804   - ID: Pde6g\_1482\_1490\_10     R|C/ N: (4/5)     Z: 4.442663    Consensus:                           YTGATCTSW   - Gnat2                 -61    -52  +  TTGATCTGT     - Mouse                           ttgat-ctgt Rat                             ttgct-ctgt Human                           ttgac-ctat Dog                             tttgctccgt Opossum                         ttttg-ctat                                 \*\*    \*  \*   CSCS: -0.10846458137740776   - Smug1                 -52    -43  -  CTGATCTCT     - Mouse                           ctgatctct Rat                             cttatc--t Human                           ctaaccgtt                                 \*\* \* \*  \*   CSCS: 1.207605794772062   - cnga3                -104    -95  +  CTTATCTGA     - Mouse                           cttatctga Rat                             cttatctga Human                           ctaaccgga Dog                             ctaaccgga                                 \*\* \* \* \*\*   CSCS: -0.3845013222614813   - ENSMUSG00000044469    -38    -29  -  TGGATCTGA   - Opn1mw                -94    -85  -  CTGATCTCT     - Mouse                           agagatcag Rat                             agagatcag Human                           agagatcag Dog                             agagatcag Opossum                         agagatcag X.tropicalis                    agagataaa                                 \*\*\*\*\*\* \*    CSCS: -1.586745495253383   - ID: Pde6g\_1027\_1034\_10     R|C/ N: (4/5)     Z: 4.442663    Consensus:                           MSATAAGM   - Gnat2                 -41    -33  +  ACATAAAG     - Mouse                           acataaag Rat                             acataaag Human                           atataaag Dog                             atataaag Opossum                         acataaag                                 \* \*\*\*\*\*\*   CSCS: -1.696467562630248   - Gnb3                  -21    -13  -  AGATAAGA   - Pde6c                 -23    -15  +  CCATAAGC     - Mouse                           ccataagc Rat                             ccataagc Human                           ccctaagc Dog                             ctgtaagc Opossum                         cgctaagc Chicken                         cactaagc X.tropicalis                    cactaagc                                    \*\*\*\*\*   CSCS: -1.2203126961822135   - ENSMUSG00000002372    -66    -58  -  CCATAAGC   - Arr3                  -43    -35  +  ATATAAGA     - Mouse                           atataaga Rat                             atataaga Human                           gtataaga Dog                             gtataaaa Opossum                         ccataaaa                                   \*\*\*\* \*   CSCS: -0.32127293892259806   - ID: Gnb3\_1979\_1987\_1     R|C/ N: (5/8)     Z: 4.3349175    Consensus:                           CWCTTATMN   - Gnb3                  -22    -13  +  CTCTTATCT   - Arr3                  -43    -34  -  CTCTTATAT     - Mouse                           atataagag Rat                             atataagag Human                           gtataagag Dog                             gtataaaag Opossum                         ccataaaag                                   \*\*\*\* \*\*   CSCS: -0.5469448206439848   - cnga3                -106    -97  +  CGCTTATCT     - Mouse                           cgcttatct Rat                             cgcttatct Human                           ccctaaccg Dog                             ccctaaccg                                 \* \*\* \* \*    CSCS: 0.15514965635112404   - Gnb3                   44     53  -  CTCTTATGC     - Mouse                           ctcttatgc Rat                             --------- Human                           --------- Dog                             --------- Opossum                         --------- CSCS: 2.7296416491897704   - ENSMUSG00000029821     49     58  +  CTCTTATCA   - ENSMUSG00000028920   -153   -144  -  CTCTTATCA   - Opn1sw               -155   -146  +  CTCTTATCC     - Mouse                           ggataagag- Rat                             ggataagag- Human                           ggataagag- Dog                             ggataaaaat                                 \*\*\*\*\*\* \*     CSCS: -0.7965214354856055   - Opn1sw                 19     28  -  CTCTTATAG     - Mouse                           ctcttatag Rat                             ctcttatgg Human                           ctcttatag Dog                             ctcttatag Opossum                         ctcttatag                                  \*\*\*  \* \*   CSCS: -1.9607435941396139   - Gngt2                 -78    -69  -  GACTTATCT     - Mouse                           agataagtc Rat                             agataagtc Human                           agatccgtc Dog                             agaccagtc                                 \*\*\*   \*\*\*   CSCS: -0.8408718747010308   - ENSMUSG00000029415   -135   -126  -  TCCTTATCT   - ID: cnga3\_317\_327\_2     R|C/ N: (5/9)     Z: 4.050124    Consensus:                           SWCTTATCWSN   - Gnb3                  -22    -11  +  CTCTTATCTCT   - Arr3                  -45    -34  -  CTCTTATATCC     - Mouse                           ggatataagag Rat                             ggatataagag Human                           gggtataagag Dog                             aagtataaaag Opossum                         ggccataaaag                                     \*\*\*\* \*\*   CSCS: -0.43442689109224836   - Gngt2                 -80    -69  -  GACTTATCTCC     - Mouse                           ggagataagtc Rat                             ggagataagtc Human                           ggagatccgtc Dog                             ggagaccagtc                                 \*\*\*\*\*   \*\*\*   CSCS: -1.0025252635162885   - Smug1                 106    117  +  CACTTATCTCA   - ENSMUSG00000029821     49     60  +  CTCTTATCAGT   - ENSMUSG00000026983    160    171  -  TCCTTATCTCT   - ENSMUSG00000028920   -155   -144  -  CTCTTATCAGG   - ENSMUSG00000029415   -137   -126  -  TCCTTATCTCA   - Opn1sw               -155   -144  +  CTCTTATCCTC     - Mouse                           gaggataagag- Rat                             gaggataagag- Human                           gaggataagag- Dog                             gaggataaaaat                                 \*\*\*\*\*\*\*\* \*     CSCS: -1.0088249982463358   - ID: cnga3\_317\_326\_2     R|C/ N: (5/9)     Z: 4.050124    Consensus:                           SWCTTATCWS   - Gnb3                  -22    -12  +  CTCTTATCTC   - Arr3                  -44    -34  -  CTCTTATATC     - Mouse                           gatataagag Rat                             gatataagag Human                           ggtataagag Dog                             agtataaaag Opossum                         gccataaaag                                    \*\*\*\* \*\*   CSCS: -0.4869348110566958   - Smug1                 106    116  +  CACTTATCTC   - ENSMUSG00000029821     49     59  +  CTCTTATCAG   - ENSMUSG00000026983    161    171  -  TCCTTATCTC   - ENSMUSG00000028920   -154   -144  -  CTCTTATCAG   - Gngt2                 -79    -69  -  GACTTATCTC     - Mouse                           gagataagtc Rat                             gagataagtc Human                           gagatccgtc Dog                             gagaccagtc                                 \*\*\*\*   \*\*\*   CSCS: -0.9288965759706529   - ENSMUSG00000029415   -136   -126  -  TCCTTATCTC   - Opn1sw               -155   -145  +  CTCTTATCCT     - Mouse                           aggataagag- Rat                             aggataagag- Human                           aggataagag- Dog                             aggataaaaat                                 \*\*\*\*\*\*\* \*     CSCS: -0.9148097947734568   - ID: Opn1sw\_2006\_2016\_1     R|C/ N: (5/9)     Z: 4.050124    Consensus:                           RRCAGAAGAAW   - Opn1sw                  5     16  +  AGCAGAAGAAT     - Mouse                           attcttctgct Rat                             cttct---gcg Human                           atcct------ Dog                             accat---ccc Opossum                         atgtt----cc CSCS: 1.683099759382308   - Pde6c                -152   -141  -  TGCAGAAGAAT     - Mouse                           attctt--ctgca Rat                             attctt--ctggg Human                           attttt--ctgga Dog                             attttc--ctgga                                 \*\*\* \* \*\*\*\*\*     CSCS: -0.5532553540668963   - Gnat2                -179   -168  -  GCCAGAAGAAT     - Mouse                           attct-----tctggc Rat                             attct----ttctggc Human                           atcct-----tc---- Dog                             atcct----atcctcc                                 \*\* \*\*\*\*\*\* \*\*       CSCS: -0.7248856210336425   - Gngt2                  -2      9  +  GCAAGAAGAAT     - Mouse                           gcaagaag Rat                             gcaagaag Human                           gcaagagg Dog                             gcaagagg                                 \*\*\*\*\*\* \*   Mouse                           at Rat                             at Human                           gt Dog                             at                                  \*   CSCS: 0.13515743019511567   - ENSMUSG00000029452   -156   -145  -  GACAGAAGAAA   - ENSMUSG00000020154     -2      9  +  AGCAGAAGCAG   - cngb3                -112   -101  +  AGCAGAAGTAA     - Mouse                           agcagaagt-aa Rat                             agcagaagt-aa Human                           agcagaagt-at Dog                             agcagaagt-aa Opossum                         agcagaaat-at                                 \*\*\*\*\*\*\* \*\*\*    CSCS: -1.5628487233340338   - ENSMUSG00000034528    -32    -21  -  TGCAGAAGAGG   - ENSMUSG00000054757   -108    -97  -  GACAGAAGAAA   - ID: Arr3\_1026\_1036\_4     R|C/ N: (7/17)     Z: 4.033988    Consensus:                           SMTGGCCCWSN   - Gnb3                 -171   -160  +  CCTGGCCCGCT     - Mouse                           agcgggccag---g Rat                             agccggccag---g Human                           accaggccag---g Dog                             actggtccag---g Opossum                         cacattccagaatg                                       \*\*\*\*   \*   CSCS: -0.35480052804545964   - ENSMUSG00000044469   -592   -581  +  GCTGGCCCTGT   - Opn1sw               -316   -305  -  CCTGGCCCTTT     - Mouse                           cctggcccttt Rat                             cctggcccttt Human                           tctgccccttt Dog                             tctgctccttt                                  \*\*\*   \*\* \*   CSCS: -1.1488964970347266   - ENSMUSG00000053093   -334   -323  -  CCTGGCCCTAT   - ENSMUSG00000053093   -254   -243  -  CAGGGCCCTGG   - cnga3                -160   -149  -  TCTGGCCCTGT     - Mouse                           acag--------ggccaga Rat                             acgg--------ggccaga Human                           gccc--------gg----- Dog                             acgctgggccttgg-----                                  \*          \*\*        CSCS: 1.727352111580352   - ENSMUSG00000037692   -148   -137  -  CCTGGCCCGGA   - ENSMUSG00000030281   -524   -513  -  CCTGGCCCAGG   - ENSMUSG00000029073   -499   -488  +  GCTGGCCCTCC   - ENSMUSG00000037750   -306   -295  +  CCTGGCCCAGC   - ENSMUSG00000062077   -236   -225  -  AGTGGCCCTGA   - Gngt2                -397   -386  +  CTGGGCCCTGG     - Mouse                           ctgggccctgg Rat                             ctgggccctgg Human                           ctgctccccga Opossum                         ttgttcccttg                                  \*\*  \*\*\*      CSCS: 0.7868019297901846   - ENSMUSG00000036537   -565   -554  -  TTGGGCCCTGG   - cngb3                -558   -547  +  CTGGGCCCTGG     - Mouse                           ctgggccctgg Rat                             ttgggctcttg Human                           ctgggctccgg                                  \*\*\*\*\* \*  \*   CSCS: -0.41142531532091975   - ENSMUSG00000024773   -293   -282  +  CCTGGCCCGGA   - ENSMUSG00000028736   -456   -445  -  GCTGGCCCTCA   - Elovl2               -139   -128  -  GCTGGCCCTCG   - Opn1mw               -180   -169  +  AATGGCCCTGA     - Mouse                           aatggcc----------------ctga Rat                             aatggcc----------------ctga Human                           aatccct----------------ctga Dog                             aatcctc----------------ctga Opossum                         agtcccccatctgccacccgtcactgg                                 \* \*                    \*\*\*    CSCS: -1.0760834583836145   - ID: Pde6d\_1105\_1112\_7     R|C/ N: (4/6)     Z: 3.989411    Consensus:                           MWCTGTGK   - Opn1mw                 25     33  -  AACTGTCT     - Mouse                           a--------- Rat                             a--------- Human                           g--------- Dog                             g--------- Opossum                         gacacagaga X.tropicalis                    g---------                                                           \*\* \*        CSCS: -0.6537562697779445   - Gngt2                  50     58  +  AACTGTGG     - Mouse                           aactgtgg Rat                             aactgtgg Human                           gggtgtta Dog                             aagtgtaa                                    \*\*\*     CSCS: 0.5020646257125257   - Gngt2                  29     37  -  AGCTGTGT     - Mouse                           acacagct Rat                             acacagct Human                           acatgacc Dog                             --acatcc                                   \*   \*    CSCS: 2.275041264053308   - ENSMUSG00000047759     51     59  -  CACTGTGC   - Smug1                  28     36  -  CTCTGTGT     - Mouse                           ctctgtgt Rat                             ctttgtgt Human                           ctatatct Dog                             ctgtgtcc                                 \*\* \* \*     CSCS: -2.1943652690824913   - cnga3                  36     44  +  GACTGTGG     - Mouse                           gactgtgg Rat                             agctgggg Human                           g------- Dog                             ggccgggt CSCS: 0.15505083183339102   - ENSMUSG00000016526     52     60  +  CTCTGTGT   - ID: Smug1\_2505\_2515\_3     R|C/ N: (4/6)     Z: 3.989411    Consensus:                           NYSATTCTKMW   - Gnat2                -182   -171  +  TGCATTCTTCT     - Mouse                           tgcattct-----tct Rat                             ctcattct----ttct Human                           --tatcct-----tc- Dog                             cacatcct----atcc                                    \*\* \*\*\*\*\*\* \*\*    CSCS: -0.4542996328285501   - ENSMUSG00000046840     93    104  +  GCCATTCTGTA   - Gngt2                   1     12  -  CTGATTCTTCT     - Mouse                           agaag Rat                             agaag Human                           agagg Dog                             agagg                                 \*\*\* \*   Mouse                           atcag Rat                             atcag Human                           gtcag Dog                             atcag                                  \*\*\*\*   CSCS: -0.7482869642846863   - Pde6c                -155   -144  +  GTTATTCTTCT     - Mouse                           gttattctt--ct Rat                             gttattctt--ct Human                           gttattttt--ct Dog                             gtgattttc--ct                                 \*\* \*\*\* \* \*\*\*\*   CSCS: -1.2366884385024741   - ENSMUSG00000020154    -80    -69  -  GCCATTCTGAC   - Opn1sw                  8     19  -  ACGATTCTTCT     - Mouse                           acgattcttct Rat                             actcttct--- Human                           gtgatcct--- Dog                             attaccat--- Opossum                         attatgtt--- CSCS: 1.5672901429110484   - ID: Pde6a\_841\_848\_14     R|C/ N: (4/6)     Z: 3.989411    Consensus:                           YYTTAAKW   - ENSMUSG00000043760    -80    -72  +  CGTTAATT   - ENSMUSG00000043760    -73    -65  -  GTTTAATA   - cngb3                 -93    -85  +  TTTTAAGT     - Mouse                           tttt Rat                             tttt Human                           tttt Dog                             tttt Opossum                         ctta                                  \*\*    Mouse                           agt Rat                             agc Human                           agc Dog                             agc Opossum                         aat Chicken                         agc                                 \*     CSCS: 0.5051564927011539   - Gnat2                 -72    -64  +  TTTTAATT     - Mouse                           t-tttaatt Rat                             t-tttaatt Human                           t-cttaatt Dog                             t-cttaatt Opossum                         tccttaatt                                 \*  \*\*\*\*\*\*   CSCS: -1.4915936110776333   - cnga3                -116   -108  -  TTTTAAGG     - Mouse                           ccttaaaa Rat                             ccttaaaa Human                           ctctgcaa Dog                             ccttgcaa                                 \*  \*  \*\*   CSCS: -0.17150363449065706   - ENSMUSG00000024906   -110   -102  +  TTTTAACA   - Opn1mw                -99    -91  -  TCTTAATT     - Mouse                           aattaaga Rat                             aattaaga Human                           aattaaga Dog                             aattaaga Opossum                         aattaaga X.tropicalis                    aattaaga                                 \*\*\*\*\*\*\*\*   CSCS: -1.8253065051916892   - ID: Smug1\_2506\_2516\_3     R|C/ N: (4/6)     Z: 3.989411    Consensus:                           NYNTTCTTCTK   - ENSMUSG00000055652   -308   -297  -  GCATTCTTCTT   - Gnat2                -181   -170  +  GCATTCTTCTG     - Mouse                           gcattct-----tctg Rat                             tcattct----ttctg Human                           -tatcct-----tc-- Dog                             acatcct----atcct                                   \*\* \*\*\*\*\*\* \*\*     CSCS: -0.5895926269310964   - ENSMUSG00000032232   -300   -289  -  CCATTCTTCCA   - cnga3                -216   -205  -  TCTTTCTTCTT     - Mouse                           aagaagaaag------a Rat                             ----------------- Human                           -------aaa------a Dog                             -------caa------a                                           \*\*\*\*\*\*    CSCS: 2.234947438175649   - Pde6c                -154   -143  +  TTATTCTTCTG     - Mouse                           ttattctt--ctg Rat                             ttattctt--ctg Human                           ttattttt--ctg Dog                             tgattttc--ctg                                 \* \*\*\* \* \*\*\*\*\*   CSCS: -1.2366884385024741   - Arr3                 -279   -268  +  TTCTTCTTCTT     - Mouse                           ttcttcttctt Rat                             ttc--------                                 \*\*\*           CSCS: 2.3795523032727055   - Arr3                 -282   -271  +  ATGTTCTTCTT     - Mouse                           atgttcttctt Rat                             atgttc-----                                 \*\*\*\*\*\*        CSCS: 1.0952542127295555   - ID: Elovl2\_230\_237\_9     R|C/ N: (4/6)     Z: 3.989411    Consensus:                           NNAATTMW   - ENSMUSG00000043760    -78    -70  +  TTAATTAT   - ENSMUSG00000043760   -157   -149  +  TTAATTCT   - Opn1mw               -101    -93  +  CCAATTAA     - Mouse                           ccaattaa Rat                             ccaattaa Human                           ccaattaa Dog                             ccaattaa Opossum                         tcaattaa X.tropicalis                    ccaattaa                                  \*\*\*\*\*\*\*   CSCS: -1.6669343231235867   - ENSMUSG00000025329    -34    -26  +  GAAATTAA   - cngb3                 -53    -45  +  GGAATTAA     - Mouse                           ggaattaa Rat                             ggaattaa Human                           ggaattaa Dog                             ggaattaa Opossum                         ggaattaa Chicken                         ggcatcaa                                 \*\* \*\* \*\*   CSCS: -1.3202457270951442   - Gnat2                 -70    -62  +  TTAATTGG     - Mouse                           ttaattgg Rat                             ttaattgg Human                           ttaattgg Dog                             ttaattgg Opossum                         ttaattgg                                 \*\*\*\*\*\*\*\*   CSCS: -2.1062154657354775   - Arr3                 -130   -122  +  CTAATTAT     - Mouse                           ctaattat-- Rat                             ctaattat-- Human                           ctatttat-- Dog                             ctatttataa Opossum                         ctctttgg--                                 \*\*  \*\*       CSCS: -0.32127293892259806 |

Page by: Charles Danko & Maochun Qin; SUNY Upstate Medical University.
