# Supplementary material for: Bioinformatic identification of novel putative photoreceptor specific cis-elements
Source: BMC Bioinformatics. 2007 Oct 22;8:407. doi: 10.1186/1471-2105-8-407 (PMC2225425; doi:10.1186/1471-2105-8-407)
Supplement: Additional file 1 — Explanation of Supplementary Data. Detailed information on reading HTML formatted supplementary data. [file 1471-2105-8-407-S1.ZIP › c.TS.html]

cis-Browser 

Predictions via cis-Browser

|  |
| --- |
| - ID: Opn1sw\_257\_268     R|C/ N: (5/6)     Z: 3.9212346    Consensus:                           RRMTMAGGTTGG   - Opn1sw              -1744  -1732  +  GGCTAAGGTTGG     - Mouse                           ccaaccttagcc Rat                             tcaaccttagcc                                  \*\*\*\*\*\*\*\*\*\*\*   CSCS: -0.9132511298138928   - cnga3                -140   -128  +  GGCTAGGGTTGG     - Mouse                           ggct--agggttgg Rat                             g--------gctag Human                           gctc--ggtgatgg Dog                             gctt--gggtattg                                 \*   \*\*     \* \*   CSCS: 2.4109072373097695   - ENSMUSG00000024124   -336   -324  +  AAATAAGGTTGG   - Pde6h                -861   -849  -  TTATAAGGTTGG     - Mouse                           ccaacc Rat                             ccaacc Human                           ttaatt Dog                             ctaact                                   \*\*     Mouse                           tataa- Rat                             tccca- Human                           tattt- Dog                             tatat-                                 \*    \*   CSCS: 0.5741984536820928   - Pde6c               -1175  -1163  -  GGCTGAGGTTGC     - Mouse                           gc Rat                             gc Human                           gc Dog                             gc                                 \*\*   Mouse                           acctcagc------c Rat                             gtctcaac------c Human                           acctcaacggatctc Dog                             atctcagtggatctc                                   \*\*\*\*        \*   CSCS: -1.1318600309305864   - Elovl2               -563   -551  +  AGCTCAGGTTGG     - Mouse                           ccaacctgagct Rat                             ccaacctgagct                                 \*\*\*\*\*\*\*\*\*\*\*\*   CSCS: -1.7379995793051286   - ID: Smug1\_2655\_2668     R|C/ N: (4/4)     Z: 3.8508577    Consensus:                           MTTCAATTTGCCTT   - Smug1                 654    668  +  CTTCAATTTGCCTT     - Mouse                           aaggcaaattgaag Rat                             aaggcagga-gaag                                 \*\*\*\*\*\*    \*\*\*\*   CSCS: 0.6006972905085493   - Arr3                -1522  -1508  +  GTTCAATTTGTCTT     - Mouse                           g---ttcaatttgtctt Rat                             aatgttcagtccatcta Human                           -----------tgtcct Dog                             -----------cattct                                              \*      CSCS: 2.8502248516128192   - Pde6h                -221   -207  +  ATTCAATGTGCCTT     - Mouse                           attcaatgtgcctt Rat                             ccttaatgtctctt Human                           atacaatacctatt Dog                             atttaatacctact                                     \*\*\*      \*   CSCS: 0.23949866306647066   - Opn1mw              -1388  -1374  +  CATCAATTTGCCCC     - Mouse                           catcaatttgcccc-------- Rat                             cttcaatcacctccccctacac                                 \* \*\*\*\*\*   \* \*\*           CSCS: 2.397478973577583   - ID: Pde6c\_692\_704     R|C/ N: (4/4)     Z: 3.8508577    Consensus:                           MATGTGGGCACCT   - Pde6c               -1309  -1296  +  AATGTGGGCACCT     - Mouse                           aatgtgggcacct Rat                             aatgtgggcaccg Human                           aatatgtgcacct Dog                             aaggtatgtactt                                 \*\*  \*  \* \*\*     CSCS: -1.3698214610723716   - Gnb3                -1824  -1811  +  CACGTGGGCACCT     - Mouse                           AGGTGCCCACGTG Rat                             AGGTGCCCAGGTG Human                           AGGTGCCCACGTG Dog                             AGGCGCCCAGGTG Opossum                         TGGGGCCCGAGTG Chicken                         TACGGAGTGCGTG X.tropicalis                    TGCTGACTGGGTG                                           \*\*\*   CSCS: 0.7556960813331745   - Smug1               -1864  -1851  +  AATGTGGGCAGGT     - Mouse                           ac--ctgcccacatt Rat                             ----ctgcccacact Human                           ac--ttttccacact Dog                             gc--ccgcccacacc                                   \*\*    \*\*\*\*\*     CSCS: -0.043218473088997074   - Opn1mw               -642   -629  +  CATGTGAGCACCT     - Mouse                           catgtgagcacct Rat                             caagtaagcatct Human                           tgagcaggcgctg                                    \*   \*\*       CSCS: 0.6459995187231412   - ID: Gngt2\_844\_852\_4     R|C/ N: (7/10)     Z: 5.116412    Consensus:                           MMAATCCWC   - Gngt2                -191   -182  -  AAAATCCAC     - Mouse                           gtggatttt Rat                             gtggatttt Human                           gtggatttt Dog                             gtggatttt Opossum                         atggttttt                                  \*\*\* \*\*\*\*   CSCS: -1.2734106060818053   - ENSMUSG00000027120    -54    -45  +  TAAATCCAC   - ENSMUSG00000054630     -7      2  -  AAAATCCTT   - Smug1                   0      9  -  ATAATCCAC     - Mouse                           at---aatccac Rat                             at---aatccac Human                           gt---cctccat                                  \*\*\*\*  \*\*\*\*    CSCS: 0.02466961309927132   - cnga3                 -18     -9  +  CCAATCCAC     - Mouse                           ccaatcca Rat                             ccagccca Human                           ccctgggg Dog                             ccctagga                                 \*\*                                         Mouse                                 Rat                                 Human                                 Dog CSCS: NaN   - cnga3                -112   -103  +  AAAATCCGC     - Mouse                           aaaatccgc Rat                             aaaatccgc Human                           gcaatcccc Dog                             gcaatcccc                                   \*\*\*\*\* \*   CSCS: -0.3845013222614813   - Pde6c                 -45    -36  +  TTAATCCAC     - Mouse                           ttaatccac Rat                             ttaatccac Human                           ttaatcctg Dog                             ttaatcctg Opossum                         taaagtcac Chicken                         ttaattagc X.tropicalis                    ttaattagc                                    \*        CSCS: -0.5210093513807389   - Pde6c                 -71    -62  -  AAAATCCTC     - Mouse                           ga--ggatttt Rat                             ta-aggatttt Human                           ca-gggattta Dog                             ta-gggatttc Opossum                         cacagggctta Chicken                         ta-ggggttta X.tropicalis                    ca-ttggttta                                  \*       \*    CSCS: -0.5210093513807389   - cngb3                 -38    -29  +  AAAATCCTG     - Mouse                           aaaatcctg Rat                             aaaatcctg Human                           aaaatcctg Dog                             aaaatcccg Opossum                         aaaatcctg Chicken                         taaa-cctg                                  \*\*\* \*\* \*   CSCS: -1.23590193995445   - ENSMUSG00000029630   -235   -226  -  CAAATCCAT   - Pde6h                  12     21  -  AAAATCCCC     - Mouse                           gggg---atttt Rat                             tg---aattttt Human                           tgagtacttctg Dog                             tgagtatttctc                                  \*      \* \*    CSCS: 1.0907125517312193   - Opn1mw               -160   -151  -  CCAATCCAC     - Mouse                           gtggattgg Rat                             gtgggttgg Human                           gcgggctcg Dog                             --------- Opossum                         gc---ttca CSCS: 1.4098661763835578   - ID: Gnb1\_573\_584\_3     R|C/ N: (4/4)     Z: 4.5303755    Consensus:                           NGGGRTTGGGGR   - Arr3                 -102    -90  -  TAGGGTTGGGGT     - Mouse                           acccc------aa--cccta Rat                             acccc------aa--cccta Human                           cccac--caagaa--cccta Dog                             ccccc------aa--ctcta Opossum                         actca------ga--gccaa                                  \*   \*\*     \*\*\*  \* \*   CSCS: -0.3865455969240813   - Gnb3                   21     33  -  GGGGGGTGGGGA     - Mouse                           ggggggt---gggga Rat                             aagaagg---gggga Human                           gagagga---ggggg Dog                             ggggaag---agggg Opossum                         ggtaggg---gagga                                        \*\*\*  \*\*    CSCS: -0.1274078077346833   - Gngt2                -112   -100  +  CGGGATTGGGGA     - Mouse                           cgggattgggga Rat                             cgggattgggaa Human                           agggacagggga Dog                             agggcctgggga Opossum                         gtgggctagtga                                   \*\*    \*  \*   CSCS: 0.09401709123379734   - Opn1mw                -78    -66  +  AGGGTTTGGGGG     - Mouse                           a Rat                             a Human                           a Dog                             a Opossum                         a X.tropicalis                    - Mouse                           ggtttggg-gg Rat                             ggtttggg-gg Human                           ggtttggg-ag Dog                             ggtttggg-ag Opossum                         catttggggag X.tropicalis                    agtttaag-gg                                   \*\*\*  \*  \*   CSCS: -1.1687114754638788   - ID: Gnat1\_444\_454\_2     R|C/ N: (4/4)     Z: 4.5303755    Consensus:                           KMCCAAGGGCW   - Gnb3                 -339   -328  +  GCCCAAGGGCA     - Mouse                           tgccctt-ggg------c Rat                             tgcccct-agg------c Human                           tgcctct-ggg------t Dog                             tgcctct-ggg------t Opossum                         tgtcctc-agg------t                                 \*\*     \* \*\*          CSCS: -0.9298822203561773   - Elovl2               -443   -432  -  GACCAAGGGCT     - Mouse                           gaccaagggct Rat                             gtccaagg-tt                                 \* \*\*\*\*\*\*  \*   CSCS: 0.632654862970378   - Opn1sw               -408   -397  +  TCCCAAGGGGG     - Mouse                           cccccttggga------------ Rat                             gccccctcggga----------- Human                           gtgccctggaa------------ Dog                             gcatcctggggagcaccctggga                                     \* \* \*                 CSCS: 0.1386198355788887   - Opn1mw               -266   -255  -  TTACAAGGGCT     - Mouse                           agcccttgtaa Rat                             --ctcttgtaa Human                           --------taa Opossum                         --------taa                                         \*\*\*   CSCS: 0.7873423985446587   - ID: Opn1mw\_1904\_1915\_1     R|C/ N: (4/4)     Z: 4.5303755    Consensus:                           WTAAGAGATCAG   - Opn1mw                -97    -85  +  TTAAGAGATCAG     - Mouse                           ttaagagatcag Rat                             ttaagagatcag Human                           ttaagagatcag Dog                             ttaagagatcag Opossum                         ttaagagatcag X.tropicalis                    ttaagagataaa                                 \*\*\*\*\*\*\*\*\* \*    CSCS: -1.7901212098465105   - cnga3                -122   -110  -  TTAAGGGATCAG     - Mouse                           ctgatcccttaa Rat                             ctgatcccttaa Human                           ctgatcctctgc Dog                             ctgatcccttgc                                 \*\*\*\*\*\*\*  \*     CSCS: -0.9309443787631781   - Opn1sw                -94    -82  +  CTAAGAGATCTC     - Mouse                           gagatctcttag Rat                             gagatctcttag Human                           gaaatccctaaa Dog                             gagctgtccaag Opossum                         gggacccttgag                                 \*         \*    CSCS: -0.42937732572149695   - Smug1                 -55    -43  +  AGTAGAGATCAG     - Mouse                           ctgatctctact Rat                             cttatc--tact Human                           ctaaccgttagt                                 \*\* \* \*  \*\* \*   CSCS: 0.8540536158360592   - ID: Gnat1\_443\_453\_4     R|C/ N: (4/4)     Z: 4.5303755    Consensus:                           NGCCCAAGGGS   - Gnb3                 -340   -329  +  GGCCCAAGGGC     - Mouse                           gccctt-ggg------cc Rat                             gcccct-agg------cc Human                           gcctct-ggg------tc Dog                             gcctct-ggg------tc Opossum                         gtcctc-agg------tc                                 \*     \* \*\*       \*   CSCS: -0.9298822203561773   - Gnat2                -304   -293  -  AGCCCAAGTTG     - Mouse                           caacttggg--------------- Rat                             ------------------------ Human                           caacataaggaatgctttctacat Dog                             caggatgggcagagcttcctgcat Mouse                           t Rat                             - Human                           t Dog                             t CSCS: -0.27565781565325415   - Opn1sw               -409   -398  +  CTCCCAAGGGG     - Mouse                           ccccttggga------------g Rat                             ccccctcggga-----------g Human                           tgccctggaa------------g Dog                             catcctggggagcaccctgggag                                    \* \* \*              \*   CSCS: -0.6872420017432251   - Elovl2               -442   -431  -  TGACCAAGGGC     - Mouse                           tgaccaagggc Rat                             tgtccaagg-t                                 \*\* \*\*\*\*\*\*     CSCS: 0.632654862970378   - ID: Pde6b\_737\_747\_6     R|C/ N: (5/7)     Z: 4.244245    Consensus:                           NMAGATGCWNN   - ENSMUSG00000028553   -398   -387  +  ACAGATGCTTA   - Elovl2               -334   -323  +  CCTGATGCTGT     - Mouse                           acagcatcagg Rat                             --------agg Human                           --------gag                                           \*   CSCS: 2.7472635426234073   - cnga3                -449   -438  -  GAAGATGCTGC     - Mouse                           gcagc-------atcttc Rat                             ------------------ Human                           ------------------ Dog                             gccgggccccatacctcc CSCS: 1.9283439499761006   - Gngt2                -288   -277  -  ACAGATGCCCC     - Mouse                           ggggcatctgt Rat                             ggggcatctgt Human                           ggggcagctgt Dog                             ------gataa Opossum                         -----agctga                                         \*     CSCS: 1.0017037565683957   - Opn1sw               -255   -244  -  GGAGATGCTGC     - Mouse                           ggagatgctgc Rat                             ggagatgctgc Human                           gaagatgcttc Dog                             agagctgtggc                                   \*\* \*\*   \*   CSCS: -1.2468507593302933   - Pde6h                -234   -223  -  ACAGATGCATG     - Mouse                           ca--tgcatctgt Rat                             tg--tgcatctgt Human                           tg--tgtttttgt Dog                             tg--tgtgtttgt                                   \*\*\*\*  \* \*\*\*   CSCS: -0.06969667942256587   - ENSMUSG00000021346   -171   -160  +  CCAGATGCTCA   - ID: Arr3\_1026\_1036\_4     R|C/ N: (6/10)     Z: 4.186514    Consensus:                           SMTGGCCCYSN   - ENSMUSG00000024124   -247   -236  -  CCTGGCCCCAC   - Gnb3                 -171   -160  +  CCTGGCCCGCT     - Mouse                           agcgggccag---g Rat                             agccggccag---g Human                           accaggccag---g Dog                             actggtccag---g Opossum                         cacattccagaatg                                       \*\*\*\*   \*   CSCS: -0.35480052804545964   - ENSMUSG00000004341   -370   -359  +  CCTGGCCCAGC   - Opn1sw               -316   -305  -  CCTGGCCCTTT     - Mouse                           cctggcccttt Rat                             cctggcccttt Human                           tctgccccttt Dog                             tctgctccttt                                  \*\*\*   \*\* \*   CSCS: -1.1488964970347266   - cnga3                -160   -149  -  TCTGGCCCTGT     - Mouse                           acag--------ggccaga Rat                             acgg--------ggccaga Human                           gccc--------gg----- Dog                             acgctgggccttgg-----                                  \*          \*\*        CSCS: 1.727352111580352   - Gngt2                -397   -386  +  CTGGGCCCTGG     - Mouse                           ctgggccctgg Rat                             ctgggccctgg Human                           ctgctccccga Opossum                         ttgttcccttg                                  \*\*  \*\*\*      CSCS: 0.7868019297901846   - ENSMUSG00000024233   -115   -104  +  GGAGGCCCTGG   - ENSMUSG00000025347   -243   -232  +  CCTGGCCCCCT   - Elovl2               -139   -128  -  GCTGGCCCTCG   - Opn1mw               -180   -169  +  AATGGCCCTGA     - Mouse                           aatggcc----------------ctga Rat                             aatggcc----------------ctga Human                           aatccct----------------ctga Dog                             aatcctc----------------ctga Opossum                         agtcccccatctgccacccgtcactgg                                 \* \*                    \*\*\*    CSCS: -1.0760834583836145   - ID: Gnb3\_2233\_2243\_2     R|C/ N: (5/8)     Z: 3.9437225    Consensus:                           RGGGCCAGGWG   - Gnb3                 -173   -162  -  CGGGCCAGGAG     - Mouse                           cgggccag---gag Rat                             ccggccag---ggg Human                           caggccag---gag Dog                             tggtccag---gag Opossum                         cattccagaatggg                                     \*\*\*\*   \* \*   CSCS: -0.7208901638014564   - ENSMUSG00000024124   -245   -234  +  GGGGCCAGGTA   - Opn1sw               -314   -303  +  AGGGCCAGGTG     - Mouse                           cacctggccct Rat                             cgcctggccct Human                           catctgcccct Dog                             catctgctcct                                 \*  \*\*\*   \*\*   CSCS: -0.9397043602914096   - Gngt2                -387   -376  -  AGGCCCAGGCC     - Mouse                           ggcc----tgggcct---- Rat                             ggcc----tgggcct---- Human                           agcc----tgtgtct---- Opossum                         ggccacctcctgact----                                  \*\*\*       \* \*\*\*\*\*\*   CSCS: 0.43424587381862634   - cnga3                -158   -147  +  AGGGCCAGAAG     - Mouse                           ag--------ggccagaag------ Rat                             gg--------ggccaga-g------ Human                           cc--------gg------g------ Dog                             gctgggccttgg------g------                                           \*\*      \*\*\*\*\*\*\*   CSCS: 2.226226367632439   - ENSMUSG00000025347   -245   -234  -  GGGGCCAGGTG   - Elovl2               -137   -126  +  AGGGCCAGCGG     - Mouse                           ccgctggccct Human                           tcgccggccgc                                  \*\*\* \*\*\*\*     CSCS: 1.4272438451448117   - ENSMUSG00000004341   -372   -361  -  TGGGCCAGGAG   - ID: cnga3\_317\_329\_4     R|C/ N: (4/5)     Z: 3.8964052    Consensus:                           MTCTTMTCTCCAM   - Arr3                  -47    -34  -  CTCTTATATCCAC     - Mouse                           gtggatataagag Rat                             gtggatataagag Human                           gtgggtataagag Dog                             tcaagtataaaag Opossum                         taggccataaaag                                       \*\*\*\* \*\*   CSCS: 0.11435018640456908   - Gngt2                 -82    -69  -  GACTTATCTCCAT     - Mouse                           atggagataagtc Rat                             atggagataagtc Human                           atggagatccgtc Dog                             gtggagaccagtc                                  \*\*\*\*\*\*   \*\*\*   CSCS: -1.0228091654512244   - Gnb3                  -22     -9  +  CTCTTATCTCTGC   - ENSMUSG00000030945    -18     -5  +  CTCTTCTCTCCAC   - Smug1                  91    104  -  ATCTTGTCTCCAA   - ID: Pde6d\_2007\_2017\_4     R|C/ N: (4/5)     Z: 3.8964052    Consensus:                           WGRGGGAGASG   - ENSMUSG00000054446    134    145  -  GGAGGGAGAGG   - cnga3                 128    139  -  AGAGGGAGATG     - Mouse                           catct---------------ccctct Rat                             tgtct---------------cccttt Human                           aatct---------------ccctac Dog                             aacccctcaattcacaactgccctac                                    \*                \*\*\*\*     CSCS: -0.9331392177202974   - Gngt2                 168    179  -  AGAGGGAGACC     - Mouse                           acaggaaggcc Rat                             acaggaaggcc Human                           acaggaaggac Dog                             ataggaaggaa                                 \* \*\*\*\*\*\*\*     CSCS: -1.4801072634185806   - Smug1                 177    188  -  TCTGGGAGAGG   - Gnb3                  140    151  -  TGGGGGAGAGG     - Mouse                           tgggg----- Rat                             taggg----- Human                           tgggg----- Dog                             tgtgg----- Opossum                         taagg-----                                 \*  \*\*\*\*\*\*\*\*\*\*\*\*\*\*\*\*    \*\*\*\*\*\*\*\*\*\*\*\*\*\*    CSCS: -0.028567612961372283   - ID: Pde6b\_1105\_1112\_13     R|C/ N: (4/5)     Z: 3.8964052    Consensus:                           YYAATCYR   - Gngt2                -190   -182  -  AAAATCCA     - Mouse                           tggatttt Rat                             tggatttt Human                           tggatttt Dog                             tggatttt Opossum                         tggttttt                                 \*\*\* \*\*\*\*   CSCS: -1.397272105155557   - cngb3                -188   -180  +  TTAATCCT     - Mouse                           tt---aatcct Rat                             tt---aatcct Human                           tt---aatcct Dog                             tt---aatcct Opossum                         ct---aagcct                                  \*\*\*\*\*\* \*\*\*   CSCS: -1.3767343569257464   - ENSMUSG00000041333   -184   -176  +  TCAATCCA   - Opn1mw               -159   -151  -  CCAATCCA     - Mouse                           tggattgg Rat                             tgggttgg Human                           cgggctcg Dog                             -------- Opossum                         c---ttca CSCS: 1.646481240079476   - Arr3                 -162   -154  +  CTAATCTG     - Mouse                           ctaatctg- Rat                             ctaatctg- Human                           ctaatctg- Dog                             ttaatctg- Opossum                         ataagctc-                                  \*\*\* \*\* \*   CSCS: -0.9179226826359946   - Arr3                 -176   -168  +  CTAATCTG     - Mouse                           ctaatc-tg Rat                             ctaatc-tg Human                           ctaatcttt Dog                             ctaatcttt Opossum                         ctaatctct                                 \*\*\*\*\*\*      CSCS: -0.9179226826359946   - ID: Pde6b\_1307\_1314\_2     R|C/ N: (4/5)     Z: 3.8964052    Consensus:                           NTGATATM   - ENSMUSG00000069763     23     31  +  GTGATATC   - ENSMUSG00000069763     25     33  -  TCGATATC   - Smug1                  56     64  +  TTGATATG   - Opn1mw                -30    -22  +  CTGATATA     - Mouse                           ctgatat Rat                             ctgatat Human                           ccagtat Dog                             ctggtat Opossum                         ctggtat X.tropicalis                    ttggtat                                     \*\*\*                                   Mouse                                 Rat                                 Human                                 Dog                                 Opossum CSCS: NaN   - Opn1sw                  0      8  +  GTGATAGC   - Pde6h                 -53    -45  -  CTGATATA     - Mouse                           tatatcag Rat                             tatatcag Human                           taagtcag Dog                             taaatcag                                 \*\*  \*\*\*\*   CSCS: -0.9137206958370295   - ID: Pde6c\_835\_844\_5     R|C/ N: (4/5)     Z: 3.8964052    Consensus:                           CTCTCTCTCT   - Pde6h                -298   -288  -  TTCTCTCTCT     - Mouse                           agagagaga-a Rat                             aatggagaa-- Human                           aataaaaaa-a Dog                             aatggagga--                                 \*       \*\*    CSCS: 1.003770910116107   - Pde6h                -300   -290  -  CTCTCTCTCT     - Mouse                           agagagagag Rat                             gtaatggaga Human                           agaataaaaa Dog                             ggaatggagg                                   \*          CSCS: 1.1549869381519566   - Pde6h                -302   -292  -  CTCTCTCTCT     - Mouse                           agagagagag Rat                             g-gtaatgga Human                           a-agaataaa Dog                             g-ggaatgga                                     \*        CSCS: 1.3062029661878065   - Pde6h                -304   -294  -  CTCTCTCTCT     - Mouse                           agagagagag Rat                             ttg-gtaatg Human                           gta-agaata Dog                             gtg-ggaatg                                       \*      CSCS: 1.4574189942236564   - Pde6h                -306   -296  -  CTCTCTCTCT     - Mouse                           agagagagag Rat                             aattg-gtaa Human                           aggta-agaa Dog                             cagtg-ggaa                                         \*    CSCS: 1.3062029661878065   - Pde6h                -308   -298  -  CTCTCTCTCT     - Mouse                           agagagagag Rat                             aaaattg-gt Human                           aaaggta-ag Dog                             aacagtg-gg                                 \*            CSCS: 1.3062029661878065   - Pde6h                -310   -300  -  CTCTCTCTCT     - Mouse                           agagagagag Rat                             aaaaaattg- Human                           ctaaaggta- Dog                             ctaacagtg-                                   \*          CSCS: 1.6086350222595063   - Pde6h                -312   -302  -  CTCTCTCTCT     - Mouse                           agagagagag Rat                             aaaaaaaatt Human                           atctaaaggt Dog                             tactaacagt                                     \*        CSCS: 1.4574189942236564   - Pde6h                -314   -304  -  CTCTCTCTCT     - Mouse                           agagagagag Rat                             gcaaaaaaaa Human                           aaatctaaag Dog                             agtactaaca                                       \*      CSCS: 1.003770910116107   - Pde6h                -316   -306  -  CTCTCTCTCT     - Mouse                           agagagagag Rat                             aagcaaaaaa Human                           tgaaatctaa Dog                             caagtactaa                                         \*    CSCS: 1.1549869381519566   - Pde6h                -318   -308  -  CTCTCTCTCT     - Mouse                           agagagagag Rat                             aaaagcaaaa Human                           attgaaatct Dog                             agcaagtact                                 \*            CSCS: 1.003770910116107   - Pde6h                -320   -310  -  CTCTCTCTCT     - Mouse                           agagagagag Rat                             gtaaaagcaa Human                           gtattgaaat Dog                             gtagcaagta                                   \*          CSCS: 1.1549869381519566   - Pde6h                -322   -312  -  CTCTCTCTCT     - Mouse                           agagagagag Rat                             tagtaaaagc Human                           gggtattgaa Dog                             gggtagcaag                                     \*        CSCS: 1.1549869381519566   - Pde6h                -324   -314  -  CTCTCTCTCT     - Mouse                           ag-------- Rat                             att------- Human                           gg-------- Dog                             gag-------                                    \*\*\*\*\*\*\*\*\*\*\*\*\*\*\*\*\*\*\*\*\*\*\*\*\*\*\*\*\*\*    \*      CSCS: 1.3062029661878065   - Pde6h                -326   -316  -  CTCTCTCTCT     - Mouse                           a--------- Rat                             a--------- Human                           a--------- Dog                             a---------                                 \*\*\*\*\*\*\*\*\*\*\*\*\*\*\*\*\*\*\*\*    \*\*\*\*\*\*\*\*\*\*\*\*\*\*\*\*\*\*\*\*\*\*\*\*\*\*\*\*\*\*    \*    CSCS: 1.003770910116107   - Pde6h                -328   -318  -  CTCTCTCTCT     - Mouse                           aga------- Rat                             ata------- Human                           aaa------- Dog                             aaa-------                                 \* \*\*\*\*\*\*\*\*\*\*\*\*\*\*\*\*\*\*\*\*    \*\*\*\*\*\*\*\*\*\*\*\*\*\*\*\*\*\*\*\*\*\*\*\*\*\*\*\*\*\*       CSCS: 1.1549869381519566   - Pde6h                -330   -320  -  CTCTCTCTCT     - Mouse                           agaga----- Rat                             aaata----- Human                           agaaa----- Dog                             aaaaa-----                                 \* \* \*\*\*\*\*\*\*\*\*\*\*\*\*\*\*\*\*\*\*\*    \*\*\*\*\*\*\*\*\*\*\*\*\*\*\*\*\*\*\*\*\*\*\*\*\*\*\*\*\*\*     CSCS: 0.5501228260085576   - Pde6h                -332   -322  -  CTCTCTCTCT     - Mouse                           agagaga--- Rat                             --aaata--- Human                           --agaaa--- Dog                             --aaaaa---                                   \* \* \*\*\*\*\*\*\*\*\*\*\*\*\*\*\*\*\*\*\*\*    \*\*\*\*\*\*\*\*\*\*\*\*\*\*\*\*\*\*\*\*\*\*\*\*\*\*\*\*\*\*   CSCS: 0.852554882080257   - Pde6h                -334   -324  -  CTCTCTCTCT     - Mouse                           agagagaga-------------------g Rat                             ----aaata-------------------g Human                           ----agaaa-------------------t Dog                             ----aaaaa-------------------t                                     \* \* \*\*\*\*\*\*\*\*\*\*\*\*\*\*\*\*\*\*\*\*    CSCS: 1.1549869381519566   - Pde6h                -336   -326  -  CTCTCTCTCT     - Mouse                           agagagagag Rat                             ------aaat Human                           ------agaa Dog                             ------aaaa                                       \* \*    CSCS: 1.7598510502953555   - Pde6h                -338   -328  -  CTCTCTCTCT     - Mouse                           agagagagag Rat                             --------aa Human                           --------ag Dog                             --------aa                                         \*    CSCS: 2.2134991344029054   - Pde6h                -340   -330  -  CTCTCTCTCT     - Mouse                           agagagagag Rat                             ---------- Human                           ---------- Dog                             ---------- CSCS: 2.8183632465463044   - Pde6h                -342   -332  -  CTCTCTCTCT     - Mouse                           agagagagag Rat                             ---------- Human                           ---------- Dog                             ---------- CSCS: 2.8183632465463044   - Pde6h                -344   -334  -  CTCTCTCTCT     - Mouse                           agagagagag Rat                             ---------- Human                           ---------- Dog                             ---------- CSCS: 2.8183632465463044   - Pde6h                -346   -336  -  CTCTCTCTCT     - Mouse                           agagagagag Rat                             ---------- Human                           ---------- Dog                             ---------- CSCS: 2.8183632465463044   - Pde6h                -348   -338  -  CTCTCTCTCT     - Mouse                           agagagagag Rat                             ---------- Human                           ---------- Dog                             ---------- CSCS: 2.8183632465463044   - Pde6h                -350   -340  -  CTCTCTCTCT     - Mouse                           agagagagag Rat                             a--------- Human                           a--------- Dog                             a---------                                 \*            CSCS: 2.3647151624387552   - Pde6h                -352   -342  -  CTCTCTCTCT     - Mouse                           agagagagag Rat                             gaa------- Human                           gga------- Dog                             gga-------                                   \*          CSCS: 2.0622831063670555   - Pde6h                -354   -344  -  CTCTCTCTCT     - Mouse                           agagagagag Rat                             aggaa----- Human                           aggga----- Dog                             aggga-----                                 \*\*  \*        CSCS: 1.1549869381519566   - Pde6h                -356   -346  -  CTCTCTCTCT     - Mouse                           agagagagag Rat                             agaggaa--- Human                           aaaggga--- Dog                             aaaggga---                                 \* \*\*  \*      CSCS: 0.5501228260085576   - Pde6h                -358   -348  -  CTCTCTCTCT     - Mouse                           agagagagag Rat                             caagaggaa- Human                           agaaaggga- Dog                             agaaaggga-                                   \* \*\*  \*    CSCS: -0.05474128613484147   - Pde6h                -360   -350  -  CTCTCTCTCT     - Mouse                           agagagagag Rat                             agcaagagga Human                           agagaaaggg Dog                             agagaaaggg                                 \*\*  \* \*\*     CSCS: -0.5083893702423908   - Smug1                -240   -230  -  CCCTCTCTCA     - Mouse                           c---cctctctca Rat                             c-----tctcttt                                 \*\*\*\*  \*\*\*\*\*     CSCS: 0.41290629654072925   - Gngt2                -380   -370  +  GCCTCTCTGT     - Mouse                           gcct----ctctgt Rat                             gcct----ctctgt Human                           gtct----ctctgt Opossum                         gact----ctcag-                                 \* \*\*\*\*\*\*\*\*\* \*    CSCS: -0.453651687493905   - ENSMUSG00000024124   -271   -261  +  TCCTCTCTCA   - Arr3                 -332   -322  +  TTCTCTCTCT     - Mouse                           ttctctctct Rat                             ttctccctct                                 \*\*\*\*\* \*\*\*\*   CSCS: -0.5612471503419484   - ID: Opn1sw\_93\_103\_5     R|C/ N: (4/5)     Z: 3.8964052    Consensus:                           GAGAGAGAGAG   - Pde6h                -299   -288  +  GAGAGAGAGAA     - Mouse                           gagagagaga-a Rat                             taatggagaa-- Human                           gaataaaaaa-a Dog                             gaatggagga--                                  \*       \*\*    CSCS: 0.9095667372124778   - Pde6h                -301   -290  +  GAGAGAGAGAG     - Mouse                           gagagagagag Rat                             -gtaatggaga Human                           -agaataaaaa Dog                             -ggaatggagg                                    \*          CSCS: 1.3292510586274964   - Pde6h                -303   -292  +  GAGAGAGAGAG     - Mouse                           gagagagagag Rat                             tg-gtaatgga Human                           ta-agaataaa Dog                             tg-ggaatgga                                      \*        CSCS: 1.4691458324325026   - Pde6h                -305   -294  +  GAGAGAGAGAG     - Mouse                           gagagagagag Rat                             attg-gtaatg Human                           ggta-agaata Dog                             agtg-ggaatg                                        \*      CSCS: 1.4691458324325026   - Pde6h                -307   -296  +  GAGAGAGAGAG     - Mouse                           gagagagagag Rat                             aaattg-gtaa Human                           aaggta-agaa Dog                             acagtg-ggaa                                          \*    CSCS: 1.4691458324325026   - Pde6h                -309   -298  +  GAGAGAGAGAG     - Mouse                           gagagagagag Rat                             aaaaattg-gt Human                           taaaggta-ag Dog                             taacagtg-gg                                  \*            CSCS: 1.4691458324325026   - Pde6h                -311   -300  +  GAGAGAGAGAG     - Mouse                           gagagagagag Rat                             aaaaaaattg- Human                           tctaaaggta- Dog                             actaacagtg-                                    \*          CSCS: 1.7489353800425154   - Pde6h                -313   -302  +  GAGAGAGAGAG     - Mouse                           gagagagagag Rat                             caaaaaaaatt Human                           aatctaaaggt Dog                             gtactaacagt                                      \*        CSCS: 1.4691458324325026   - Pde6h                -315   -304  +  GAGAGAGAGAG     - Mouse                           gagagagagag Rat                             agcaaaaaaaa Human                           gaaatctaaag Dog                             aagtactaaca                                        \*      CSCS: 1.0494615110174839   - Pde6h                -317   -306  +  GAGAGAGAGAG     - Mouse                           gagagagagag Rat                             aaagcaaaaaa Human                           ttgaaatctaa Dog                             gcaagtactaa                                          \*    CSCS: 1.1893562848224903   - Pde6h                -319   -308  +  GAGAGAGAGAG     - Mouse                           gagagagagag Rat                             taaaagcaaaa Human                           tattgaaatct Dog                             tagcaagtact                                  \*            CSCS: 1.1893562848224903   - Pde6h                -321   -310  +  GAGAGAGAGAG     - Mouse                           gagagagagag Rat                             agtaaaagcaa Human                           ggtattgaaat Dog                             ggtagcaagta                                    \*          CSCS: 1.0494615110174839   - Pde6h                -323   -312  +  GAGAGAGAGAG     - Mouse                           g--------- Rat                             tt-------- Human                           g--------- Dog                             ag--------                                   \*\*\*\*\*\*\*\*\*\*\*\*\*\*\*\*\*\*\*\*\*\*\*\*\*\*\*\*\*\*    \*        CSCS: 1.1893562848224903   - Pde6h                -325   -314  +  GAGAGAGAGAG     - Mouse                           gag------- Rat                             gatt------ Human                           tgg------- Dog                             tgag------                                     \*\*\*\*\*\*\*\*\*\*\*\*\*\*\*\*\*\*\*\*\*\*\*\*\*\*\*\*\*\*    \*      CSCS: 1.3292510586274964   - Pde6h                -327   -316  +  GAGAGAGAGAG     - Mouse                           ga-------- Rat                             ta-------- Human                           aa-------- Dog                             aa--------                                  \*\*\*\*\*\*\*\*\*\*\*\*\*\*\*\*\*\*\*\*    \*\*\*\*\*\*\*\*\*\*\*\*\*\*\*\*\*\*\*\*\*\*\*\*\*\*\*\*\*\*    \*    CSCS: 1.1893562848224903   - Pde6h                -329   -318  +  GAGAGAGAGAG     - Mouse                           gaga------ Rat                             aata------ Human                           gaaa------ Dog                             aaaa------                                  \* \*\*\*\*\*\*\*\*\*\*\*\*\*\*\*\*\*\*\*\*    \*\*\*\*\*\*\*\*\*\*\*\*\*\*\*\*\*\*\*\*\*\*\*\*\*\*\*\*\*\*       CSCS: 1.1893562848224903   - Pde6h                -331   -320  +  GAGAGAGAGAG     - Mouse                           gagaga---- Rat                             -aaata---- Human                           -agaaa---- Dog                             -aaaaa----                                  \* \* \*\*\*\*\*\*\*\*\*\*\*\*\*\*\*\*\*\*\*\*    \*\*\*\*\*\*\*\*\*\*\*\*\*\*\*\*\*\*\*\*\*\*\*\*\*\*\*\*\*\*     CSCS: 0.7696719634074717   - Pde6h                -333   -322  +  GAGAGAGAGAG     - Mouse                           gagagaga-- Rat                             ---aaata-- Human                           ---agaaa-- Dog                             ---aaaaa--                                    \* \* \*\*\*\*\*\*\*\*\*\*\*\*\*\*\*\*\*\*\*\*    \*\*\*\*\*\*\*\*\*\*\*\*\*\*\*\*\*\*\*\*\*\*\*\*\*\*\*\*\*\*   CSCS: 1.0494615110174839   - Pde6h                -335   -324  +  GAGAGAGAGAG     - Mouse                           gagagagaga-------------------g Rat                             -----aaata-------------------g Human                           -----agaaa-------------------t Dog                             -----aaaaa-------------------t                                      \* \* \*\*\*\*\*\*\*\*\*\*\*\*\*\*\*\*\*\*\*\*    CSCS: 1.3292510586274964   - Pde6h                -337   -326  +  GAGAGAGAGAG     - Mouse                           gagagagagag Rat                             -------aaat Human                           -------agaa Dog                             -------aaaa                                        \* \*    CSCS: 1.8888301538475216   - Pde6h                -339   -328  +  GAGAGAGAGAG     - Mouse                           gagagagagag Rat                             ---------aa Human                           ---------ag Dog                             ---------aa                                          \*    CSCS: 2.3085144752625397   - Pde6h                -341   -330  +  GAGAGAGAGAG     - Mouse                           gagagagagag Rat                             ----------- Human                           ----------- Dog                             ----------- CSCS: 2.8680935704825647   - Pde6h                -343   -332  +  GAGAGAGAGAG     - Mouse                           gagagagagag Rat                             ----------- Human                           ----------- Dog                             ----------- CSCS: 2.8680935704825647   - Pde6h                -345   -334  +  GAGAGAGAGAG     - Mouse                           gagagagagag Rat                             ----------- Human                           ----------- Dog                             ----------- CSCS: 2.8680935704825647   - Pde6h                -347   -336  +  GAGAGAGAGAG     - Mouse                           gagagagagag Rat                             ----------- Human                           ----------- Dog                             ----------- CSCS: 2.8680935704825647   - Pde6h                -349   -338  +  GAGAGAGAGAG     - Mouse                           gagagagagag Rat                             ----------- Human                           ----------- Dog                             ----------- CSCS: 2.8680935704825647   - Pde6h                -351   -340  +  GAGAGAGAGAG     - Mouse                           gagagagagag Rat                             aa--------- Human                           ga--------- Dog                             ga---------                                  \*            CSCS: 2.168619701457534   - Pde6h                -353   -342  +  GAGAGAGAGAG     - Mouse                           gagagagagag Rat                             ggaa------- Human                           ggga------- Dog                             ggga-------                                 \*  \*          CSCS: 1.7489353800425154   - Pde6h                -355   -344  +  GAGAGAGAGAG     - Mouse                           gagagagagag Rat                             gaggaa----- Human                           aaggga----- Dog                             aaggga-----                                  \*\*  \*        CSCS: 1.1893562848224903   - Pde6h                -357   -346  +  GAGAGAGAGAG     - Mouse                           gagagagagag Rat                             aagaggaa--- Human                           gaaaggga--- Dog                             gaaaggga---                                  \* \*\*  \*      CSCS: 0.48988241579745917   - Pde6h                -359   -348  +  GAGAGAGAGAG     - Mouse                           gagagagagag Rat                             gcaagaggaa- Human                           gagaaaggga- Dog                             gagaaaggga-                                 \*  \* \*\*  \*    CSCS: -0.20959145322757197   - Pde6h                -361   -350  +  GAGAGAGAGAG     - Mouse                           gagagagagag Rat                             aagcaagagga Human                           cagagaaaggg Dog                             cagagaaaggg                                  \*\*  \* \*\*     CSCS: -0.20959145322757197   - Smug1                -241   -230  +  CTGAGAGAGGG     - Mouse                           c---cctctctcag Rat                             c-----tctctttg                                 \*\*\*\*  \*\*\*\*\*  \*   CSCS: 0.2362260910343898   - Gngt2                -380   -369  -  TACAGAGAGGC     - Mouse                           gcct----ctctgta Rat                             gcct----ctctgtc Human                           gtct----ctctgtc Opossum                         gact----ctcag-c                                 \* \*\*\*\*\*\*\*\*\* \*     CSCS: 0.43424587381862634   - ENSMUSG00000024124   -271   -260  -  ATGAGAGAGGA   - Arr3                 -332   -321  -  AAGAGAGAGAA     - Mouse                           ttctctctctt Rat                             ttctccctctt                                 \*\*\*\*\* \*\*\*\*\*   CSCS: -0.6171432413279778   - ID: Opn1mw\_358\_366\_3     R|C/ N: (4/5)     Z: 3.8964052    Consensus:                           MSCATGCTS   - ENSMUSG00000031471   -265   -256  -  CCCATGCTG   - Pde6c                -300   -291  +  CCCATGCTC     - Mouse                           cccatgctc Rat                             cccatactc Human                           cagatgttc Dog                             caaatactc                                 \*  \*\*  \*\*   CSCS: -0.7754382665230707   - Gnb3                 -226   -217  -  ACCATGCTT     - Mouse                           acca---tgctt Rat                             gcca---tgctt Human                           actg---tgctc Dog                             attt---tgtct Opossum                         agtaagtggctc                                         \*      CSCS: 0.48311708960942257   - cnga3                -311   -302  -  TGCATGCTG     - Mouse                           ca------gcatgca Rat                             --------ccaggca Human                           --------gaaggca Dog                             agatcg--gcgggcc                                       \*\*    \*\*    CSCS: 0.3582541757537296   - cngb3                -226   -217  -  ATCATGCTG     - Mouse                           cagcatgat Rat                             cagcatgat Human                           ttgtgtgat Dog                             caatgtgat Opossum                         gagcaatgt                                         \*   CSCS: 0.42769946138415116   - ID: Pde6g\_115\_125\_6     R|C/ N: (4/5)     Z: 3.8964052    Consensus:                           WGGAAAATMRA   - Smug1                -198   -187  +  TGGAAAATGGA     - Mouse                           tccattttcca Rat                             tccctttctca                                 \*\*\* \*\*\*  \*\*   CSCS: -0.29528261379298765   - Arr3                 -158   -147  -  TGGAAAACAGA     - Mouse                           tctg-ttttcca Rat                             tctg-ttttcca Human                           tctg-ctttcca Dog                             tctg-ctttcta Opossum                         gctc-ctttcca                                  \*\* \* \*\*\*\* \*   CSCS: -0.9364312985766245   - Gngt2                -190   -179  -  TGGAAAATCCA     - Mouse                           tggattttcca Rat                             tggattttcca Human                           tggattttcca Dog                             tggattttcca Opossum                         tggtttttcca                                 \*\*\* \*\*\*\*\*\*\*   CSCS: -1.5393446392147063   - cngb3                -248   -237  +  AGGAAAATAAC     - Mouse                           aggaaaataa Rat                             aggaaggaaa Human                           ggaaaaaaaa Dog                             --aaagaaaa Opossum                         ---------- CSCS: 2.5396291754178044   - ENSMUSG00000035540   -110    -99  -  CCAAAAATAGA |

Page by: Charles Danko & Maochun Qin; SUNY Upstate Medical University.
