# Supplementary material for: Bioinformatic identification of novel putative photoreceptor specific cis-elements
Source: BMC Bioinformatics. 2007 Oct 22;8:407. doi: 10.1186/1471-2105-8-407 (PMC2225425; doi:10.1186/1471-2105-8-407)
Supplement: Additional file 1 — Explanation of Supplementary Data. Detailed information on reading HTML formatted supplementary data. [file 1471-2105-8-407-S1.ZIP › EN2.html]

cis-Browser 

Predictions via cis-Browser

|  |
| --- |
| - ID: Pde6a\_841\_848\_14     R|C/ N: (4/6)     Z: 3.989411    Consensus:                           YYTTAAKW   - ENSMUSG00000043760    -80    -72  +  CGTTAATT   - ENSMUSG00000043760    -73    -65  -  GTTTAATA   - cngb3                 -93    -85  +  TTTTAAGT     - Mouse                           tttt Rat                             tttt Human                           tttt Dog                             tttt Opossum                         ctta                                 \*\*   Mouse                           agt Rat                             agc Human                           agc Dog                             agc Opossum                         aat Chicken                         agc                                 \*    CSCS: 0.5051564927011539   - Gnat2                 -72    -64  +  TTTTAATT     - Mouse                           t-tttaatt Rat                             t-tttaatt Human                           t-cttaatt Dog                             t-cttaatt Opossum                         tccttaatt                                 \*  \*\*\*\*\*\*  CSCS: -1.4915936110776333   - cnga3                -116   -108  -  TTTTAAGG     - Mouse                           ccttaaaa Rat                             ccttaaaa Human                           ctctgcaa Dog                             ccttgcaa                                 \*  \*  \*\*  CSCS: -0.17150363449065706   - ENSMUSG00000024906   -110   -102  +  TTTTAACA   - Opn1mw                -99    -91  -  TCTTAATT     - Mouse                           aattaaga Rat                             aattaaga Human                           aattaaga Dog                             aattaaga Opossum                         aattaaga X.tropicalis                    aattaaga                                 \*\*\*\*\*\*\*\*  CSCS: -1.8253065051916892   - ID: Elovl2\_230\_237\_9     R|C/ N: (4/6)     Z: 3.989411    Consensus:                           NNAATTMW   - ENSMUSG00000043760    -78    -70  +  TTAATTAT   - ENSMUSG00000043760   -157   -149  +  TTAATTCT   - Opn1mw               -101    -93  +  CCAATTAA     - Mouse                           ccaattaa Rat                             ccaattaa Human                           ccaattaa Dog                             ccaattaa Opossum                         tcaattaa X.tropicalis                    ccaattaa                                 \*\*\*\*\*\*\*  CSCS: -1.6669343231235867   - ENSMUSG00000025329    -34    -26  +  GAAATTAA   - cngb3                 -53    -45  +  GGAATTAA     - Mouse                           ggaattaa Rat                             ggaattaa Human                           ggaattaa Dog                             ggaattaa Opossum                         ggaattaa Chicken                         ggcatcaa                                 \*\* \*\* \*\*  CSCS: -1.3202457270951442   - Gnat2                 -70    -62  +  TTAATTGG     - Mouse                           ttaattgg Rat                             ttaattgg Human                           ttaattgg Dog                             ttaattgg Opossum                         ttaattgg                                 \*\*\*\*\*\*\*\*  CSCS: -2.1062154657354775   - Arr3                 -130   -122  +  CTAATTAT     - Mouse                           ctaattat-- Rat                             ctaattat-- Human                           ctatttat-- Dog                             ctatttataa Opossum                         ctctttgg--                                 \*\*  \*\*      CSCS: -0.32127293892259806 |

Page by: Charles Danko & Maochun Qin; SUNY Upstate Medical University.
