# Supplementary material for: Bioinformatic identification of novel putative photoreceptor specific cis-elements
Source: BMC Bioinformatics. 2007 Oct 22;8:407. doi: 10.1186/1471-2105-8-407 (PMC2225425; doi:10.1186/1471-2105-8-407)
Supplement: Additional file 1 — Explanation of Supplementary Data. Detailed information on reading HTML formatted supplementary data. [file 1471-2105-8-407-S1.ZIP › IL6.html]

cis-Browser 

Predictions via cis-Browser

|  |
| --- |
| - ID: Pde6g\_294\_302\_8     R|C/ N: (4/7)     Z: 3.9808664    Consensus:                           STGGGATKW   - Pde6g                -103    -94  -  CTGGGATGC     - 1.2131147540983607              Ratio: Mouse                           ctgggatgc Human                           caagg-ttc Dog                             ggaggattt                                   \*\* \*     CSCS: 0.5094102651622908   - Rho                  -118   -109  +  GTGGGATTA     - 0.49074074074074076             Ratio: Mouse                           gt-g--ggatt Rat                             gt-g--ggatt Human                           gcgg--ggatt Dog                             gtgg--ggatt Opossum                         gcgg--ggatt X.tropicalis                    gctt--ggatt                                   \*   \*\*\*\*\*\*\*  NaN                             Ratio:                                 Mouse                                 Rat                                 Human                                 Dog                                 Opossum                                 X.tropicalis  CSCS: NaN   - ENSMUSG00000038797   -107    -98  +  CTGGGATCT   - ENSMUSG00000048439    -41    -32  -  ATGGGATTT   - Pde6a                 -70    -61  -  GAGGGATTA     - 0.33292483660130723             Ratio: Mouse                           taatc------cctc Rat                             taatcc-----cccc Human                           taatct-----ccca Dog                             taatct-----cctg Opossum                         caatcttgtaccctc                                   \*\*\*\*      \*\*     CSCS: -1.5962913817594404   - Gnat1                 -69    -60  -  GTGGGATTT     - 0.3619047619047619              Ratio: Mouse                           gtgggattt Rat                             gtgggattt Human                           gtgggattt Dog                             gtaggattt Opossum                         gcaggattt                                   \*  \*\*\*\*\*\*   CSCS: -1.2576149203369287   - ENSMUSG00000034278   -107    -98  -  CTGGGATGA   - ID: Opn1mw\_101\_108\_15     R|C/ N: (4/4)     Z: 4.941698    Consensus:                           NCTGGGAS   - Gnb1                   97    105  +  GCTGGGAC     - 1.705128205128205               Ratio: Mouse                           gctgggac Rat                             gctgggac Human                           gccgggag Dog                             --cgggag                                   \*\*\*\*    CSCS: 0.776249804453297   - Sag                   103    111  +  ACTGGGTG     - 1.2084407216494844              Ratio: Mouse                           actgggtg Rat                             -----tcg Human                           cctggttg Dog                             gctggctg                                   \*   CSCS: 0.42404493883721645   - Nrl                   123    131  -  TCTGGGAC     - 1.2638888888888888              Ratio: Mouse                           ccctcaag- Rat                             cacccacga Human                           cacccaggt Dog                             cacccaggt Opossum                         --cttaggt                                   \*  \* \*    CSCS: 0.454833432516318   - Pde6d                 119    127  +  CGTGGGAG     - 1.3217391304347827              Ratio: Mouse                           ctcccacg Rat                             ctcccacg Human                           ttctcact Dog                             ctcccacg Chicken                         gcgccggg                                   \*      CSCS: 0.7348869403459909   - ID: Gnb3\_560\_570\_2     R|C/ N: (5/8)     Z: 4.0735784    Consensus:                           SCTGGGAARNS   - ENSMUSG00000031957   -381   -370  +  GCTGGGAAGCC   - ENSMUSG00000024803   -433   -422  -  GCTGGGAAGTG   - ENSMUSG00000024803   -267   -256  -  ATCGGGAAGCC   - Rho                  -361   -350  +  CCTGGGAAGAG     - 0.9716494845360825              Ratio: Mouse                           cct--gggaagag Rat                             cct--gggaagag Human                           ctt--gtggggga Dog                             ctt--gtggggaa                                   \* \*\*\*\* \*  \*     CSCS: -0.07066170190808235   - Pde6d                -336   -325  +  GCTGGGAAATG     - 0.0                             Ratio: Mouse                           catttcccagc Rat                             catttcccagc Human                           catttcccagc Dog                             catttcccagc                                   \*\*\*\*\*\*\*\*\*\*\*   CSCS: -1.5102709612362777   - Nrl                  -420   -409  +  GCTGGGAACGC   - Gnat1                -365   -354  +  GCTGGGAAAAC     - 0.9883449883449884              Ratio: Mouse                           gttttcccagc Rat                             gcttgcccagc Human                           gcctgcccggc Dog                             c-ctgcccagc                                   \* \*\*\* \*\*   CSCS: -0.027383339736015515   - ENSMUSG00000066515   -221   -210  +  CCTGGGAAGAT   - Pde6b                -418   -407  -  CCTGGGAAGGT     - 0.5043988269794721              Ratio: Mouse                           accttcccagg- Rat                             accttcccagg- Human                           acctccccagc-                                   \*\*\*\* \*\*\*\*\* \*   CSCS: -0.8429313761596674 |

Page by: Charles Danko & Maochun Qin; SUNY Upstate Medical University.
