# Supplementary material for: Bioinformatic identification of novel putative photoreceptor specific cis-elements
Source: BMC Bioinformatics. 2007 Oct 22;8:407. doi: 10.1186/1471-2105-8-407 (PMC2225425; doi:10.1186/1471-2105-8-407)
Supplement: Additional file 1 — Explanation of Supplementary Data. Detailed information on reading HTML formatted supplementary data. [file 1471-2105-8-407-S1.ZIP › INR.html]

cis-Browser 

Predictions via cis-Browser

|  |
| --- |
| - ID: Opn1mw\_1260\_1268\_11     R|C/ N: (5/7)     Z: 5.2183123    Consensus:                           NGACAGTRW   - Gnat1                  38     47  -  GGACAGGGT     - 0.914030819140308               Ratio: Mouse                           ggacagggt Rat                             ggacagggt Human                           ggacagagt Dog                             ---caggat Opossum                         gggcagaaa X.tropicalis                    caagagag-  CSCS: -0.5814596514566847   - ENSMUSG00000007817    -12     -3  -  AGACAGTGA   - Nr2e3                 -31    -22  -  ACACAGTGT   - Sag                    53     62  -  CGACAGTGA     - 0.9765177548682703              Ratio: Mouse                           tcactgtcg Rat                             tcaccatct Human                           tcatcatct Dog                             tcacc-ccg                                   \*\*\*    \*    CSCS: -0.049364112697844145   - Pde6g                   0      9  +  GGCCAGTGT     - 1.3479052823315119              Ratio: Mouse                           acactggcc Human                           gtgccagcc Dog                             gtgccgg-c                                   \*  \* \*   CSCS: 0.8316013730427143   - Nrl                    61     70  +  TGACAGTGA     - 0.6049382716049382              Ratio: Mouse                           tc-actgtca Rat                             tc-accgtca Human                           tc-agtgcca Dog                             tc-agtgtca Opossum                         tc-aaagtta                                   \*\*\*\*  \*  \*   CSCS: -0.7067729670354492   - ENSMUSG00000032323     18     27  +  GGACAGTNN   - ID: Pde6g\_2020\_2028\_1     R|C/ N: (4/5)     Z: 5.0074553    Consensus:                           SMGGTCCMG   - Pde6g                  19     28  +  CAGGTCCAG     - 1.0783242258652095              Ratio: Mouse                           ctggacctg Human                           ctgggcctc Dog                             ccaggtatc                                   \*  \*   \*    CSCS: 0.1872191572818674   - Nr2e3                   0      9  -  TGGGTCCAG     - 1.011111111111111               Ratio: Mouse                           tgggtc-----cag Human                           tggcttga--ggag Opossum                         acaggcag--gaag X.tropicalis                    tatggtca--agag                                   \*\*  \*\*   CSCS: 0.046595013348603755   - Sag                    25     34  +  CAGGTCCCT     - 1.2694730813287514              Ratio: Mouse                           caggtccct Rat                             cagttccct Human                           cagcttgct Dog                             ---------  CSCS: 0.5664832932764784   - ENSMUSG00000036537    -35    -26  +  GCGGTCCAG   - Gnat1                 -47    -38  -  CAGGTCCTG     - 1.3269841269841272              Ratio: Mouse                           caggtcct--------g Rat                             caggccct--------g Human                           caatccct--------g Dog                             caccccct--------g Opossum                         ---ttcct--------g                                   \*\*\*\*\*\*\*\*\*\*\*\*   CSCS: 0.6444494367895707   - ID: Pde6c\_946\_954\_10     R|C/ N: (5/8)     Z: 4.7692127    Consensus:                           RTCTGAGGN   - Pde6a                  -2      7  -  ATCTGAGGT     - 0.7324346405228759              Ratio: Mouse                           acctca---g Rat                             aggtca---g Human                           agtcca---g Dog                             agccca---g Opossum                         agctcatcag                                   \*   \*\*   \*                           CSCS: -0.6402760900504796   - Gnat1                  12     21  +  GTCTGAGGA     - 0.9935117599351175              Ratio: Mouse                           tcctcagac Rat                             tcctcagac Human                           tcc-caggc Dog                             tccc--aac Opossum                         ttccctaat X.tropicalis                    tctctaaa-  CSCS: -0.043883747279750474   - ENSMUSG00000044469    -41    -32  -  ATCTGAGCC   - ENSMUSG00000047759     21     30  +  GGCTGAGGA   - Gnb1                   27     36  +  GTCTGAGGG     - 0.0                             Ratio: Mouse                           gtctgaggg Rat                             gtctgaggg Human                           gtctgaggg Dog                             gtctgaggg                                   \*\*\*\*\*\*\*\*\*   CSCS: -1.121702643971277   - Sag                    30     39  -  CTCTGAGGG     - 0.7812142038946162              Ratio: Mouse                           ccctcagag Rat                             ccctcagcg Human                           tgctcagaa Dog                             ----cagag                                   \*\*\*     CSCS: -0.4599290500140595   - ENSMUSG00000021650    -40    -31  -  CTCTGAGGT   - Cnga1                 -21    -12  -  ATCTGAGAA     - 0.42145593869731807             Ratio: Mouse                           atctgag--aa Rat                             atctgag--aa Human                           atctgag--ga Dog                             atctggggaga Opossum                         atctgat--ga                                   \*\*\*\*\*     \*   CSCS: -1.24188490118517   - ID: Pde6b\_1548\_1556\_12     R|C/ N: (5/9)     Z: 4.3428607    Consensus:                           CCTCAGANS   - Gnb1                   26     35  -  CCTCAGACG     - 0.0                             Ratio: Mouse                           cgtctgagg Rat                             cgtctgagg Human                           cgtctgagg Dog                             cgtctgagg                                   \*\*\*\*\*\*\*\*\*   CSCS: -1.121702643971277   - ENSMUSG00000062077    -10     -1  -  GCTCAGAAC   - Nr2e3                  29     38  +  CTTCAGAAG     - 0.7777777777777778              Ratio: Mouse                           cttctg-aag Human                           gtcccg-aag Opossum                         ctcctgcgag X.tropicalis                    tttctc-tta                                   \* \*         CSCS: -0.9319002669720784   - Sag                    31     40  +  CCTCAGAGC     - 0.5859106529209621              Ratio: Mouse                           cctcagagc Rat                             cctcagcgc Human                           gctcagaac Dog                             ---cagagc                                   \*\*\*  \*   CSCS: -0.8704939873302746   - ENSMUSG00000025329     -4      5  +  CATCAGAAG   - ENSMUSG00000027596    -13     -4  -  CCCCAGAAG   - Pde6a                  -1      8  +  CCTCAGATT     - 0.8656045751633987              Ratio: Mouse                           cctca---ga Rat                             ggtca---ga Human                           gtcca---ga Dog                             gccca---gg Opossum                         gctcatcaga                                   \*\*   \*                            CSCS: -0.32160432614749285   - Gnat1                  11     20  -  CCTCAGACG     - 1.192214111922141               Ratio: Mouse                           cctcagacg Rat                             cctcagaca Human                           cc-caggca Dog                             ccc--aaca Opossum                         tccctaata X.tropicalis                    ctctaaa--  CSCS: 1.300056013162585   - ENSMUSG00000021650    -39    -30  +  CCTCAGAGG   - ID: Pde6g\_1576\_1584\_9     R|C/ N: (6/14)     Z: 3.999445    Consensus:                           RGGCACAGN   - Pde6g                  38     47  -  AGCCACAGG     - 1.7522768670309654              Ratio: Mouse                           agccacagg Human                           ggct----g Dog                             tcac----g                                   \*   CSCS: 1.798174696683984   - Sag                    64     73  +  AAGCACAGG     - 0.7812142038946162              Ratio: Mouse                           aagcacagg Rat                             aagcacagg Human                           gagcataga Dog                             ggg-acgga                                   \* \*  \*    CSCS: -0.4599290500140595   - Sag                   -58    -49  +  GGGCACAGA     - 0.0                             Ratio: Mouse                           g Rat                             g Human                           g Dog                             g                                   \*  0.3766568483063328              Ratio: Mouse                           gca-------caga Rat                             gcatct---ccaca Human                           gcatcctcgctaga Dog                             gcaccctgtccagg                                   \*\*\*        \*     CSCS: -1.2235982469627582   - ENSMUSG00000027995     35     44  -  AGCCACAGG   - ENSMUSG00000052353     86     95  -  CGGCACAGT   - Nr2e3                  84     93  +  AGGCACAGA     - 0.6597222222222222              Ratio: Mouse                           tctgtgc--- Human                           cctgaact-- Opossum                         tctgtcctag                                   \*\*\*  \*           \*\*\*\*\*\*\*\*\*\*\*\*\*\*\*\*\*\*\*\*\*\*\*\*\*\*\*\*\*\*\*\*\*\*\*     CSCS: -1.0392181363089315   - Nrl                    43     52  -  AGGCACAGC     - 0.43209876543209874             Ratio: Mouse                           aggcacagc Rat                             ctgcacagc Human                           aggcacagc Dog                             aggcacagc Opossum                         aggtaccac                                   \* \*\*  \*   CSCS: -1.0159861401134582   - Nrl                   -36    -27  -  GGGCACAGA     - 0.25925925925925924             Ratio: Mouse                           gggcacaga Rat                             gggcacaga Human                           gggcacaga Dog                             gggcaccaa Opossum                         ggacacaga                                   \*\* \*\*\*  \*   CSCS: -1.3251993131914674   - ENSMUSG00000027481      7     16  -  GGGCACAGC   - Gnat1                 -42    -33  -  CTGCACAGG     - 1.6888888888888889              Ratio: Mouse                           ctgcacagg Rat                             ctgcacagg Human                           ctgtgcaat Dog                             ctgtgcacc Opossum                         ctcc----t                                   \*\*          CSCS: 1.3577235707120077   - ENSMUSG00000021650    -77    -68  +  AGGCACAGA   - ENSMUSG00000020327    -10     -1  +  CGGCACAGG   - ENSMUSG00000026983     77     86  -  AGGCACACT   - ENSMUSG00000032059    -39    -30  +  TGGCACAGT   - ENSMUSG00000020868    -11     -2  -  GCGCACAGG   - Pde6b                 -21    -12  +  GGGCACAGC     - 1.1766586730615507              Ratio: Mouse                           gggcaca-gc Rat                             ggcacagcag Human                           gggacag-gc Dog                             g--acag-ac Opossum                         gggactg-tg                                   \*            CSCS: 0.8927084935697425   - ID: Nr2e3\_2084\_2094\_1     R|C/ N: (4/7)     Z: 3.9808664    Consensus:                           MAGGCACAGAN   - Nr2e3                  83     94  +  CAGGCACAGAC     - 0.7196969696969697              Ratio: Mouse                           gtctgtgc-- Human                           tcctgaact- Opossum                         ctctgtccta                                   \*\*\*  \*           \*\*\*\*\*\*\*\*\*\*\*\*\*\*\*\*\*\*\*\*\*\*\*\*\*\*\*\*\*\*\*\*\*\*\*  \*   CSCS: -0.9058848980233705   - ENSMUSG00000021650    -78    -67  +  CAGGCACAGAG   - Sag                   -59    -48  +  TGGGCACAGAT     - 0.5402930402930404              Ratio: Mouse                           tg Rat                             tg Human                           tg Dog                             -g                                   \*  0.4394329896907216              Ratio: Mouse                           gca-------cagat-- Rat                             gcatct---ccacagat Human                           gcatcctcgctagat-c Dog                             gcaccctgtccaggt-c                                   \*\*\*        \*        CSCS: -1.1403990627152352   - Rho                   -29    -18  -  CAGGCACTGAC     - 0.9527272727272728              Ratio: Mouse                           g-------tcagtgcctg Rat                             g-------tcagtgcctg Human                           g-------tcagaaccca Dog                             g-------tcagagcctg Opossum                         g-------ccacagttcc X.tropicalis                    g-------tctcagtgta                                   \*\*\*\*\*\*\*\* \*           CSCS: -0.127975798861171   - ENSMUSG00000026983     76     87  -  CAGGCACACTC   - ENSMUSG00000043760    144    155  +  CAGGCACAAAA   - Nrl                    42     53  -  AAGGCACAGCT     - 0.5656565656565656              Ratio: Mouse                           aaggcacagct Rat                             actgcacagct Human                           caggcacagct Dog                             caggcacagct Opossum                         caggtaccact                                   \* \*\*  \*\*   CSCS: -0.8397313136888972   - Nrl                   -37    -26  -  TGGGCACAGAG     - 0.3535353535353535              Ratio: Mouse                           tgggcacagag Rat                             tgggcacagag Human                           tgggcacagag Dog                             agggcaccaag Opossum                         tggacacagaa                                   \*\* \*\*\*  \*    CSCS: -1.249832652932312   - ID: cngb3\_1726\_1736\_4     R|C/ N: (4/5)     Z: 4.283107    Consensus:                           MARGCACAGRW   - Gnat1                 -43    -32  -  CCTGCACAGGT     - 1.6779220779220778              Ratio: Mouse                           cctgcacaggt Rat                             cctgcacaggc Human                           cctgtgcaatc Dog                             cctgtgcaccc Opossum                         cctcc----tt                                   \*\*\*           CSCS: 1.5691842441682395   - ENSMUSG00000030669    -24    -13  +  GAGGCACAGGA   - Sag                    63     74  +  AAAGCACAGGT     - 0.7989690721649484              Ratio: Mouse                           aaagcacaggt Rat                             aaagcacaggt Human                           agagcatagag Dog                             aggg-acggag                                   \*  \* \*  \*     CSCS: -0.45063350407481917   - Nr2e3                  83     94  +  CAGGCACAGAC     - 0.7196969696969697              Ratio: Mouse                           gtctgtgc-- Human                           tcctgaact- Opossum                         ctctgtccta                                   \*\*\*  \*           \*\*\*\*\*\*\*\*\*\*\*\*\*\*\*\*\*\*\*\*\*\*\*\*\*\*\*\*\*\*\*\*\*\*\*  \*   CSCS: -0.9058848980233705   - Nrl                    42     53  -  AAGGCACAGCT     - 0.5656565656565656              Ratio: Mouse                           aaggcacagct Rat                             actgcacagct Human                           caggcacagct Dog                             caggcacagct Opossum                         caggtaccact                                   \* \*\*  \*\*   CSCS: -0.8397313136888972   - ID: Opn1mw\_1260\_1268\_9     R|C/ N: (5/8)     Z: 4.0735784    Consensus:                           RGACAGTGW   - Gnat1                  38     47  -  GGACAGGGT     - 0.914030819140308               Ratio: Mouse                           ggacagggt Rat                             ggacagggt Human                           ggacagagt Dog                             ---caggat Opossum                         gggcagaaa X.tropicalis                    caagagag-  CSCS: -0.5814596514566847   - Nr2e3                 -31    -22  -  ACACAGTGT   - Sag                    53     62  -  CGACAGTGA     - 0.9765177548682703              Ratio: Mouse                           tcactgtcg Rat                             tcaccatct Human                           tcatcatct Dog                             tcacc-ccg                                   \*\*\*    \*    CSCS: -0.049364112697844145   - Pde6g                   0      9  +  GGCCAGTGT     - 1.3479052823315119              Ratio: Mouse                           acactggcc Human                           gtgccagcc Dog                             gtgccgg-c                                   \*  \* \*   CSCS: 0.8316013730427143   - ENSMUSG00000024233    104    113  -  AGACAGTGA   - Nrl                    61     70  +  TGACAGTGA     - 0.6049382716049382              Ratio: Mouse                           tc-actgtca Rat                             tc-accgtca Human                           tc-agtgcca Dog                             tc-agtgtca Opossum                         tc-aaagtta                                   \*\*\*\*  \*  \*   CSCS: -0.7067729670354492   - ENSMUSG00000049152      2     11  +  AGACAGTGC   - ENSMUSG00000031445    -22    -13  -  GGTCAGTGT |

Page by: Charles Danko & Maochun Qin; SUNY Upstate Medical University.
