# Supplementary material for: Bioinformatic identification of novel putative photoreceptor specific cis-elements
Source: BMC Bioinformatics. 2007 Oct 22;8:407. doi: 10.1186/1471-2105-8-407 (PMC2225425; doi:10.1186/1471-2105-8-407)
Supplement: Additional file 1 — Explanation of Supplementary Data. Detailed information on reading HTML formatted supplementary data. [file 1471-2105-8-407-S1.ZIP › NRE.html]

cis-Browser 

Predictions via cis-Browser

|  |
| --- |
| - ID: Rho\_1913\_1920\_1     R|C/ N: (5/10)     Z: 4.069864    Consensus:                           WATGCTGM   - Rho                   -88    -80  +  GATGCTGA     - 0.21833333333333332             Ratio: Mouse                           gatgctga Rat                             gatgctga Human                           gatgctga Dog                             ggtgctga Opossum                         ggtgctga X.tropicalis                    gttgctga                                   \* \*\*\*\*\*\*   CSCS: -1.86764296166387   - ENSMUSG00000029070    -96    -88  -  AATGCTGC   - Pde6g                -106    -98  -  GATGCTGG     - 0.9098360655737705              Ratio: Mouse                           gatgctgg Human                           g-ttcagg Dog                             gatttagg                                   \* \*   \*\*   CSCS: -0.20640603569244648   - ENSMUSG00000048439    -77    -69  -  TATGCTGA   - Pde6b                 -77    -69  +  TTTGCTGA     - 0.0                             Ratio: Mouse                           tttgctga Rat                             tttgctga Human                           tttgctga Dog                             tttgctga Opossum                         tttgctga                                   \*\*\*\*\*\*\*\*   CSCS: -1.6589117087720509   - Cnga1                 -98    -90  -  AATGCTGA     - 1.1041666666666667              Ratio: Mouse                           aatgctga Human                           -atgctat                                   \*\*\*\*\*     CSCS: 0.2048115228768415   - ENSMUSG00000069743    -93    -85  -  AATGCTGG   - Nr2e3                 -90    -82  +  AATGCTGC     - 1.1271186440677967              Ratio: Mouse                           gcagcatt Human                           gcagcatt Opossum                         gaagaatt Chicken                         gaagaatt X.tropicalis                    gaagaatt  CSCS: 0.23856680818984288   - ENSMUSG00000034528   -115   -107  +  TATGCTGC   - ENSMUSG00000044469   -112   -104  +  TTTGCTGA |

Page by: Charles Danko & Maochun Qin; SUNY Upstate Medical University.
