# Supplementary material for: Bioinformatic identification of novel putative photoreceptor specific cis-elements
Source: BMC Bioinformatics. 2007 Oct 22;8:407. doi: 10.1186/1471-2105-8-407 (PMC2225425; doi:10.1186/1471-2105-8-407)
Supplement: Additional file 1 — Explanation of Supplementary Data. Detailed information on reading HTML formatted supplementary data. [file 1471-2105-8-407-S1.ZIP › r.NS.html]

cis-Browser 

Predictions via cis-Browser

|  |
| --- |
| - ID: Gnb1\_1686\_1698     R|C/ N: (4/4)     Z: 4.5394983    Consensus:                           RCCTTTCRGCCCT   - Gnb1                 -315   -302  +  GCCTTTCGGCCCT     - Mouse                           gcctttcggccct Rat                             gcttttccgctct Human                           gcccaccggcccc                                 \*\*    \* \*\* \*    CSCS: 0.024319080305123347   - Pde6g                -375   -362  -  GCCTTTCAGCCCT     - Mouse                           gcctttcagccct Dog                             caccttctctctc                                   \* \*\*\*   \*     CSCS: 0.6645664032364412   - Rho                  -289   -276  -  ACCTTTGGGCCCT     - Mouse                           agggcccaaa Rat                             agggcccaaa Human                           agaggcccat Dog                             -ggagccaga                                  \*   \*\*   \*\*\*\*\*\*\*\*\*\*\*\*\*\*\*\*\* \*\*\*\*\*\*    CSCS: 0.3537508513127135   - Pde6a                -272   -259  -  TCCTTTCTGCCCT     - Mouse                           agggcagaaagga Rat                             agggcagcgagga Human                           aggtcagagaaga Dog                             agggcagagaaaa                                 \*\*\* \*\*\*  \*  \*   CSCS: -1.6377554542824095   - ID: Pde6a\_1684\_1696     R|C/ N: (4/4)     Z: 4.5394983    Consensus:                           AGTCAWTGTCAGG   - Pde6a                -317   -304  +  AGTCATTGTCAGG     - Mouse                           agtcattgtcagg Rat                             ------tgtcagt Human                           agtaaatgtcagt Dog                             agcataggt-agg                                        \*\* \*\*    CSCS: -0.17694545855345192   - Rho                  -403   -390  +  AGCCATTGTCAGG     - Mouse                           agccattgtcagg Rat                             agctggtgtcagg Human                           a------------ Dog                             ggctgggctcaga CSCS: 1.7115269091125789   - Cnga1               -1747  -1734  +  GGTCAATGTCAGG     - Mouse                           cctgacattgacc Rat                             cctgatgttgacc                                 \*\*\*\*\*  \*\*\*\*\*\*   CSCS: -0.01923322843080425   - Nrl                    59     72  -  AGTCACTGTCAGA     - Mouse                           agtc-actgtcaga Rat                             agtc-accgtcaga Human                           ggtc-agtgccaga Dog                             ggtc-agtgtcaga Opossum                         agtc-aaagttata                                  \*\*\*\*\*  \*  \* \*   CSCS: -0.8421613845489426   - ID: Pde6a\_1684\_1695     R|C/ N: (5/8)     Z: 3.9451532    Consensus:                           RGWCATTGTCAR   - Pde6a                -317   -305  +  AGTCATTGTCAG     - Mouse                           agtcattgtcag Rat                             ------tgtcag Human                           agtaaatgtcag Dog                             agcataggt-ag                                        \*\* \*\*   CSCS: -0.32283800342162805   - Rho                  -403   -391  +  AGCCATTGTCAG     - Mouse                           agccattgtcag Rat                             agctggtgtcag Human                           a----------- Dog                             ggctgggctcag CSCS: 1.5939653717835411   - Rho                  -327   -315  +  AGACATTGTCAA     - Mouse                           a--------- Rat                             a--------- Human                           a--------- Dog                             actgagaata                                 \*                \*\*\*\*\*\*      \*     \*\*       \*\*    \*  \*   CSCS: -1.1147809136225386   - Nrl                    60     72  -  AGTCACTGTCAG     - Mouse                           agtc-actgtcag Rat                             agtc-accgtcag Human                           ggtc-agtgccag Dog                             ggtc-agtgtcag Opossum                         agtc-aaagttat                                  \*\*\*\*\*  \*  \*    CSCS: -0.7085400761300135   - ENSMUSG00000037295   -404   -392  +  GATCATTGTCAA   - Gnb1                 -501   -489  +  AGTCATTGTGGC     - Mouse                           agtc------ Rat                             agtc------ Human                           aggcccaggc                                 \*\* \*                       \*\*                        \*\*\*    CSCS: -0.7843874841835871   - ENSMUSG00000063838  -1519  -1507  +  AGACATTGTCAC   - ENSMUSG00000062077  -1215  -1203  -  TGTCATTCTCAG   - Cnga1               -1747  -1735  +  GGTCAATGTCAG     - Mouse                           ctgacattgacc Rat                             ctgatgttgacc                                 \*\*\*\*  \*\*\*\*\*\*   CSCS: 0.09976870496053221   - ID: Pde6g\_986\_993\_10     R|C/ N: (4/4)     Z: 5.702139    Consensus:                           TCATACWR   - Pde6g                -130   -122  -  TCATACAG     - Mouse                           tc---atacag Human                           ctcaggtgctg Dog                             cccgggtgcag                                       \* \* \*   CSCS: 0.4878688116366915   - Pde6b                -129   -121  -  TCATACCA     - Mouse                           t---ggta Rat                             t---ggta Human                           tgtaggag                                 \*   \*\*     Mouse                           ga Rat                             ga Human                           ga Dog                             ga Opossum                         -- CSCS: 0.4648405621425595   - Rho                  -105    -97  -  TCATACTA     - Mouse                           tagtatga-------- Rat                             tagtgtgat------- Human                           gattatgaacaccccc Dog                             gattaataacgccccc Opossum                         -----gtcatgccctg X.tropicalis                    --ttattagtgcgcta CSCS: 0.9145742719164223   - Cnga1                -132   -124  +  GCATACTT     - Mouse                           aagtatgc Human                           --gtgttc                                   \*\* \* \*   CSCS: 0.9284789037083477   - ID: Opn1mw\_1260\_1268\_11     R|C/ N: (5/7)     Z: 5.2183123    Consensus:                           NGACAGTRW   - Gnat1                  38     47  -  GGACAGGGT     - Mouse                           ggacagggt Rat                             ggacagggt Human                           ggacagagt Dog                             ---caggat Opossum                         gggcagaaa X.tropicalis                    caagagag- CSCS: -0.5814596514566847   - ENSMUSG00000007817    -12     -3  -  AGACAGTGA   - Nr2e3                 -31    -22  -  ACACAGTGT   - Sag                    53     62  -  CGACAGTGA     - Mouse                           tcactgtcg Rat                             tcaccatct Human                           tcatcatct Dog                             tcacc-ccg                                 \*\*\*    \*    CSCS: -0.049364112697844145   - Pde6g                   0      9  +  GGCCAGTGT     - Mouse                           acactggcc Human                           gtgccagcc Dog                             gtgccgg-c                                    \*  \* \*   CSCS: 0.8316013730427143   - Nrl                    61     70  +  TGACAGTGA     - Mouse                           tc-actgtca Rat                             tc-accgtca Human                           tc-agtgcca Dog                             tc-agtgtca Opossum                         tc-aaagtta                                 \*\*\*\*  \*  \*   CSCS: -0.7067729670354492   - ENSMUSG00000032323     18     27  +  GGACAGTNN   - ID: Rho\_1897\_1904\_1     R|C/ N: (4/5)     Z: 5.0074553    Consensus:                           RGTATGAY   - Rho                  -104    -96  +  AGTATGAT     - Mouse                           agtatga--------t Rat                             agtgtgat-------- Human                           attatgaacaccccca Dog                             attaataacgccccca Opossum                         ----gtcatgccctga X.tropicalis                    -ttattagtgcgctaa CSCS: 1.0884628490151906   - Pde6g                -129   -121  +  TGTATGAC     - Mouse                           gtc---ataca Human                           gctcaggtgct Dog                             gcccgggtgca                                 \*      \* \*    CSCS: 0.4878688116366915   - Pde6b                -128   -120  +  GGTATGAG     - Mouse                           ggta Rat                             ggta Human                           ggag                                 \*\*     Mouse                           gag Rat                             gag Human                           gag Dog                             gag Opossum                         --- CSCS: 0.5478501247329967   - ENSMUSG00000026799   -164   -156  -  GTTATGAT   - Cnga1                -133   -125  -  AGTATGCC     - Mouse                           agtatgcc Human                           -gtgttcc                                  \*\* \* \*\*   CSCS: 0.2048115228768415   - ID: Gnb1\_1687\_1698\_1     R|C/ N: (4/5)     Z: 5.0074553    Consensus:                           CCTTTCRGCCCT   - Gnb1                 -314   -302  +  CCTTTCGGCCCT     - Mouse                           cctttcggccct Rat                             cttttccgctct Human                           cccaccggcccc                                 \*    \* \*\* \*    CSCS: 0.20019931604685723   - Rho                  -289   -277  -  CCTTTGGGCCCT     - Mouse                           agggcccaaa Rat                             agggcccaaa Human                           agaggcccat Dog                             -ggagccaga                                  \*   \*\*   \*\*\*\*\*\*\*\*\*\*\*\*\*\*\*\*\* \*\*\*\*\*\*   CSCS: 0.2659543331421266   - Pde6a                -272   -260  -  CCTTTCTGCCCT     - Mouse                           agggcagaaagg Rat                             agggcagcgagg Human                           aggtcagagaag Dog                             agggcagagaaa                                 \*\*\* \*\*\*  \*     CSCS: -1.487115483846437   - Pde6g                -375   -363  -  CCTTTCAGCCCT     - Mouse                           cctttcagccct Dog                             accttctctctc                                  \* \*\*\*   \*     CSCS: 0.4469156094481655   - ENSMUSG00000030643   -338   -326  +  CCTTTCGGCTCC   - ID: Pde6g\_2020\_2028\_1     R|C/ N: (4/5)     Z: 5.0074553    Consensus:                           SMGGTCCMG   - Pde6g                  19     28  +  CAGGTCCAG     - Mouse                           ctggacctg Human                           ctgggcctc Dog                             ccaggtatc                                 \*  \*   \*    CSCS: 0.1872191572818674   - Nr2e3                   0      9  -  TGGGTCCAG     - Mouse                           tgggtc-----cag Human                           tggcttga--ggag Opossum                         acaggcag--gaag X.tropicalis                    tatggtca--agag                                         \*\*  \*\*   CSCS: 0.046595013348603755   - Sag                    25     34  +  CAGGTCCCT     - Mouse                           caggtccct Rat                             cagttccct Human                           cagcttgct Dog                             --------- CSCS: 0.5664832932764784   - ENSMUSG00000036537    -35    -26  +  GCGGTCCAG   - Gnat1                 -47    -38  -  CAGGTCCTG     - Mouse                           caggtcct--------g Rat                             caggccct--------g Human                           caatccct--------g Dog                             caccccct--------g Opossum                         ---ttcct--------g                                      \*\*\*\*\*\*\*\*\*\*\*\*   CSCS: 0.6444494367895707   - ID: Pde6c\_946\_954\_10     R|C/ N: (5/8)     Z: 4.7692127    Consensus:                           RTCTGAGGN   - Pde6a                  -2      7  -  ATCTGAGGT     - Mouse                           acctca---g Rat                             aggtca---g Human                           agtcca---g Dog                             agccca---g Opossum                         agctcatcag                                 \*   \*\*   \*                           CSCS: -0.6402760900504796   - Gnat1                  12     21  +  GTCTGAGGA     - Mouse                           tcctcagac Rat                             tcctcagac Human                           tcc-caggc Dog                             tccc--aac Opossum                         ttccctaat X.tropicalis                    tctctaaa- CSCS: -0.043883747279750474   - ENSMUSG00000044469    -41    -32  -  ATCTGAGCC   - ENSMUSG00000047759     21     30  +  GGCTGAGGA   - Gnb1                   27     36  +  GTCTGAGGG     - Mouse                           gtctgaggg Rat                             gtctgaggg Human                           gtctgaggg Dog                             gtctgaggg                                 \*\*\*\*\*\*\*\*\*   CSCS: -1.121702643971277   - Sag                    30     39  -  CTCTGAGGG     - Mouse                           ccctcagag Rat                             ccctcagcg Human                           tgctcagaa Dog                             ----cagag                                     \*\*\*     CSCS: -0.4599290500140595   - ENSMUSG00000021650    -40    -31  -  CTCTGAGGT   - Cnga1                 -21    -12  -  ATCTGAGAA     - Mouse                           atctgag--aa Rat                             atctgag--aa Human                           atctgag--ga Dog                             atctggggaga Opossum                         atctgat--ga                                 \*\*\*\*\*     \*   CSCS: -1.24188490118517   - ID: Pde6g\_997\_1005\_3     R|C/ N: (5/8)     Z: 4.7692127    Consensus:                           WACATGGCR   - Pde6g                -224   -215  +  TACATGGAA     - Mouse                           ttccatgta Dog                             gcttgtctt                                      \* \*    CSCS: 1.4249485355701015   - Nr2e3                -325   -316  +  TACATGGCA     - Mouse                           tgccatgta Human                           tggcacaca                                 \*\* \*\*   \*   CSCS: 0.3429678304789139   - Gnb1                 -256   -247  +  CACATGGCA     - Mouse                           cacatggca Rat                             cacatggca Human                           cccacttca                                 \* \*\*   \*\*   CSCS: -0.5206041793777975   - ENSMUSG00000030643   -283   -274  +  AACATGGCG   - Nrl                  -283   -274  +  AACATGGCT     - Mouse                           agccatgtt Rat                             agccct--- Human                           agaaacatg Dog                             agctgcttt                                 \*\*          CSCS: 0.32002695077277715   - Rho                  -311   -302  -  CACATGGCT     - Mouse                           agccatg Rat                             agcaatg Human                           agctgtc Dog                             gtcagct                                   \*       Mouse                           g Rat                             g Human                           a Dog                             g CSCS: -0.4062831849960865   - ENSMUSG00000068391   -240   -231  -  ACCATGGCA   - ENSMUSG00000028427   -282   -273  -  TACATGCCA   - ID: cngb3\_1726\_1736\_4     R|C/ N: (4/6)     Z: 4.4265203    Consensus:                           MMRGCACAGRY   - ENSMUSG00000020327    -11      0  +  CCGGCACAGGT   - Gnat1                 -43    -32  -  CCTGCACAGGT     - Mouse                           cctgcacaggt Rat                             cctgcacaggc Human                           cctgtgcaatc Dog                             cctgtgcaccc Opossum                         cctcc----tt                                 \*\*\*           CSCS: 1.5691842441682395   - Sag                    63     74  +  AAAGCACAGGT     - Mouse                           aaagcacaggt Rat                             aaagcacaggt Human                           agagcatagag Dog                             aggg-acggag                                 \*  \* \*  \*     CSCS: -0.45063350407481917   - Nr2e3                  83     94  +  CAGGCACAGAC     - Mouse                           gtctgtgc-- Human                           tcctgaact- Opossum                         ctctgtccta                                   \*\*\*  \*           \*\*\*\*\*\*\*\*\*\*\*\*\*\*\*\*\*\*\*\*\*\*\*\*\*\*\*\*\*\*\*\*\*\*\*  \*   CSCS: -0.9058848980233705   - Nrl                    42     53  -  AAGGCACAGCT     - Mouse                           aaggcacagct Rat                             actgcacagct Human                           caggcacagct Dog                             caggcacagct Opossum                         caggtaccact                                    \* \*\*  \*\*   CSCS: -0.8397313136888972   - ENSMUSG00000021650    -78    -67  +  CAGGCACAGAG   - ID: Rho\_1541\_1549\_3     R|C/ N: (4/6)     Z: 4.4265203    Consensus:                           TGRGACTRR   - Rho                  -275   -266  -  TGCGACTGG     - Mouse                           ccagtc----gca Rat                             ccagtc----aca Human                           cctatt----tca Dog                             ccaatg----cca                                 \*\*  \* \*\*\*\* \*\*   CSCS: 0.6082287370157643   - Pde6d                -292   -283  -  TGAGACTAT     - Mouse                           tgagactat Rat                             tgagactat Human                           tgagacggc Dog                             tgaaatgac                                 \*\*\* \*       CSCS: 0.5316370721441761   - ENSMUSG00000044469   -305   -296  -  GGAGACTGA   - Cnga1                -243   -234  +  TGGGACTGG     - Mouse                           ccagtccca Rat                             ccaatccaa Human                           ccaggccac                                 \*\*\*  \*\*     CSCS: -0.6257531783326754   - Pde6b                -305   -296  -  TGAGACTAG     - Mouse                           ct-------------agtctca Rat                             ct-------------agtctca Human                           ctcctcaggggattagttctca                                 \*\*               \*\*\*\*\*   CSCS: -1.5514095556632541   - ENSMUSG00000034278   -273   -264  -  TGGGACTGG   - ID: Pde6b\_225\_235\_5     R|C/ N: (4/6)     Z: 4.4265203    Consensus:                           MSRCAGTGAMM   - Nrl                    61     72  +  TGACAGTGACT     - Mouse                           agtc-actgtca Rat                             agtc-accgtca Human                           ggtc-agtgcca Dog                             ggtc-agtgtca Opossum                         agtc-aaagtta                                  \*\*\*\*\*  \*  \*   CSCS: -0.7030308672744255   - ENSMUSG00000007817    -14     -3  -  AGACAGTGAAA   - ENSMUSG00000038797     77     88  -  AGACTGTGACC   - ENSMUSG00000038797     43     54  -  CCTCAGTGACC   - Rho                    30     41  -  AGACAGAGACC     - Mouse                           ggtctctgtct Rat                             gtcta------ Human                           gggtg------ Dog                             aggac------ Opossum                         gttag------ X.tropicalis                    gaagg------ CSCS: 0.7248270397519686   - Sag                    51     62  -  CGACAGTGAGC     - Mouse                           gctcactgtcg Rat                             gctcaccatct Human                           attcatcatct Dog                             gctcacc-ccg                                   \*\*\*    \*    CSCS: -0.09243764186150168   - Pde6g                 116    127  -  CTGCAGTGACC     - Mouse                           ccaggaccagg Human                           ----------- CSCS: 0.7039377035587134   - ID: Pde6d\_1613\_1623\_1     R|C/ N: (4/6)     Z: 4.4265203    Consensus:                           WRWAGTCCCTG   - Pde6d                -388   -377  +  TGTAGTCCCTG     - Mouse                           c-agggactaca Rat                             --aggaactaca Human                           c-ggggactaca Dog                             c-ggggactaca                                  \* \*\* \*\*\*\*\*\*   CSCS: -0.5341812269959745   - Pde6g                -414   -403  -  TGTAGTCCCAT   - Nr2e3                -298   -287  +  TGAAGTCCCTG     - Mouse                           cagggac-------ttca Human                           ---ggac-------ttta                                    \*\*\*\*\*\*\*\*\*\*\*\*\* \*   CSCS: -1.1727281762162   - ENSMUSG00000029415   -306   -295  -  TGTAATCCCTG   - ENSMUSG00000055053   -268   -257  -  CTGAGTCCCTG   - Pde6b                -387   -376  -  AAAAGTCCCTG     - Mouse                           cagggactttt Rat                             cagggactttt Human                           ctggggccacc                                 \* \*\*\* \*       CSCS: 0.8728579338931468   - ID: Pde6g\_1626\_1637\_1     R|C/ N: (4/6)     Z: 4.4265203    Consensus:                           RGGGCWGAAARG   - Pde6g                -375   -363  +  AGGGCTGAAAGG     - Mouse                           cctttcagccct Dog                             accttctctctc                                  \* \*\*\*   \*     CSCS: 0.4469156094481655   - Pde6a                -272   -260  +  AGGGCAGAAAGG     - Mouse                           agggcagaaagg Rat                             agggcagcgagg Human                           aggtcagagaag Dog                             agggcagagaaa                                 \*\*\* \*\*\*  \*     CSCS: -1.487115483846437   - Gnb1                 -314   -302  -  AGGGCCGAAAGG     - Mouse                           cctttcggccct Rat                             cttttccgctct Human                           cccaccggcccc                                 \*    \* \*\* \*    CSCS: 0.20019931604685723   - ENSMUSG00000020154   -410   -398  +  GGGGCTGAAAAG   - Nrl                  -482   -470  +  GGGGCTGAAATG     - Mouse                           catttcagcc----------cc Rat                             tatttcagcc----------cc Human                           catttctacc----------tc Dog                             cacttctgtcc---------cc Opossum                         cccttcttctttccaattgatt                                    \*\*\*                   CSCS: -0.3230313862085451   - ENSMUSG00000037060   -253   -241  +  AGGCCTGAAAGA   - ID: Elovl2\_1581\_1591\_5     R|C/ N: (4/6)     Z: 4.4265203    Consensus:                           NYSTGTGCCMR   - Cnga1                -190   -179  +  GCGTGTGCCAA     - Mouse                           ttggcacacgc Human                           tttgcatatgc                                 \*\* \*\*\* \* \*\*   CSCS: -0.4785676575667903   - ENSMUSG00000000037    -79    -68  -  CTGTGTGCCCG   - Sag                   -59    -48  -  ATCTGTGCCCA     - Mouse                           tg Rat                             tg Human                           tg Dog                             -g                                  \*   Mouse                           gca-------cagat-- Rat                             gcatct---ccacagat Human                           gcatcctcgctagat-c Dog                             gcaccctgtccaggt-c                                 \*\*\*        \*        CSCS: -1.1403990627152352   - Pde6d                -277   -266  +  TAGTGTGCCCG     - Mouse                           cgggcacacta Rat                             cgggtacacta Human                           tgagcacacta Dog                             ggggcacacta                                  \* \* \*\*\*\*\*\*   CSCS: -0.5341812269959745   - Nrl                   -37    -26  +  CTCTGTGCCCA     - Mouse                           tgggcacagag Rat                             tgggcacagag Human                           tgggcacagag Dog                             agggcaccaag Opossum                         tggacacagaa                                  \*\* \*\*\*  \*    CSCS: -1.249832652932312   - ENSMUSG00000038496   -121   -110  -  CCGTGTGCAGT   - ID: Pde6b\_1548\_1556\_12     R|C/ N: (5/9)     Z: 4.3428607    Consensus:                           CCTCAGANS   - Gnb1                   26     35  -  CCTCAGACG     - Mouse                           cgtctgagg Rat                             cgtctgagg Human                           cgtctgagg Dog                             cgtctgagg                                 \*\*\*\*\*\*\*\*\*   CSCS: -1.121702643971277   - ENSMUSG00000062077    -10     -1  -  GCTCAGAAC   - Nr2e3                  29     38  +  CTTCAGAAG     - Mouse                           cttctg-aag Human                           gtcccg-aag Opossum                         ctcctgcgag X.tropicalis                    tttctc-tta                                  \* \*         CSCS: -0.9319002669720784   - Sag                    31     40  +  CCTCAGAGC     - Mouse                           cctcagagc Rat                             cctcagcgc Human                           gctcagaac Dog                             ---cagagc                                    \*\*\*  \*   CSCS: -0.8704939873302746   - ENSMUSG00000025329     -4      5  +  CATCAGAAG   - ENSMUSG00000027596    -13     -4  -  CCCCAGAAG   - Pde6a                  -1      8  +  CCTCAGATT     - Mouse                           cctca---ga Rat                             ggtca---ga Human                           gtcca---ga Dog                             gccca---gg Opossum                         gctcatcaga                                    \*\*   \*                            CSCS: -0.32160432614749285   - Gnat1                  11     20  -  CCTCAGACG     - Mouse                           cctcagacg Rat                             cctcagaca Human                           cc-caggca Dog                             ccc--aaca Opossum                         tccctaata X.tropicalis                    ctctaaa-- CSCS: 1.300056013162585   - ENSMUSG00000021650    -39    -30  +  CCTCAGAGG   - ID: Rho\_1913\_1920\_1     R|C/ N: (5/10)     Z: 4.069864    Consensus:                           WATGCTGM   - Rho                   -88    -80  +  GATGCTGA     - Mouse                           gatgctga Rat                             gatgctga Human                           gatgctga Dog                             ggtgctga Opossum                         ggtgctga X.tropicalis                    gttgctga                                 \* \*\*\*\*\*\*   CSCS: -1.86764296166387   - ENSMUSG00000029070    -96    -88  -  AATGCTGC   - Pde6g                -106    -98  -  GATGCTGG     - Mouse                           gatgctgg Human                           g-ttcagg Dog                             gatttagg                                 \* \*   \*\*   CSCS: -0.20640603569244648   - ENSMUSG00000048439    -77    -69  -  TATGCTGA   - Pde6b                 -77    -69  +  TTTGCTGA     - Mouse                           tttgctga Rat                             tttgctga Human                           tttgctga Dog                             tttgctga Opossum                         tttgctga                                 \*\*\*\*\*\*\*\*   CSCS: -1.6589117087720509   - Cnga1                 -98    -90  -  AATGCTGA     - Mouse                           aatgctga Human                           -atgctat                                  \*\*\*\*\*     CSCS: 0.2048115228768415   - ENSMUSG00000069743    -93    -85  -  AATGCTGG   - Nr2e3                 -90    -82  +  AATGCTGC     - Mouse                           gcagcatt Human                           gcagcatt Opossum                         gaagaatt Chicken                         gaagaatt X.tropicalis                    gaagaatt CSCS: 0.23856680818984288   - ENSMUSG00000034528   -115   -107  +  TATGCTGC   - ENSMUSG00000044469   -112   -104  +  TTTGCTGA   - ID: Nrl\_1964\_1974\_1     R|C/ N: (5/10)     Z: 4.069864    Consensus:                           NKCTGTGCCYN   - Nrl                   -37    -26  +  CTCTGTGCCCA     - Mouse                           tgggcacagag Rat                             tgggcacagag Human                           tgggcacagag Dog                             agggcaccaag Opossum                         tggacacagaa                                  \*\* \*\*\*  \*    CSCS: -1.249832652932312   - Sag                   -59    -48  -  ATCTGTGCCCA     - Mouse                           tg Rat                             tg Human                           tg Dog                             -g                                  \*   Mouse                           gca-------cagat-- Rat                             gcatct---ccacagat Human                           gcatcctcgctagat-c Dog                             gcaccctgtccaggt-c                                 \*\*\*        \*        CSCS: -1.1403990627152352   - ENSMUSG00000021650    -78    -67  -  CTCTGTGCCTG   - ENSMUSG00000044811    -72    -61  -  CTCTGTGACCA   - ENSMUSG00000048439     -2      9  -  CTCTGTGACCA   - Nr2e3                  83     94  -  GTCTGTGCCTG     - Mouse                           gtctgtgc-- Human                           tcctgaact- Opossum                         ctctgtccta                                   \*\*\*  \*           \*\*\*\*\*\*\*\*\*\*\*\*\*\*\*\*\*\*\*\*\*\*\*\*\*\*\*\*\*\*\*\*\*\*\*  \*   CSCS: -0.9058848980233705   - ENSMUSG00000027481      6     17  +  AGCTGTGCCCT   - ENSMUSG00000026603   -123   -112  -  TGCTGTGCCCT   - Cnga1                -181   -170  -  CTCTGTGCTTT     - Mouse                           ctctgtgcttt Human                           ctctgtgcctt                                 \*\*\*\*\*\*\*\* \*\*   CSCS: -1.779290008902169   - Pde6b                 -22    -11  -  TGCTGTGCCCG     - Mouse                           cgggcaca-gca Rat                             gggcacagcagg Human                           agggacag-gca Dog                             ag--acag-aca Opossum                         agggactg-tga                                  \*             CSCS: 0.9261176502370957   - ID: Pde6g\_1576\_1584\_9     R|C/ N: (6/14)     Z: 3.999445    Consensus:                           RGGCACAGN   - Pde6g                  38     47  -  AGCCACAGG     - Mouse                           agccacagg Human                           ggct----g Dog                             tcac----g                                         \*   CSCS: 1.798174696683984   - Sag                    64     73  +  AAGCACAGG     - Mouse                           aagcacagg Rat                             aagcacagg Human                           gagcataga Dog                             ggg-acgga                                   \* \*  \*    CSCS: -0.4599290500140595   - Sag                   -58    -49  +  GGGCACAGA     - Mouse                           g Rat                             g Human                           g Dog                             g                                 \*   Mouse                           gca-------caga Rat                             gcatct---ccaca Human                           gcatcctcgctaga Dog                             gcaccctgtccagg                                 \*\*\*        \*     CSCS: -1.2235982469627582   - ENSMUSG00000027995     35     44  -  AGCCACAGG   - ENSMUSG00000052353     86     95  -  CGGCACAGT   - Nr2e3                  84     93  +  AGGCACAGA     - Mouse                           tctgtgc--- Human                           cctgaact-- Opossum                         tctgtcctag                                  \*\*\*  \*           \*\*\*\*\*\*\*\*\*\*\*\*\*\*\*\*\*\*\*\*\*\*\*\*\*\*\*\*\*\*\*\*\*\*\*     CSCS: -1.0392181363089315   - Nrl                    43     52  -  AGGCACAGC     - Mouse                           aggcacagc Rat                             ctgcacagc Human                           aggcacagc Dog                             aggcacagc Opossum                         aggtaccac                                   \* \*\*  \*   CSCS: -1.0159861401134582   - Nrl                   -36    -27  -  GGGCACAGA     - Mouse                           gggcacaga Rat                             gggcacaga Human                           gggcacaga Dog                             gggcaccaa Opossum                         ggacacaga                                 \*\* \*\*\*  \*   CSCS: -1.3251993131914674   - ENSMUSG00000027481      7     16  -  GGGCACAGC   - Gnat1                 -42    -33  -  CTGCACAGG     - Mouse                           ctgcacagg Rat                             ctgcacagg Human                           ctgtgcaat Dog                             ctgtgcacc Opossum                         ctcc----t                                 \*\*          CSCS: 1.3577235707120077   - ENSMUSG00000021650    -77    -68  +  AGGCACAGA   - ENSMUSG00000020327    -10     -1  +  CGGCACAGG   - ENSMUSG00000026983     77     86  -  AGGCACACT   - ENSMUSG00000032059    -39    -30  +  TGGCACAGT   - ENSMUSG00000020868    -11     -2  -  GCGCACAGG   - Pde6b                 -21    -12  +  GGGCACAGC     - Mouse                           gggcaca-gc Rat                             ggcacagcag Human                           gggacag-gc Dog                             g--acag-ac Opossum                         gggactg-tg                                 \*            CSCS: 0.8927084935697425   - ID: Nr2e3\_2084\_2094\_1     R|C/ N: (4/7)     Z: 3.9808664    Consensus:                           MAGGCACAGAN   - Nr2e3                  83     94  +  CAGGCACAGAC     - Mouse                           gtctgtgc-- Human                           tcctgaact- Opossum                         ctctgtccta                                   \*\*\*  \*           \*\*\*\*\*\*\*\*\*\*\*\*\*\*\*\*\*\*\*\*\*\*\*\*\*\*\*\*\*\*\*\*\*\*\*  \*   CSCS: -0.9058848980233705   - ENSMUSG00000021650    -78    -67  +  CAGGCACAGAG   - Sag                   -59    -48  +  TGGGCACAGAT     - Mouse                           tg Rat                             tg Human                           tg Dog                             -g                                  \*   Mouse                           gca-------cagat-- Rat                             gcatct---ccacagat Human                           gcatcctcgctagat-c Dog                             gcaccctgtccaggt-c                                 \*\*\*        \*        CSCS: -1.1403990627152352   - Rho                   -29    -18  -  CAGGCACTGAC     - Mouse                           g-------tcagtgcctg Rat                             g-------tcagtgcctg Human                           g-------tcagaaccca Dog                             g-------tcagagcctg Opossum                         g-------ccacagttcc X.tropicalis                    g-------tctcagtgta                                 \*\*\*\*\*\*\*\* \*           CSCS: -0.127975798861171   - ENSMUSG00000026983     76     87  -  CAGGCACACTC   - ENSMUSG00000043760    144    155  +  CAGGCACAAAA   - Nrl                    42     53  -  AAGGCACAGCT     - Mouse                           aaggcacagct Rat                             actgcacagct Human                           caggcacagct Dog                             caggcacagct Opossum                         caggtaccact                                    \* \*\*  \*\*   CSCS: -0.8397313136888972   - Nrl                   -37    -26  -  TGGGCACAGAG     - Mouse                           tgggcacagag Rat                             tgggcacagag Human                           tgggcacagag Dog                             agggcaccaag Opossum                         tggacacagaa                                  \*\* \*\*\*  \*    CSCS: -1.249832652932312   - ID: Pde6g\_294\_302\_8     R|C/ N: (4/7)     Z: 3.9808664    Consensus:                           STGGGATKW   - Pde6g                -103    -94  -  CTGGGATGC     - Mouse                           ctgggatgc Human                           caagg-ttc Dog                             ggaggattt                                    \*\* \*     CSCS: 0.5094102651622908   - Rho                  -118   -109  +  GTGGGATTA     - Mouse                           gt-g--ggatt Rat                             gt-g--ggatt Human                           gcgg--ggatt Dog                             gtgg--ggatt Opossum                         gcgg--ggatt X.tropicalis                    gctt--ggatt                                 \*   \*\*\*\*\*\*\*                                   Mouse                                 Rat                                 Human                                 Dog                                 Opossum                                 X.tropicalis CSCS: NaN   - ENSMUSG00000038797   -107    -98  +  CTGGGATCT   - ENSMUSG00000048439    -41    -32  -  ATGGGATTT   - Pde6a                 -70    -61  -  GAGGGATTA     - Mouse                           taatc------cctc Rat                             taatcc-----cccc Human                           taatct-----ccca Dog                             taatct-----cctg Opossum                         caatcttgtaccctc                                  \*\*\*\*      \*\*     CSCS: -1.5962913817594404   - Gnat1                 -69    -60  -  GTGGGATTT     - Mouse                           gtgggattt Rat                             gtgggattt Human                           gtgggattt Dog                             gtaggattt Opossum                         gcaggattt                                 \*  \*\*\*\*\*\*   CSCS: -1.2576149203369287   - ENSMUSG00000034278   -107    -98  -  CTGGGATGA   - ID: Pde6c\_1366\_1376\_4     R|C/ N: (4/7)     Z: 3.9808664    Consensus:                           NKWGGCAGAAW   - Pde6d                -322   -311  -  GGTGGCAGAAA     - Mouse                           ggtggcaga-aa Rat                             ggtggcaga-aa Human                           cgcgacaga-ga Dog                             tgtgagaga-ga                                  \* \*  \*\*\*\* \*   CSCS: 0.4419085072443286   - ENSMUSG00000019808   -446   -435  -  ATTGGCAGAAA   - ENSMUSG00000027536   -395   -384  +  CGTGGCAGAAT   - Nr2e3                -381   -370  +  AGAGGCAGAAA     - Mouse                           tttctgcctct Human                           gtcctgcctga                                  \* \*\*\*\*\*\*     CSCS: -0.12175256287497035   - Pde6a                -273   -262  +  GAGGGCAGAAA     - Mouse                           g--agggcagaaa Rat                             a--agggcagcga Human                           g--aggtcagaga Dog                             tgcagggcagaga                                    \*\*\* \*\*\*  \*   CSCS: -1.5231168307096112   - Gnat1                -265   -254  -  TTTGGCAGAAT   - ENSMUSG00000026799   -405   -394  +  AGTGGCAGTTT   - ID: Pde6h\_620\_630\_2     R|C/ N: (4/7)     Z: 3.9808664    Consensus:                           MWGGAACAMMN   - ENSMUSG00000020327    -30    -19  +  CTGGAACACCG   - Pde6a                -117   -106  +  CTGGAACACGC     - Mouse                           ctggaacac-gc Rat                             ctggaacat-ac Human                           ctggaacac-ac Dog                             ctggaaccctct                                 \*\*\*\*\*\*\*        CSCS: -0.81902562728175   - Gnb1                  104    115  +  CTGGAACAGCC     - Mouse                           ctggaacagcc Rat                             ctggaacagcc Human                           gtggagcagcc Dog                             gtggagcagcc                                  \*\*\*\* \*\*\*\*\*   CSCS: -0.20181684216809054   - Nrl                   101    112  +  AAAGAACACCT     - Mouse                           atactcacttt- Rat                             atactcacttt- Human                           atactcacttc- Dog                             atactcacctg- Opossum                         acacttacttt-                                 \* \*\*\* \*\* \* \*   CSCS: -1.249832652932312   - ENSMUSG00000037295   -167   -156  +  TAGGAACACCG   - ENSMUSG00000054757   -150   -139  -  CTGGAACAAAT   - Pde6b                 -10      1  +  CAGGAACACCN     - Mouse                           cagga Rat                             g--ga Human                           c---- Dog                             c-cgg Opossum                         g--ag Mouse                           cacc Rat                             cacc Human                           cacc Dog                             cacc Opossum                         cacc Chicken                         caca X.tropicalis                    ---- CSCS: NaN   - ID: Pde6h\_2027\_2034\_11     R|C/ N: (5/11)     Z: 3.8301704    Consensus:                           NTGCCTGN   - Nr2e3                  82     90  -  GTGCCTGT     - Mouse                           gtgc------ Human                           gaact----- Opossum                         gtcctaggct                                 \*  \*           \*\*\*\*\*\*\*\*\*\*\*\*\*\*\*\*\*\*\*\*\*\*\*\*\*\*\*\*\*\*\*\*\*\*\*  \*\*   CSCS: -1.1144797583023751   - ENSMUSG00000041669     63     71  -  TTGCCTGG   - ENSMUSG00000026983     80     88  +  GTGCCTGG   - ENSMUSG00000036686     67     75  -  GTGCCTGG   - Pde6g                  90     98  +  CAGCCTGA     - Mouse                           aaaggacc Human                           gaaagaca Dog                             gacaggcc                                  \*  \* \*    CSCS: 0.1407313879721225   - Pde6g                  67     75  +  TTGCCTGC     - Mouse                           ctcaccaa Human                           ctcaccaa Dog                             ctcaccaa                                 \*\*\*\*\*\*\*\*   CSCS: -2.2892305776798603   - Nrl                    46     54  +  GTGCCTTT     - Mouse                           aaaggcac Rat                             aactgcac Human                           acaggcac Dog                             gcaggcac Opossum                         gcaggtac                                     \* \*\*   CSCS: -0.3830176273821628   - ENSMUSG00000030281     77     85  -  AGGCCTGA   - Gnat1                  49     57  +  TTGCCTGC   - ENSMUSG00000070427     56     64  +  CTGCCTGC   - ENSMUSG00000030643     95    103  +  ATGCCTGG   - Pde6a                  61     69  +  GAGCCTGA     - Mouse                           g Rat                             a Human                           c Dog                             a Opossum                         - Mouse                           gcctga Rat                             cctgag Human                           tcccag Dog                             ctttag Opossum                         --tgag Chicken                         cataga X.tropicalis                    ------ CSCS: 1.6190451573532527   - ID: Pde6d\_70\_80\_7     R|C/ N: (5/11)     Z: 3.8301704    Consensus:                           WSAGACCCWWR   - Gnat1                -283   -272  -  ACAGACCCTTT     - Mouse                           acagacccttt Rat                             acagacccttt Human                           gcagaaccttg Dog                             ataggaccctg                                   \*\*  \*\* \*    CSCS: -0.5366463644690751   - ENSMUSG00000027481   -175   -164  -  AGAGACCCTGG   - ENSMUSG00000026433     30     41  -  TTAGACCCTTT   - Rho                  -204   -193  +  AGACACCCTTT     - Mouse                           agacacccttt Rat                             ----------- Human                           ggggaccttct Dog                             ----------t X.tropicalis                    aaatacacttt CSCS: 0.7797599479838877   - Rho                   -45    -34  -  CCAGACCCTTA     - Mouse                           taa--gggtct-----gg Rat                             taa--gggtct-----gg Human                           taa--gggtct------g Dog                             taa--cggcctgg---gg Opossum                         taa--ggacct------- X.tropicalis                    aaa--ggaccc-----gg                                  \*\*\*\* \*  \*   \*\*\*     CSCS: -1.1309997950636825   - Gnb1                 -182   -171  +  GAAGACCCTTG     - Mouse                           gaagacccttg Rat                             gaagacccttg                                 \*\*\*\*\*\*\*\*\*\*\*   CSCS: -1.5278349030144454   - ENSMUSG00000013150     -5      6  +  AGAGACCCCTG   - ENSMUSG00000043760   -101    -90  -  AGAGACCCAAA   - ENSMUSG00000043760   -266   -255  -  AGAGACCCCAG   - Pde6g                -142   -131  +  TCAGACCCTTA     - Mouse                           taagggtct----ga Human                           caagggctccaagga Dog                             caagggctcgaagga                                  \*\*\*\*\*       \*\*   CSCS: -0.3026480787669187   - ENSMUSG00000036636   -343   -332  -  TGAGACCCTGA   - ENSMUSG00000037295   -316   -305  +  CAAGACCCTTA   - Pde6b                -167   -156  -  AGAGACCCGGA     - Mouse                           tccgggtctc-t Rat                             gccgggcctc-t Human                           tcccagggtc-t                                  \*\*  \*  \*\*\*\*   CSCS: -0.6016605474172871 |

Page by: Charles Danko & Maochun Qin; SUNY Upstate Medical University.
