# Supplementary material for: Bioinformatic identification of novel putative photoreceptor specific cis-elements
Source: BMC Bioinformatics. 2007 Oct 22;8:407. doi: 10.1186/1471-2105-8-407 (PMC2225425; doi:10.1186/1471-2105-8-407)
Supplement: Additional file 1 — Explanation of Supplementary Data. Detailed information on reading HTML formatted supplementary data. [file 1471-2105-8-407-S1.ZIP › r.TS.html]

cis-Browser 

Predictions via cis-Browser

|  |
| --- |
| - ID: Sag\_21\_31     R|C/ N: (9/19)     Z: 4.4803023    Consensus:                           CTGGAGACKNR   - Sag                 -1980  -1969  +  CTGGAGACGTG   - Pde6b                -474   -463  -  GTGGAGACGTC     - Mouse                           gac---------gtctccac Rat                             --------------------                                    \*\*\*\*\*\*\*\*\*           CSCS: 2.6496829088661134   - Pde6d                -538   -527  -  CCGGAGACGTG     - Mouse                           ccgga--gacgtg Rat                             g----agac-atg Human                           tcaaaagaagatg Dog                             tcagaagaggatg                                            \*\*   CSCS: 2.1719137368200396   - Pde6d               -1533  -1522  -  CTGGTGACGTG     - Mouse                           ctggtgacgtg Rat                             t---------- Human                           c---------- Dog                             a---------- CSCS: 1.977933443013073   - Pde6d               -1218  -1207  -  TTGGAGACGCC     - Mouse                           ttggagac-gcc Rat                             ttggagatgc-c Human                           ttggaaacctcc Dog                             ttggacatctcc                                 \*\*\*\*\* \*    \*   CSCS: -0.14773773196091178   - Nr2e3               -1847  -1836  +  CTGGAGACGCT   - ENSMUSG00000069763  -1390  -1379  +  CTGGAGATGTG   - Pde6g               -1356  -1345  -  CTGGAGAGGTG   - ENSMUSG00000025482   -234   -223  +  CTGGAGATGTG   - ENSMUSG00000025482   -253   -242  +  CTGGAGACTCG   - Gnat1                -207   -196  +  CTGGAGACCTG     - Mouse                           cag-gtct----ccag Rat                             cag-ttct----ccag Human                           caa-ccct----ccag Dog                             caa-ccct----ctgg Opossum                         caa-tcatggggccag                                 \*\* \*   \*    \*  \*   CSCS: -0.2549681338063851   - ENSMUSG00000018919   -126   -115  -  CTGGAGAGGTG   - ENSMUSG00000025347   -926   -915  -  CTGCAGACGTG   - ENSMUSG00000025347  -1106  -1095  -  CTGGAGACCTG   - ENSMUSG00000031957   -554   -543  +  CTGGAGACAGG   - ENSMUSG00000031957  -1610  -1599  -  CTGGAGAGGTG   - Gnb1                  286    297  +  ACTGAGACGTG   - ENSMUSG00000035769  -1650  -1639  +  TTGGAGACGAC   - ENSMUSG00000027442   -162   -151  -  CTGGAGACAGC   - Nrl                    28     39  -  CTGGAGACTAA     - Mouse                           ctggagactaa Rat                             ccagagactag Human                           caggagactga Dog                             ccagagactgg Opossum                         ctaaaggtaat                                 \*   \*\*        CSCS: 0.11717181121240394   - Nrl                  -663   -652  -  CTGGAGACTAA   - Rho                  -680   -669  -  ATGGAGACGGG     - Mouse                           cccgtctccat Rat                             cccatctccat Human                           cctgtctccac Dog                             tcccctgcctt                                  \*     \*\*     CSCS: -0.5109833815667115   - ENSMUSG00000031445   -137   -126  -  AGAGAGACGTG   - ENSMUSG00000024803   -841   -830  -  CTGGAGACTCA   - ENSMUSG00000019987   -605   -594  +  CTGGAGACTGT   - ID: Rho\_1799\_1811     R|C/ N: (4/4)     Z: 4.070672    Consensus:                           ACASCCTTTCCTY   - Rho                  -202   -189  +  ACACCCTTTCCTT     - Mouse                           acaccctttcctt Rat                             ------------- Human                           ggaccttctcctc Dog                             --------tctgc X.tropicalis                    atacactttcttc CSCS: 0.5042403334233109   - Cnga1                -284   -271  -  ACACCCTCTCCTC     - Mouse                           ac-accctctcctc Rat                             catatcctcctatc Human                           gcttccttctcttc                                      \* \*\*   \*\*   CSCS: 0.030450066335843753   - Nr2e3                -887   -874  -  ACATCCTTTCCTG     - Mouse                           acatcctttcc-tg Human                           gcagccttttc-t- Dog                             actgcctttcctt-                                  \*  \*\*\*\*\* \* \*    CSCS: -1.2614660577690369   - Gnb1                 -699   -686  -  GCAGCCTTTCCTT     - Mouse                           aaggaaaggctgc Rat                             gaggaaaggctgc                                  \*\*\*\*\*\*\*\*\*\*\*\*   CSCS: -0.4751698975199642   - ID: Pde6d\_2113\_2125     R|C/ N: (4/4)     Z: 4.070672    Consensus:                           GAGCCAGCGTGGK   - Pde6d                 112    125  +  GAGCCAGCGTGGG     - Mouse                           cccacgct-ggctc Rat                             cccacgctggc-tc Human                           ctcactct-gg-tc Dog                             cccacgct-ggccc Chicken                         gccgggct-cgcct                                   \*   \*\*         CSCS: 0.6783633886091904   - Pde6a                -281   -268  +  GAGCCAGCGAGGG     - Mouse                           gagccagcg--aggg Rat                             cagccacca--aggg Human                           aactaacag--aggt Dog                             aaacaccctgcaggg                                  \*         \*\*\*    CSCS: -0.17694545855345192   - Nr2e3                   9     22  +  GAGCCAGGGTGGT     - Mouse                           accac-----------cctggctc Human                           acagc-----------acaggctc Opossum                         a------------------ggcgc X.tropicalis                    g---------------------tc                                      \*\*\*\*\*\*\*\*\*\*\*       \*   CSCS: 1.4880476182856892   - Gnb1                 -626   -613  +  GAGTCAGCGTGGT     - Mouse                           gagtcagcgt----ggt Rat                             aagccaacgt----ggt Human                           aaataggtgttggaggt                                  \*      \*\*    \*\*\*   CSCS: 0.3381136648873603   - ID: Pde6a\_1684\_1696     R|C/ N: (4/4)     Z: 4.070672    Consensus:                           AGTCAWTGTCAGG   - Pde6a                -317   -304  +  AGTCATTGTCAGG     - Mouse                           agtcattgtcagg Rat                             ------tgtcagt Human                           agtaaatgtcagt Dog                             agcataggt-agg                                        \*\* \*\*    CSCS: -0.17694545855345192   - Rho                  -403   -390  +  AGCCATTGTCAGG     - Mouse                           agccattgtcagg Rat                             agctggtgtcagg Human                           a------------ Dog                             ggctgggctcaga CSCS: 1.7115269091125789   - Cnga1               -1747  -1734  +  GGTCAATGTCAGG     - Mouse                           cctgacattgacc Rat                             cctgatgttgacc                                 \*\*\*\*\*  \*\*\*\*\*\*   CSCS: -0.01923322843080425   - Nrl                    59     72  -  AGTCACTGTCAGA     - Mouse                           agtc-actgtcaga Rat                             agtc-accgtcaga Human                           ggtc-agtgccaga Dog                             ggtc-agtgtcaga Opossum                         agtc-aaagttata                                  \*\*\*\*\*  \*  \* \*   CSCS: -0.8421613845489426   - ID: Rho\_483\_495     R|C/ N: (4/4)     Z: 4.070672    Consensus:                           RMACTGCTAGTGR   - Rho                 -1518  -1505  +  AAACTGCTAGTGG     - Mouse                           aaactgctagtgg Rat                             aaactgctggagg Human                           aaactacgagagg Dog                             aaacagcgagagg                                 \*\*\*\*  \*  \* \*\*   CSCS: -0.024626276652108082   - Pde6g               -1156  -1143  -  GCACTGCTAGTGT   - Sag                  -831   -818  +  GAACTGCTAGTAA     - Mouse                           ga-actg------------ctagtaa Rat                             gtca---------------ttagtaa Human                           gagactgtagaataattcccaagcaa Dog                             aagaccatgataacctcc-cacacaa                                    \*                    \*\*   CSCS: 0.7109168836172539   - Gnb1                 -887   -874  -  CTTCTGCTAGTGG     - Mouse                           ccactagcagaag Rat                             tgcccac------ Human                           ctcccacaatgaa                                    \* \*          CSCS: 1.8366215717419372   - ID: Gnb1\_1686\_1698\_1     R|C/ N: (4/4)     Z: 4.941698    Consensus:                           RCCTTTCRGCCCT   - Gnb1                 -315   -302  +  GCCTTTCGGCCCT     - Mouse                           gcctttcggccct Rat                             gcttttccgctct Human                           gcccaccggcccc                                 \*\*    \* \*\* \*    CSCS: 0.024319080305123347   - Pde6g                -375   -362  -  GCCTTTCAGCCCT     - Mouse                           gcctttcagccct Dog                             caccttctctctc                                   \* \*\*\*   \*     CSCS: 0.6645664032364412   - Rho                  -289   -276  -  ACCTTTGGGCCCT     - Mouse                           agggcccaaa Rat                             agggcccaaa Human                           agaggcccat Dog                             -ggagccaga                                  \*   \*\*   \*\*\*\*\*\*\*\*\*\*\*\*\*\*\*\*\* \*\*\*\*\*\*    CSCS: 0.3537508513127135   - Pde6a                -272   -259  -  TCCTTTCTGCCCT     - Mouse                           agggcagaaagga Rat                             agggcagcgagga Human                           aggtcagagaaga Dog                             agggcagagaaaa                                 \*\*\* \*\*\*  \*  \*   CSCS: -1.6377554542824095   - ID: Gnb1\_1687\_1698\_1     R|C/ N: (4/4)     Z: 4.941698    Consensus:                           CCTTTCRGCCCT   - Gnb1                 -314   -302  +  CCTTTCGGCCCT     - Mouse                           cctttcggccct Rat                             cttttccgctct Human                           cccaccggcccc                                 \*    \* \*\* \*    CSCS: 0.20019931604685723   - Rho                  -289   -277  -  CCTTTGGGCCCT     - Mouse                           agggcccaaa Rat                             agggcccaaa Human                           agaggcccat Dog                             -ggagccaga                                  \*   \*\*   \*\*\*\*\*\*\*\*\*\*\*\*\*\*\*\*\* \*\*\*\*\*\*   CSCS: 0.2659543331421266   - Pde6a                -272   -260  -  CCTTTCTGCCCT     - Mouse                           agggcagaaagg Rat                             agggcagcgagg Human                           aggtcagagaag Dog                             agggcagagaaa                                 \*\*\* \*\*\*  \*     CSCS: -1.487115483846437   - Pde6g                -375   -363  -  CCTTTCAGCCCT     - Mouse                           cctttcagccct Dog                             accttctctctc                                  \* \*\*\*   \*     CSCS: 0.4469156094481655   - ID: Opn1mw\_101\_108\_15     R|C/ N: (4/4)     Z: 4.941698    Consensus:                           NCTGGGAS   - Gnb1                   97    105  +  GCTGGGAC     - Mouse                           gctgggac Rat                             gctgggac Human                           gccgggag Dog                             --cgggag                                    \*\*\*\*    CSCS: 0.776249804453297   - Sag                   103    111  +  ACTGGGTG     - Mouse                           actgggtg Rat                             -----tcg Human                           cctggttg Dog                             gctggctg                                        \*   CSCS: 0.42404493883721645   - Nrl                   123    131  -  TCTGGGAC     - Mouse                           ccctcaag- Rat                             cacccacga Human                           cacccaggt Dog                             cacccaggt Opossum                         --cttaggt                                   \*  \* \*    CSCS: 0.454833432516318   - Pde6d                 119    127  +  CGTGGGAG     - Mouse                           ctcccacg Rat                             ctcccacg Human                           ttctcact Dog                             ctcccacg Chicken                         gcgccggg                                     \*      CSCS: 0.7348869403459909   - ID: Smug1\_1207\_1217\_8     R|C/ N: (4/5)     Z: 4.283107    Consensus:                           RGCTCCCTRMK   - Nr2e3                -111   -100  +  GGCTCCCAGGG   - Sag                  -112   -101  +  AGCTCCCTGCT     - Mouse                           agctccctgct Rat                             agctccctgct Human                           ----ctctgct Dog                             tccactctgct                                     \* \*\*\*\*\*   CSCS: -0.1567396509131853   - Pde6g                 -86    -75  +  GGCTCCCTCAA     - Mouse                           tt------ga-gggagcc Human                           cc------gagggggggc Dog                             actcaggagggtgagggc                                         \*   \*  \* \*   CSCS: 0.547831332451511   - ENSMUSG00000042200    -91    -80  +  AGCTCCCTGAG   - Pde6b                -104    -93  +  GGCTCCCTAAT     - Mouse                           ggctccc--taat Rat                             ggctccc--taat Human                           gggttcc--taat Dog                             ggcctcg--taat Opossum                         gattccccataat                                 \*    \*   \*\*\*\*   CSCS: -0.13988956771286906   - ID: Rho\_286\_294\_9     R|C/ N: (4/5)     Z: 4.283107    Consensus:                           RRGATGGCY   - Sag                  -213   -204  +  TGGATGGCT     - Mouse                           tggatggc--t Rat                             tggat-ac--t Human                           tgggtgac--t Dog                             ------gc--t                                        \*\*\*\*   CSCS: 0.2615010474051899   - Cnga1                -228   -219  +  GAGATGGGC     - Mouse                           gcccatctc Rat                             acccatctc Human                           atccattgc                                   \*\*\*\*  \*   CSCS: -0.6257531783326754   - Pde6d                -226   -217  -  GCGATGGCT     - Mouse                           gcgatggct Rat                             gcggtggct Human                           gggttggct Dog                             gggttggct                                 \* \* \*\*\*\*\*   CSCS: -0.017512750611808077   - Pde6d                -255   -246  -  GAGATGCCT     - Mouse                           gagatgcct Rat                             gaag--cct Human                           ggga--cct Dog                             gggg--ccc                                 \*     \*\*    CSCS: 1.9045116290341366   - Pde6g                -254   -245  +  AAGATGGCC     - Mouse                           ggccatctt Dog                             ggctgttgt                                 \*\*\*  \*  \*   CSCS: -0.34510472345838406   - ENSMUSG00000021439   -251   -242  +  AGGATGGCT   - ID: Pde6d\_1613\_1623\_1     R|C/ N: (4/5)     Z: 4.283107    Consensus:                           TGWAGTCCCTG   - Pde6d                -388   -377  +  TGTAGTCCCTG     - Mouse                           c-agggactaca Rat                             --aggaactaca Human                           c-ggggactaca Dog                             c-ggggactaca                                  \* \*\* \*\*\*\*\*\*   CSCS: -0.5341812269959745   - Pde6g                -414   -403  -  TGTAGTCCCAT   - ENSMUSG00000069763   -316   -305  -  TGTAGTGCCTG   - Nr2e3                -298   -287  +  TGAAGTCCCTG     - Mouse                           cagggac-------ttca Human                           ---ggac-------ttta                                    \*\*\*\*\*\*\*\*\*\*\*\*\* \*   CSCS: -1.1727281762162   - Pde6b                -387   -376  -  AAAAGTCCCTG     - Mouse                           cagggactttt Rat                             cagggactttt Human                           ctggggccacc                                 \* \*\*\* \*       CSCS: 0.8728579338931468   - ID: Nr2e3\_1095\_1105\_5     R|C/ N: (4/5)     Z: 4.283107    Consensus:                           KNRGGCAGAMN   - Rho                  -220   -209  +  TGGGGCAGACA     - Mouse                           tggg-gcagac-----------a Rat                             cagg-gcaaacaa---------- Human                           tggg-acagacaagtcatgcaga Dog                             gggg-gcgggcct----------                                   \*\*\* \*   \*               CSCS: -1.6483848437895317   - ENSMUSG00000054446   -318   -307  -  AAAGGCAGAAT   - Pde6g                -119   -108  -  GTGGGCAGAAG     - Mouse                           gtgggcagaag Human                           atggagaggag Dog                             acaagg-gaag                                        \* \*\*   CSCS: 0.264338195378701   - Pde6a                -273   -262  +  GAGGGCAGAAA     - Mouse                           g--agggcagaaa Rat                             a--agggcagcga Human                           g--aggtcagaga Dog                             tgcagggcagaga                                    \*\*\* \*\*\*  \*   CSCS: -1.5231168307096112   - Gnat1                -193   -182  +  GGGGGCAGAGG     - Mouse                           cct-ctgccc-------c----c Rat                             cct-tta-cc------cc----c Human                           ccggctgtccttctg-tc----c Dog                             cct-ctctat------ga----g Opossum                         ttc-cccctt------ta----c                                                \*  \*\*\*\*    CSCS: 0.48028881019342373   - Gnat1                -265   -254  -  TTTGGCAGAAT   - ID: cngb3\_1726\_1736\_4     R|C/ N: (4/5)     Z: 4.283107    Consensus:                           MARGCACAGRW   - Gnat1                 -43    -32  -  CCTGCACAGGT     - Mouse                           cctgcacaggt Rat                             cctgcacaggc Human                           cctgtgcaatc Dog                             cctgtgcaccc Opossum                         cctcc----tt                                 \*\*\*           CSCS: 1.5691842441682395   - ENSMUSG00000030669    -24    -13  +  GAGGCACAGGA   - Sag                    63     74  +  AAAGCACAGGT     - Mouse                           aaagcacaggt Rat                             aaagcacaggt Human                           agagcatagag Dog                             aggg-acggag                                 \*  \* \*  \*     CSCS: -0.45063350407481917   - Nr2e3                  83     94  +  CAGGCACAGAC     - Mouse                           gtctgtgc-- Human                           tcctgaact- Opossum                         ctctgtccta                                   \*\*\*  \*           \*\*\*\*\*\*\*\*\*\*\*\*\*\*\*\*\*\*\*\*\*\*\*\*\*\*\*\*\*\*\*\*\*\*\*  \*   CSCS: -0.9058848980233705   - Nrl                    42     53  -  AAGGCACAGCT     - Mouse                           aaggcacagct Rat                             actgcacagct Human                           caggcacagct Dog                             caggcacagct Opossum                         caggtaccact                                    \* \*\*  \*\*   CSCS: -0.8397313136888972   - ID: Nr2e3\_1114\_1125\_2     R|C/ N: (4/5)     Z: 4.283107    Consensus:                           NAGGAAAGGAKG   - Nr2e3                -169   -157  +  CAGCAAAGGATG   - Pde6g                -180   -168  -  CAGGAAAGGAGG     - Mouse                           caggaaaggag-g Human                           cgggacaggga-g Dog                             caggacagggatg                                 \* \*\*\* \*\*\*   \*   CSCS: -0.7756618788185099   - Pde6a                -269   -257  +  GCAGAAAGGATG     - Mouse                           gcagaaaggatg Rat                             gcagcgaggaca Human                           tcagagaagaca Dog                             gcagagaaaaca                                  \*\*\*  \*  \*     CSCS: -0.32283800342162805   - ENSMUSG00000074336   -159   -147  +  TAGGAAAGGAGG   - Rho                  -201   -189  -  AAGGAAAGGGTG     - Mouse                           caccctttcctt Rat                             ------------ Human                           gaccttctcctc Dog                             -------tctgc X.tropicalis                    tacactttcttc CSCS: 0.3407794697129662   - ID: Gnb3\_560\_570\_2     R|C/ N: (5/8)     Z: 4.0735784    Consensus:                           SCTGGGAARNS   - ENSMUSG00000031957   -381   -370  +  GCTGGGAAGCC   - ENSMUSG00000024803   -433   -422  -  GCTGGGAAGTG   - ENSMUSG00000024803   -267   -256  -  ATCGGGAAGCC   - Rho                  -361   -350  +  CCTGGGAAGAG     - Mouse                           cct--gggaagag Rat                             cct--gggaagag Human                           ctt--gtggggga Dog                             ctt--gtggggaa                                 \* \*\*\*\* \*  \*     CSCS: -0.07066170190808235   - Pde6d                -336   -325  +  GCTGGGAAATG     - Mouse                           catttcccagc Rat                             catttcccagc Human                           catttcccagc Dog                             catttcccagc                                 \*\*\*\*\*\*\*\*\*\*\*   CSCS: -1.5102709612362777   - Nrl                  -420   -409  +  GCTGGGAACGC   - Gnat1                -365   -354  +  GCTGGGAAAAC     - Mouse                           gttttcccagc Rat                             gcttgcccagc Human                           gcctgcccggc Dog                             c-ctgcccagc                                    \* \*\*\* \*\*   CSCS: -0.027383339736015515   - ENSMUSG00000066515   -221   -210  +  CCTGGGAAGAT   - Pde6b                -418   -407  -  CCTGGGAAGGT     - Mouse                           accttcccagg- Rat                             accttcccagg- Human                           acctccccagc-                                 \*\*\*\* \*\*\*\*\* \*   CSCS: -0.8429313761596674   - ID: Opn1mw\_1260\_1268\_9     R|C/ N: (5/8)     Z: 4.0735784    Consensus:                           RGACAGTGW   - Gnat1                  38     47  -  GGACAGGGT     - Mouse                           ggacagggt Rat                             ggacagggt Human                           ggacagagt Dog                             ---caggat Opossum                         gggcagaaa X.tropicalis                    caagagag- CSCS: -0.5814596514566847   - Nr2e3                 -31    -22  -  ACACAGTGT   - Sag                    53     62  -  CGACAGTGA     - Mouse                           tcactgtcg Rat                             tcaccatct Human                           tcatcatct Dog                             tcacc-ccg                                 \*\*\*    \*    CSCS: -0.049364112697844145   - Pde6g                   0      9  +  GGCCAGTGT     - Mouse                           acactggcc Human                           gtgccagcc Dog                             gtgccgg-c                                    \*  \* \*   CSCS: 0.8316013730427143   - ENSMUSG00000024233    104    113  -  AGACAGTGA   - Nrl                    61     70  +  TGACAGTGA     - Mouse                           tc-actgtca Rat                             tc-accgtca Human                           tc-agtgcca Dog                             tc-agtgtca Opossum                         tc-aaagtta                                 \*\*\*\*  \*  \*   CSCS: -0.7067729670354492   - ENSMUSG00000049152      2     11  +  AGACAGTGC   - ENSMUSG00000031445    -22    -13  -  GGTCAGTGT   - ID: Pde6d\_70\_80\_3     R|C/ N: (5/8)     Z: 4.0735784    Consensus:                           RRAGACCCTTN   - ENSMUSG00000024866   -261   -250  +  AGAGACCCTTG   - ENSMUSG00000034913   -335   -324  +  GGAGACCCTTC   - Gnat1                -283   -272  -  ACAGACCCTTT     - Mouse                           acagacccttt Rat                             acagacccttt Human                           gcagaaccttg Dog                             ataggaccctg                                   \*\*  \*\* \*    CSCS: -0.5366463644690751   - Rho                  -204   -193  +  AGACACCCTTT     - Mouse                           agacacccttt Rat                             ----------- Human                           ggggaccttct Dog                             ----------t X.tropicalis                    aaatacacttt CSCS: 0.7797599479838877   - Gnb1                 -182   -171  +  GAAGACCCTTG     - Mouse                           gaagacccttg Rat                             gaagacccttg                                 \*\*\*\*\*\*\*\*\*\*\*   CSCS: -1.5278349030144454   - Pde6g                -142   -131  +  TCAGACCCTTA     - Mouse                           taagggtct----ga Human                           caagggctccaagga Dog                             caagggctcgaagga                                  \*\*\*\*\*       \*\*   CSCS: -0.3026480787669187   - ENSMUSG00000074336   -338   -327  +  TGAGACCCTGT   - Pde6b                -167   -156  -  AGAGACCCGGA     - Mouse                           tccgggtctc-t Rat                             gccgggcctc-t Human                           tcccagggtc-t                                  \*\*  \*  \*\*\*\*   CSCS: -0.6016605474172871 |

Page by: Charles Danko & Maochun Qin; SUNY Upstate Medical University.
