# Supplementary material for: Bioinformatic identification of novel putative photoreceptor specific cis-elements
Source: BMC Bioinformatics. 2007 Oct 22;8:407. doi: 10.1186/1471-2105-8-407 (PMC2225425; doi:10.1186/1471-2105-8-407)
Supplement: Additional file 1 — Explanation of Supplementary Data. Detailed information on reading HTML formatted supplementary data. [file 1471-2105-8-407-S1.ZIP › ROP2.html]

cis-Browser 

Predictions via cis-Browser

|  |
| --- |
| - ID: Opn1mw\_1904\_1915\_1     R|C/ N: (4/4)     Z: 5.0822554    Consensus:                           WTAAGAGATCAG   - Opn1mw                -97    -85  +  TTAAGAGATCAG     - 0.11568627450980393             Ratio: Mouse                           ttaagagatcag Rat                             ttaagagatcag Human                           ttaagagatcag Dog                             ttaagagatcag Opossum                         ttaagagatcag X.tropicalis                    ttaagagataaa                                   \*\*\*\*\*\*\*\*\* \*    CSCS: -1.7901212098465105   - cnga3                -122   -110  -  TTAAGGGATCAG     - 0.6060606060606061              Ratio: Mouse                           ctgatcccttaa Rat                             ctgatcccttaa Human                           ctgatcctctgc Dog                             ctgatcccttgc                                   \*\*\*\*\*\*\*  \*     CSCS: -0.9309443787631781   - Opn1sw                -94    -82  +  CTAAGAGATCTC     - 0.8140655105973025              Ratio: Mouse                           gagatctcttag Rat                             gagatctcttag Human                           gaaatccctaaa Dog                             gagctgtccaag Opossum                         gggacccttgag                                   \*         \*    CSCS: -0.42937732572149695   - Smug1                 -55    -43  +  AGTAGAGATCAG     - 1.3846153846153846              Ratio: Mouse                           ctgatctctact Rat                             cttatc--tact Human                           ctaaccgttagt                                   \*\* \* \*  \*\* \*   CSCS: 0.8540536158360592   - ID: Opn1mw\_1904\_1915\_1     R|C/ N: (4/4)     Z: 4.5303755    Consensus:                           WTAAGAGATCAG   - Opn1mw                -97    -85  +  TTAAGAGATCAG     - 0.11568627450980393             Ratio: Mouse                           ttaagagatcag Rat                             ttaagagatcag Human                           ttaagagatcag Dog                             ttaagagatcag Opossum                         ttaagagatcag X.tropicalis                    ttaagagataaa                                   \*\*\*\*\*\*\*\*\* \*    CSCS: -1.7901212098465105   - cnga3                -122   -110  -  TTAAGGGATCAG     - 0.6060606060606061              Ratio: Mouse                           ctgatcccttaa Rat                             ctgatcccttaa Human                           ctgatcctctgc Dog                             ctgatcccttgc                                   \*\*\*\*\*\*\*  \*     CSCS: -0.9309443787631781   - Opn1sw                -94    -82  +  CTAAGAGATCTC     - 0.8140655105973025              Ratio: Mouse                           gagatctcttag Rat                             gagatctcttag Human                           gaaatccctaaa Dog                             gagctgtccaag Opossum                         gggacccttgag                                   \*         \*    CSCS: -0.42937732572149695   - Smug1                 -55    -43  +  AGTAGAGATCAG     - 1.3846153846153846              Ratio: Mouse                           ctgatctctact Rat                             cttatc--tact Human                           ctaaccgttagt                                   \*\* \* \*  \*\* \*   CSCS: 0.8540536158360592 |

Page by: Charles Danko & Maochun Qin; SUNY Upstate Medical University.
