# Supplementary material for: Bioinformatic identification of novel putative photoreceptor specific cis-elements
Source: BMC Bioinformatics. 2007 Oct 22;8:407. doi: 10.1186/1471-2105-8-407 (PMC2225425; doi:10.1186/1471-2105-8-407)
Supplement: Additional file 1 — Explanation of Supplementary Data. Detailed information on reading HTML formatted supplementary data. [file 1471-2105-8-407-S1.ZIP › RXR.html]

cis-Browser 

Predictions via cis-Browser

|  |
| --- |
| - ID: Gnb3\_1797\_1809     R|C/ N: (5/7)     Z: 3.8463635    Consensus:                           NACAGGGTCATMK   - Gnb3                 -204   -191  +  AACAGGGTCATCT     - 0.7847882454624028              Ratio: Mouse                           agatgac--------cctgtt Rat                             agatgac--------cctgtt Human                           agatggc--------cccatt Dog                             aggtggc--------cc-att Opossum                         agatgatggcaaacatccttc                                   \*\* \*\*           \*  \*    CSCS: -0.5577113005350727   - ENSMUSG00000029415  -1855  -1842  -  AACATGGTCATCT   - ENSMUSG00000037060  -1126  -1113  +  AACAGGGTCAAAG   - cngb3                  37     50  +  TACAGGGTCATAT     - 1.4830316742081449              Ratio: Mouse                           tacagggtcatat Rat                             tacagggtcatag Human                           caca--gtcataa Dog                             -acagagttgtaa Opossum                         caca--ggcagaa Chicken                         cacg--gtaaaag                                   \*\*   \*    \*    CSCS: 0.9021743976791854   - Pde6c               -1847  -1834  +  CACAGGGTCATGG     - 0.7818181818181819              Ratio: Mouse                           cacagggtcatgg Rat                             cacagagtcatga                                   \*\*\*\*\* \*\*\*\*\*\*    CSCS: -0.191482117569823   - Gngt2                -770   -757  -  GGTAGGGTCATCT     - 0.7867132867132868              Ratio: Mouse                           ag----------atgaccctacc Rat                             ag----------atgaccctgcc Human                           aggatgggtcccagggccccact                                   \*\*          \* \* \*\*\*  \*    CSCS: -0.504303059896423   - Pde6h                -197   -184  -  GACAGGGGCATCT     - 0.8801261829652998              Ratio: Mouse                           agatgcccctgtc Rat                             agatgcccttgtc Human                           atgaacccaagtc Dog                             gagagcccaagtc                                   \*\*\*  \*\*\*   CSCS: -0.2155677347036258 |

Page by: Charles Danko & Maochun Qin; SUNY Upstate Medical University.
