# Supplementary material for: Bioinformatic identification of novel putative photoreceptor specific cis-elements
Source: BMC Bioinformatics. 2007 Oct 22;8:407. doi: 10.1186/1471-2105-8-407 (PMC2225425; doi:10.1186/1471-2105-8-407)
Supplement: Additional file 1 — Explanation of Supplementary Data. Detailed information on reading HTML formatted supplementary data. [file 1471-2105-8-407-S1.ZIP › TATAlike.html]

cis-Browser 

Predictions via cis-Browser

|  |
| --- |
| - ID: Pde6g\_1027\_1034\_10     R|C/ N: (4/5)     Z: 4.442663    Consensus:                           MSATAAGM   - Gnat2                 -41    -33  +  ACATAAAG     - 0.19454225352112675             Ratio: Mouse                           acataaag Rat                             acataaag Human                           atataaag Dog                             atataaag Opossum                         acataaag                                   \* \*\*\*\*\*\*   CSCS: -1.696467562630248   - Gnb3                  -21    -13  -  AGATAAGA   - Pde6c                 -23    -15  +  CCATAAGC     - 0.5542521994134897              Ratio: Mouse                           ccataagc Rat                             ccataagc Human                           ccctaagc Dog                             ctgtaagc Opossum                         cgctaagc Chicken                         cactaagc X.tropicalis                    cactaagc                                   \*\*\*\*\*   CSCS: -1.2203126961822135   - ENSMUSG00000002372    -66    -58  -  CCATAAGC   - Arr3                  -43    -35  +  ATATAAGA     - 0.8478260869565217              Ratio: Mouse                           atataaga Rat                             atataaga Human                           gtataaga Dog                             gtataaaa Opossum                         ccataaaa                                   \*\*\*\* \*   CSCS: -0.32127293892259806   - ID: Gnb3\_1979\_1987\_1     R|C/ N: (5/8)     Z: 4.3349175    Consensus:                           CWCTTATMN   - Gnb3                  -22    -13  +  CTCTTATCT   - Arr3                  -43    -34  -  CTCTTATAT     - 0.7536231884057971              Ratio: Mouse                           atataagag Rat                             atataagag Human                           gtataagag Dog                             gtataaaag Opossum                         ccataaaag                                   \*\*\*\* \*\*   CSCS: -0.5469448206439848   - cnga3                -106    -97  +  CGCTTATCT     - 1.0774410774410774              Ratio: Mouse                           cgcttatct Rat                             cgcttatct Human                           ccctaaccg Dog                             ccctaaccg                                   \* \*\* \* \*    CSCS: 0.15514965635112404   - Gnb3                   44     53  -  CTCTTATGC     - 2.4962406015037595              Ratio: Mouse                           ctcttatgc Rat                             --------- Human                           --------- Dog                             --------- Opossum                         ---------  CSCS: 2.7296416491897704   - ENSMUSG00000029821     49     58  +  CTCTTATCA   - ENSMUSG00000028920   -153   -144  -  CTCTTATCA   - Opn1sw               -155   -146  +  CTCTTATCC     - 0.5128205128205128              Ratio: Mouse                           ggataagag- Rat                             ggataagag- Human                           ggataagag- Dog                             ggataaaaat                                   \*\*\*\*\*\* \*     CSCS: -0.7965214354856055   - Opn1sw                 19     28  -  CTCTTATAG     - 0.20833333333333331             Ratio: Mouse                           ctcttatag Rat                             ctcttatgg Human                           ctcttatag Dog                             ctcttatag Opossum                         ctcttatag                                   \*\*\*  \* \*   CSCS: -1.9607435941396139   - Gngt2                 -78    -69  -  GACTTATCT     - 0.41203703703703703             Ratio: Mouse                           agataagtc Rat                             agataagtc Human                           agatccgtc Dog                             agaccagtc                                   \*\*\*   \*\*\*   CSCS: -0.8408718747010308   - ENSMUSG00000029415   -135   -126  -  TCCTTATCT   - ID: cnga3\_317\_327\_2     R|C/ N: (5/9)     Z: 4.050124    Consensus:                           SWCTTATCWSN   - Gnb3                  -22    -11  +  CTCTTATCTCT   - Arr3                  -45    -34  -  CTCTTATATCC     - 0.8221343873517787              Ratio: Mouse                           ggatataagag Rat                             ggatataagag Human                           gggtataagag Dog                             aagtataaaag Opossum                         ggccataaaag                                   \*\*\*\* \*\*   CSCS: -0.43442689109224836   - Gngt2                 -80    -69  -  GACTTATCTCC     - 0.33712121212121215             Ratio: Mouse                           ggagataagtc Rat                             ggagataagtc Human                           ggagatccgtc Dog                             ggagaccagtc                                   \*\*\*\*\*   \*\*\*   CSCS: -1.0025252635162885   - Smug1                 106    117  +  CACTTATCTCA   - ENSMUSG00000029821     49     60  +  CTCTTATCAGT   - ENSMUSG00000026983    160    171  -  TCCTTATCTCT   - ENSMUSG00000028920   -155   -144  -  CTCTTATCAGG   - ENSMUSG00000029415   -137   -126  -  TCCTTATCTCA   - Opn1sw               -155   -144  +  CTCTTATCCTC     - 0.4195804195804196              Ratio: Mouse                           gaggataagag- Rat                             gaggataagag- Human                           gaggataagag- Dog                             gaggataaaaat                                   \*\*\*\*\*\*\*\* \*     CSCS: -1.0088249982463358   - ID: cnga3\_317\_326\_2     R|C/ N: (5/9)     Z: 4.050124    Consensus:                           SWCTTATCWS   - Gnb3                  -22    -12  +  CTCTTATCTC   - Arr3                  -44    -34  -  CTCTTATATC     - 0.7913043478260869              Ratio: Mouse                           gatataagag Rat                             gatataagag Human                           ggtataagag Dog                             agtataaaag Opossum                         gccataaaag                                   \*\*\*\* \*\*   CSCS: -0.4869348110566958   - Smug1                 106    116  +  CACTTATCTC   - ENSMUSG00000029821     49     59  +  CTCTTATCAG   - ENSMUSG00000026983    161    171  -  TCCTTATCTC   - ENSMUSG00000028920   -154   -144  -  CTCTTATCAG   - Gngt2                 -79    -69  -  GACTTATCTC     - 0.37083333333333335             Ratio: Mouse                           gagataagtc Rat                             gagataagtc Human                           gagatccgtc Dog                             gagaccagtc                                   \*\*\*\*   \*\*\*   CSCS: -0.9288965759706529   - ENSMUSG00000029415   -136   -126  -  TCCTTATCTC   - Opn1sw               -155   -145  +  CTCTTATCCT     - 0.46153846153846156             Ratio: Mouse                           aggataagag- Rat                             aggataagag- Human                           aggataagag- Dog                             aggataaaaat                                   \*\*\*\*\*\*\* \*     CSCS: -0.9148097947734568 |

Page by: Charles Danko & Maochun Qin; SUNY Upstate Medical University.
