# Supplementary material for: Bioinformatic identification of novel putative photoreceptor specific cis-elements
Source: BMC Bioinformatics. 2007 Oct 22;8:407. doi: 10.1186/1471-2105-8-407 (PMC2225425; doi:10.1186/1471-2105-8-407)
Supplement: Additional file 2 — Extended table of information on predictions. Contains cross-promoter alignments and phylogenetic alignments for each prediction, as well as the entire list of ENSEMBL IDs for genes used in the study. Please refer to "Data Supplement Instructions.doc" for detailed information. [file 1471-2105-8-407-S2.DOC]

**Bioinformatic Identification of Novel Putative Photoreceptor Specific *cis*-Elements**

*Explanation of Supplementary Data*

**Directions for viewing the HTML-formatted supplementary files.**

Download and uncompress the zip file in a directory on your local machine. Open any of the “.html” files using the Firefox web-browser. Note that javascript must be enabled to view the files properly.

Motifs are sorted into separate boxes. The motif ID, enrichment ratio, Z-score of enrichment, and IUPAC consensus are given for each motif. Occurrences of the motif in the promoter sequences are shown in the gray box. Rod- or cone-specific promoters are marked with the MGI ID for the gene of the applicable promoter, whereas background promoters carry the ENSEMBL gene ID. Next, columns give the start and end position relative to the transcription start site, the strand, and the actual sequence from which the motif was discovered.

Selecting **[more]** on the far-right hand side of each occurrence reveals the phylogenetic alignment corresponding to the above occurrence. Phylogenetic alignments were extracted from whole genome alignments available on the UCSC genome browser. Alignments were not modified in any way, and consequently are presented in the same strand as the chromosome (therefore, the phylogenetic sequence given may be the reverse complement of the motif). Certain motifs fall in adjoining portions between two separate alignments. In these rare cases, the Z-score refers to the second alignment given.

**Additional methods.**

In addition to the tissue-non-specific non-photoreceptor background set discussed in the article, we also ran the same analysis on a separate tissue-specific, non-photoreceptor background set. To identify the genes in this set, we used the same procedure as described in the *Methods* section of the article (“*Selecting background promoter set*”), except that we took all REFSEQ IDs with a tissue-specificity ratio >0.4 (n=62). For simplicity, we discuss only the larger background dataset (non-tissue-specific) in the article; however, data obtained using both background sets are provided in the supplementary data package.

**Included files:**

The following files include occurrence and phylogenetic information for all motif highlighted in the discussion section of the paper. Files include motifs detected using both the non-tissue-specific (NS) and tissue-specific (TS) background sets.

EN2.html: Motifs similar to the central region of an Engrailed homeodomain.

IL6.html: Motifs similar to the binding site for IL-6 effectors.

INR.html: Motifs similar in base composition and position to INR elements.

NRE.html: Motifs similar to NRE.

ROP2.html: Motif similar to ROP2.

RXR.html: Motifs similar to the binding site for RXR family members.

TATAlike.html: Motifs similar to the central region of a TATA-box; mean position matches expected TATA position with high variance.

The following files include all predictions for rods (r.*) and cones (c.*) presented in Table 3.

r.NS.html

c.NS.html

The following files are the same as the two immediately above, except using the tissue-specific background set.

r.TS.html

c.TS.html

The following supplementary files do not contain data, but they are required for the web browser to display the data files properly.

dropdown.css

dropdown.js

The following files list the ENSEMBL IDs for genes in the non-tissue-specific (NS) and tissue-specific (TS) non-photoreceptor background set.

NS.names

TS.names
