# Supplementary figures and images for: Bioinformatic identification of novel putative photoreceptor specific cis-elements
Source: BMC Bioinformatics. 2007 Oct 22;8:407. doi: 10.1186/1471-2105-8-407 (PMC2225425; doi:10.1186/1471-2105-8-407)

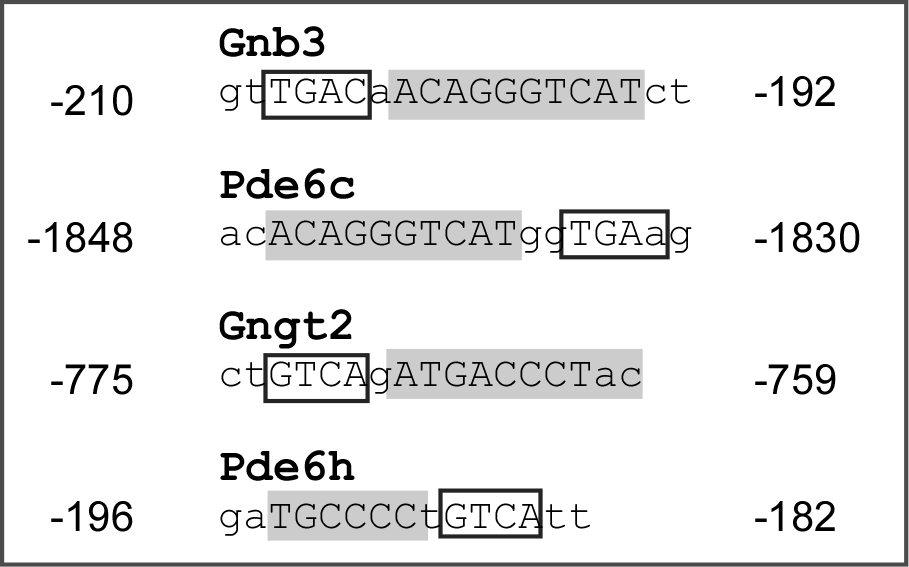

Supplement: Additional file 3 — Additional figure 1. Region surrounding predicted IL-6 sites in 5 rod promoters. Sequences identified by IAMMS are shaded in gray; copies of the core (including the degenerate copy CTGGA) are outlined in black. [file 1471-2105-8-407-S3.png]

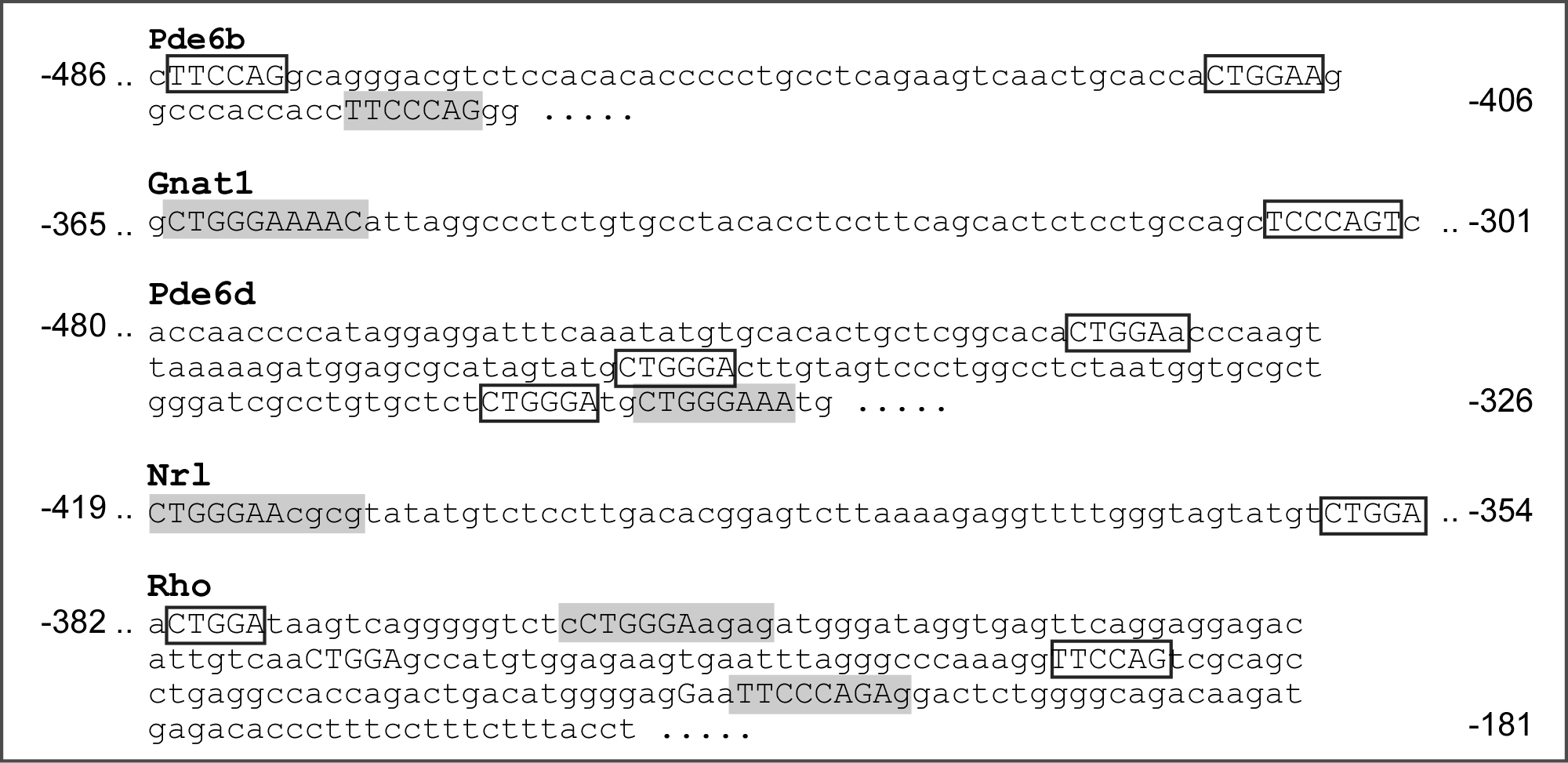

Supplement: Additional file 4 — Additional figure 2. The location of predicted RXR core binding sites (gray) and the adjacent degenerate core region (outline) in 4 cone promoters. [file 1471-2105-8-407-S4.png]
